# Systematic review of wastewater surveillance of antimicrobial resistance in human populations

**DOI:** 10.1016/j.envint.2022.107171

**Published:** 2022-04

**Authors:** K.K. Chau, L. Barker, E.P. Budgell, K.D. Vihta, N. Sims, B. Kasprzyk-Hordern, E. Harriss, D.W. Crook, D.S. Read, A.S. Walker, N. Stoesser

**Affiliations:** a Nuffield Department of Medicine, John Radcliffe Hospital, Oxford OX3 9DU, United Kingdom; b Department of Chemistry, Faculty of Science, University of Bath, Bath BA2 7AY, United Kingdom; c Bodleian Healthcare Libraries, University of Oxford, Oxford OX3 9DU, United Kingdom; d Department of Microbiology/Infectious Diseases, Oxford University Hospitals NHS Foundation Trust, John Radcliffe Hospital, Oxford OX3 9DU, United Kingdom; e UK Centre for Ecology & Hydrology, Wallingford OX10 8BB, United Kingdom; f NIHR Oxford Biomedical Research Centre, Oxford OX4 2PG, United Kingdom

**Keywords:** AMR, Sewage, Epidemiology, Wastewater, Surveillance, AST, Antimicrobial susceptibility testing, CIA, Critically important antimicrobial, ESBL, Extended spectrum beta-lactamase, IQR, Interquartile range, LMIC, Lower-middle-income country, MDR, Multidrug resistant, PE, Population equivalent, WGS, Whole genome sequencing, WWTW, Wastewater treatment works

## Abstract

**Objectives:**

We systematically reviewed studies using wastewater for AMR surveillance in human populations, to determine: (i) evidence of concordance between wastewater-human AMR prevalence estimates, and (ii) methodological approaches which optimised identifying such an association, and which could be recommended as standard. We used Lin’s concordance correlation coefficient (CCC) to quantify concordance between AMR prevalence estimates in wastewater and human compartments (where CCC = 1 reflects perfect concordance), and logistic regression to identify study features (e.g. sampling methods) associated with high agreement studies (defined as >70% of within-study wastewater-human AMR prevalence comparisons within ±10%).

**Results:**

Of 8,867 records and 441 full-text methods reviewed, 33 studies were included. AMR prevalence data was extractable from 24 studies conducting phenotypic-only (n = 7), genotypic-only (n = 1) or combined (n = 16) AMR detection. Overall concordance of wastewater-human AMR prevalence estimates was reasonably high for both phenotypic (CCC = 0.85 [95% CI 0.8–0.89]) and genotypic approaches (CCC = 0.88 (95% CI 0.84–0.9)) despite diverse study designs, bacterial species investigated and phenotypic/genotypic targets. No significant relationships between methodological approaches and high agreement studies were identified using logistic regression; however, this was limited by inconsistent reporting of study features, significant heterogeneity in approaches and limited sample size. Based on a secondary, descriptive synthesis, studies conducting composite sampling of wastewater influent, longitudinal sampling >12 months, and time-/location-matched sampling of wastewater and human compartments generally had higher agreement.

**Conclusion:**

Wastewater-based surveillance of AMR appears promising, with high overall concordance between wastewater and human AMR prevalence estimates in studies irrespective of heterogenous approaches. However, our review suggests future work would benefit from: time-/location-matched sampling of wastewater and human populations, composite sampling of influent, and sampling >12 months for longitudinal studies. Further research and clear and consistent reporting of study methods is required to identify optimal practice.

## Highlights

• Detection of wastewater-human AMR associations is promising despite diverse methods.
• Including composite influent, 12-month timeframe and WwTW-human overlap appears optimal.
• Specific species and AMR mechanisms appear more suited to the approach than others.
• Genomic approaches facilitate agnostic detection and biostatistical synthesis.
• More studies with clear reporting are needed to validate “best practice” protocols.

## 1 Introduction

Antimicrobial resistance (AMR) is a significant threat to global health (O’Neill, 2016) and a multi-faceted problem compounded by diverse drivers facilitating its emergence and spread. AMR surveillance is critical to understanding trends, monitoring interventions and developing empiric treatment guidelines, as prioritised in the World Health Organisation’s global AMR action plan (WHO, 2019). Large networks sharing AMR data have been established to meet this need, including the European Antimicrobial Resistance Surveillance Network (EARS-Net) and the Global Antimicrobial Resistance Surveillance System (GLASS). However, current surveillance can be limited by the reliance on individual-level sampling, which is often affected by selection bias towards healthcare-associated settings (WHO, 2018). For example, both EARS-Net and GLASS target AMR in clinical specimens from hospitalised patients; this however does not reliably capture AMR prevalence in commensal organisms, thought to silently constitute most of the true AMR burden (Fahrenfeld and Bisceglia, 2016, Hay et al., 2018, Hendriksen et al., 2019b). Additionally, data collection is often limited to a subset of culturable species, and on susceptibility phenotypes rather than AMR genotypes. This lack of genotyping hampers the surveillance of high-risk AMR-associated clones and specific AMR-associated genetic determinants (Tacconelli et al., 2018). In many settings, particularly low- and middle-income countries (LMICs) where the burden of AMR is largest, the laboratory infrastructure to support individual, patient-level surveillance is lacking.

Wastewater-based epidemiology (WBE) is an epidemiological approach based on the analyses of wastewater (e.g. sewage) to generate information on human populations on a community scale (Choi et al., 2018). WBE has the potential to overcome some of the aforementioned challenges by simultaneously sampling both healthcare- and community-associated populations at scale (Newton et al., 2015). The approach has already been successful in illicit drug monitoring (González-Mariño et al., 2020) and pathogen surveillance (Asghar et al., 2014, Fernández et al., 2012), including SARS-CoV-2 (Ahmed et al., 2020a, Ahmed et al., 2020b), and its application to AMR surveillance is gaining traction (Fahrenfeld and Bisceglia, 2016). Difficulties in standardising AMR detection methods and targets across surveillance networks (Tacconelli et al., 2018) could potentially be circumvented by using metagenomics to agnostically probe wastewater resistomes (Aarestrup and Woolhouse, 2020, Wright, 2007). Recent wastewater-based studies have investigated seasonal/geographic AMR distributions (Su et al., 2017), quantified global AMR gene abundance (Hendriksen et al., 2019b) and identified associations between AMR in wastewater and clinical samples (Karkman et al., 2020, Pärnänen et al., 2019). However, heterogeneous study designs and methods likely contribute to differences in outcomes/interpretations. The impact of methodological approaches such as grab sampling (i.e. taking single samples at a single timepoint) (Reinthaler et al., 2013), snapshot versus longitudinal study design, sampling in the presence of unrepresentative and “contaminating” AMR-associated point sources, and/or characterising AMR based on phenotypic testing of isolates versus genotypic profiling remains poorly understood (Table 1).

**Table 1:** Methodological features potentially contributing to variability in outcomes.

**Methodological features**	**Examples of methodological feature**	**Aspects potentially introducing variability in outcomes**	**References**
Wastewater sampling point type	Wastewater treatment works (WWTW) sampling point e.g. influent versus effluent	Treatment processes can transform microbial and AMR composition resulting in differences between e.g. influent and effluent samples	Tong et al., 2019, Zhang et al., 2020
	• Hospital effluent • Domestic sewers/manholes	Focussed sampling may only represent specific sub-populations*	Jakobsen et al., 2008, Larson et al., 2020*
	Informal sewer systems	Informal sewer systems (often with low flow) may be susceptible to homogeneity	Fahrenfeld and Bisceglia, 2016

Wastewater sampling method	Grab (single sample)	• Single grab samples can be flooded by homogenous solids • Wastewater composition can vary significantly over short time periods	Reinthaler et al., 2013, Guo et al., 2019
	• Composite sampling (combining grabs) • Proportional sampling (flow/time/volume)	Composite and proportional samples capture average composition but may be unable to discriminate peak values during sampling period*	Michael-kordatou et al., 2020*

WWTW sewershed inputs	• Hospitals • Other healthcare facilities (e.g. care homes) • Industry • Abattoirs	• Effluent from AMR-associated sources may obscure detection of true population-level trends (e.g. elevated levels of unique AMR, co-selection of plasmids, non-human associated AMR)* • Unique substrate properties of industry effluent can influence WWTW communities	Jakobsen et al., 2008, Shchegolkova et al., 2016, Larson et al., 2020*
	• Agriculture • Rural communities	Subsistence farming and inadequate waste management can result in frequent contact between human and environmental reservoirs	Pehrsson et al., 2016

WWTW properties and sampling conditions	Size of WWTW sewershed and infrastructure	• Long conveyance times from population to sampling point may impact composition due to transformation in unique sewer environment (anaerobic, temperature, biofilms) • Presence of pre-treatment infrastructure (e.g. pumping stations, balancing tanks) may also play a role in transforming wastewater	Fahrenfeld and Bisceglia, 2016
	Treatment methods	When sampling treated wastewater, differing levels of treatment can selectively transform AMR and microbial composition	Tong et al., 2019, Zhang et al., 2020
	Geography and weather (seasons, rainfall, temperature, latitude)	• Heavy rainfall dilutes wastewater in combined sewer systems via rainwater runoff and by infiltration of groundwater (dislodged biofilms, freshwater taxa) • Local ambient environment and climate can influence both human-associated microorganisms entering the system and resident sewer microbiota	Shanks et al., 2013
	Flow rate	• Combined sewer overflows impact composition of post-treatment samples collected during these events • Flow rate is also associated to hydraulic retention time and the level of WWTW treatment	Honda et al., 2020

Sample processing methods	• Filtration • Storage conditions • Sodium thiosulfate (chlorine neutraliser)	Different sample processing methods may selectively affect recovery yields of specific species*	Ahmed et al., 2020*
	Freeze-thaw cycles	Multiple freeze-thaw cycles shown to select for Firmicutes, Actinobacteria, and eukaryotic microorganisms	Poulsen et al., 2021
	DNA extraction methods	Use of different metagenomic DNA extraction kits and procedures has been shown to modulate inferred microbial composition*	Knudsen et al., 2016, Michael-Kordatou et al., 2020*

AMR detection methods	• Culture-based and phenotypic (selective media, disk-diffusion, microbroth dilution) • Culture-based and genotypic (WGS, PCR, qPCR) • Direct-from-sample genotypic (qPCR, metagenomics)	Methods based on culturing isolates may only capture a fraction of the diversity present even with detailed sampling	Shaw et al., 2021
		Culture-based methods may be subject to variations from phenotyping method and breakpoint selection	Davies, 2019, Davies et al., 2020
		Bioinformatic deconvolution can be subject to variation depending on which tools/databases/references are used.	Lal Gupta et al., 2020

* Also recently studied as sources of variability in outcomes for SARS-CoV-2 wastewater-based epidemiology and likely relevant for AMR surveillance.

Despite the increasing use of WBE for AMR surveillance/evaluation purposes, there has been no attempt to review the available data, synthesise the evidence, and assess remaining knowledge gaps. We therefore systematically reviewed studies using wastewater (i.e. the “wastewater compartment”) for AMR surveillance in human populations (i.e. the “human compartment”), seeking firstly to characterise the strength of the AMR prevalence associations observed between wastewater and human compartments to identify whether this appears to be a promising surveillance approach. Secondly, we sought to identify methodological factors that might optimise these associations in support of a standardised approach for wastewater-based AMR surveillance going forwards. We specifically focussed on study design and methodological approaches, including AMR detection methods, highlighting limitations and recommendations for future work.

## 2 Materials and methods

For this systematic review, we sought firstly to evaluate concordance between wastewater and human AMR prevalence estimates for each study, stratified by the AMR detection method used (i.e. phenotypic versus genotypic). Secondly, we adapted the PECOTS (Population, Exposure/Intervention, Comparator, Outcome, Target Condition, Study Design) systematic review framework and formulated the following statement to assess association between study methods and outcomes: Among studies jointly evaluating AMR prevalence in wastewater and humans, what is the effect of methodological approaches (e.g. wastewater sampling methods, AMR detection methods) on the concordance between these metrics?

A PRISMA (Preferred Reporting Items for Systematic Reviews and Meta-Analyses) checklist is included in (Supplementary data**set 1),** and the complete PROSPERO (International prospective register of systematic reviews) protocol is available at: https://www.crd.york.ac.uk/prospero/display_record.php?ID=CRD42019134946.

### 2.1 Literature search

The search string was developed through iterative preliminary searches in consultation with a librarian experienced with systematic reviews. Full search strings adapted for each database are presented in (Supplementary data**set 2**). Searches were conducted on 01/02/2019 in: MEDLINE (National Library of Medicine), EMBASE (Excerpta Medica dataBASE) Global Health, 2021, CAB Abstracts, 2021, SCOPUS, 2021, Web of Science Core Collection, 2021. Searches were updated on 09/01/2021 using identical search strings. Results were limited to the English language and de-duplicated.

### 2.2 Eligibility criteria

Records were assessed through a two-stage screen detailed in (Fig.S1**,** Supplementary data**set 3**) to capture both studies piloting wastewater-based AMR surveillance and studies conducting relevant wastewater-human AMR comparisons. Briefly, stage one screened titles/abstracts to determine if the study was: (i) primary research conducted by the author/s, (ii) investigated wastewater which at least in part was constituted of human waste, (iii) reported AMR prevalence as a result of the work/analyses undertaken, and (iv) performed comparative analyses of the investigated wastewater to a separate non-wastewater compartment which potentially represents AMR prevalence in a human population. If it was unclear whether a study met criteria based on title and abstract alone, the study was passed onto the next stage. Stage two reviewed full-text methods and studies included if: (i) the wastewater analysed originated from at least one conventional WWTW where multiple waste streams converge, and (ii) the compartment being used as a comparator to wastewater AMR prevalence must directly represent a human population such as in clinical isolates or resistance network data. A universal inclusion question was used to include studies explicitly performing wastewater-based surveillance of human AMR irrespective of meeting stage one or two criteria. For full descriptions of screening criteria and examples of excluded records see (Fig.S1**,** Supplementary data**set 3**).

### 2.3 Study selection and data extraction

Records were independently screened in duplicate (by the authors KKC and LB) and data was extracted from included records using a pre-tested data extraction form piloted on five random included records (Supplementary data**set 4**). This form consisted of both pre-determined fields using data validation and non-restricted write in fields to record data relating to (non-exhaustive): study design (wastewater sampling strategy, WWTW metadata, human sample type, sample sizes), methods (wastewater sampling, AMR detection, statistical methods) and outcomes (reported wastewater-human comparison results). If available, raw resistance prevalence data (total and resistant isolate counts regarding individual antibiotics or resistance genes) were extracted for antibiotics on the WHO critically important antimicrobials (CIAs) list (AGISAR, 2018). These counts were used to calculate point estimates (±95% confidence intervals) representing the proportion of AMR isolates amongst all isolates tested for either wastewater or human compartments; this metric is referred to as either the wastewater or human AMR prevalence from here on.

### 2.4 Risk of bias and certainty assessment

Risk of bias was assessed independently by two reviewers (the authors KKC and LB) using a qualitative approach based on the Cochrane risk of bias tool (Higgins et al., 2011); our modified tool focused on systematic differences at the study level as outcomes reported were highly diverse. Modified interpretations of five bias domains are detailed in **Table S2**; a brief summary of our interpretation of each bias domain is provided here. Attrition bias referred to differences introduced by missing data (e.g. missing sampling timepoints in longitudinal studies). Performance bias referred to differences introduced by use of different methods between or within sampling compartments (i.e. AMR detection methods). Reporting bias referred to selective reporting where outcomes were measured but not reported or disproportionately reported in the text. Selection bias referred to differences introduced across the wastewater compartment by use of different wastewater sampling or processing methods (e.g. selecting more colonies for susceptibility testing from one WWTW compared to another). Other bias referred to a lack of acknowledgement of AMR-influencing wastewater inputs (i.e. unreported sewer inputs from healthcare, abattoir or agricultural sources), which may have modulated AMR profiles in sampled wastewater. If there was insufficient information present to assess the risk of bias this was denoted as “unclear”. The rationale for risk of bias assessments was recorded, including examples where applicable. Discrepancies in risk of bias assessments were resolved by discussion. An overall qualitative measure (high, low and unclear) was assigned to each study as per the Cochrane risk of bias tool approach to summary assessment (Higgins et al., 2011).

Certainty assessment (assessment of overall confidence in the evidence included) was conducted using an adaptation (Woodruff and Sutton, 2011) of the GRADE (Grading of Recommendations, Assessment, Development and Evaluations) system designed for clinical studies (Morgan et al., 2016), where evidence is given an initial rating (“high”, “moderate”, “low”, “very low”) then upgraded or downgraded based on study characteristics. The outcome being evaluated was whether or not high concordance between AMR prevalence estimates in wastewater and human compartments was observed. Conventionally, GRADE assigns “high” and “low” initial ratings to randomised trials and observational studies respectively. A randomised controlled trial design is of limited applicability to the studies being evaluated as part of this review, and therefore using the adapted version of GRADE (Woodruff and Sutton, 2011), we assigned initial “moderate” ratings to our body of evidence. Upgrading or downgrading was based on a subset of adapted GRADE domains (**Table S3**). Briefly, these included our risk of bias summary assessment, inconsistency, indirectness, imprecision, publication bias and concordance (all defined in **Table S3**). In our adaptations we omitted two of the original GRADE criteria (“large effects”, “residual confounding”), as these could not be readily adapted to our context.

### 2.5 Data synthesis and analysis

For extracted resistance prevalence data, we used Lin’s concordance correlation coefficient (CCC – R package DescTools) with 95% confidence intervals (CIs) to quantify the concordance between the proportion of resistant wastewater isolates (i.e. wastewater AMR prevalence) and the proportion of resistant human isolates (i.e. human AMR prevalence), with the latter representing the reference standard. As perfect concordance is unrealistic, we arbitrarily defined “high concordance” to represent a ±10% difference in AMR prevalence between wastewater and human compartments. Concordance was considered separately for comparisons derived from phenotypic versus genotypic approaches. In addition to evaluating concordance across all studies, we also evaluated concordance stratified by bacterial species, resistance to specific antibiotic classes and for AMR gene families. Since Lin’s CCC does not reflect error in AMR prevalence estimates, we also compared Clopper-Pearson 95% confidence intervals (CIs) stratified by study, antibiotic class or AMR gene.

As we aimed to identify approaches that could optimise wastewater-based AMR surveillance, we classified studies based on wastewater-human AMR concordance where a “high agreement” study was defined as a study with >70% of its wastewater and human AMR prevalence estimate comparisons being within ±10% of each other (i.e. highly concordant comparisons, see above). We then used logistic regression to identify if any study features were associated with this high agreement classification in STATA/IC v.16.1 (StataCorp, College Station, USA). As study feature reporting was highly inconsistent, we only tested features that were reported by at least 75% of studies.

In addition, given the heterogeneity of study features, their inconsistent reporting across studies, and the small number of studies limiting power to detect associations, we descriptively synthesised features potentially associated with “high agreement” studies (i.e. where >70% of wastewater and human AMR prevalence estimate comparisons were within ±10% of each other). For this, in addition to the high agreement category, we further classified studies as moderate agreement (30–70% of wastewater and human AMR prevalence estimate comparisons being within ±10% of each other) and low agreement studies (<30% of wastewater and human AMR prevalence estimate comparisons being within ±10% of each other). Agreement classifications were considered separately for comparisons derived from phenotypic versus genotypic approaches.

## 3 Results

### 3.1 Study selection, risk of bias and characteristics

#### 3.1.1 Literature screen

**Fig. 1:**
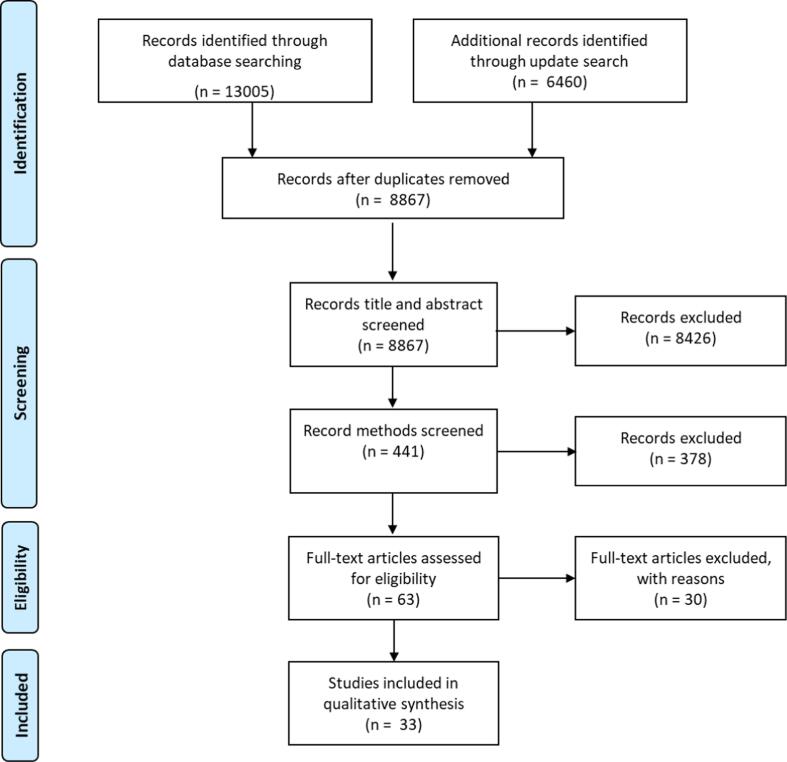
PRISMA flowchart of search strategy and study inclusion/exclusions.

Of 8,867 de-duplicated studies identified using our search strategy, full-text methods for 441 relevant studies were reviewed, and based on pre-specified inclusion criteria (see Methods), 33 studies were included in the review (Fig. 1 **and** Fig.S1).

#### 3.1.2 Risk of bias and certainty assessment

Based on our modified bias domains (**Table S2**), 19/33 studies were judged to have overall high-risk of bias, 7/33 with an unclear-risk and 7/33 with low-risk (Supplementary data**set 5**). To avoid splitting our analyses and losing statistical power, we present a synthesis of all studies and provide a summary of the risk of bias across studies below (as recommended by Higgins et al., 2011 when the study pool is small and stratified analyses according to risk categories is not feasible).

Selection bias was present in eight studies (Haghi et al., 2019, Hendriksen et al., 2019b, Jakobsen et al., 2008, Karkman et al., 2020, Moradigaravand et al., 2018, Pignato et al., 2010, Reinthaler et al., 2013, YoungKeun et al., 2015) where 7/8 sampled multiple wastewater sources using different sampling methods. Performance bias was not present in any studies, with laboratory methods consistent within studies. Attrition bias occurred in three studies as missing pre-specified sampling timepoints due to logistical factors (e.g. WWTW closure, problems with sampling permissions (Pärnänen et al., 2019), or missing datapoints (Jørgensen et al., 2017, Urase et al., 2020). Reporting bias was present in three studies where sample sizes or antibiotic phenotyping data were incompletely reported for one or more group (Colomer-Lluch et al., 2014, Meir-Gruber et al., 2016, YoungKeun et al., 2015). Other bias was evident in six studies (Rahimi and Bouzari, 2015, Haghi et al., 2019; Mourkas et al., 2019; Ojer-Usoz et al., 2017, Pehrsson et al., 2016, Saifi et al., 2009) which did not report any information regarding sewer inputs to sampled wastewater.

Based on our certainty assessment, we rated the overall quality of included bodies of evidence as “low to moderate” regarding the outcome of identifying concordance between wastewater and human AMR prevalence estimates (**Table S4**). Initial “moderate” ratings were downgraded due to high overall risk of bias and the indirectness of study aims in the context of our review questions. Upgrades were warranted by statistically significant concordance (adapted GRADE “dose-response” assessment) across most studies resulting in the final “low to moderate” rating of the evidence base on which the following data synthesis and recommendations are made.

#### 3.1.3 Summary of general study characteristics

**Fig. 2:**
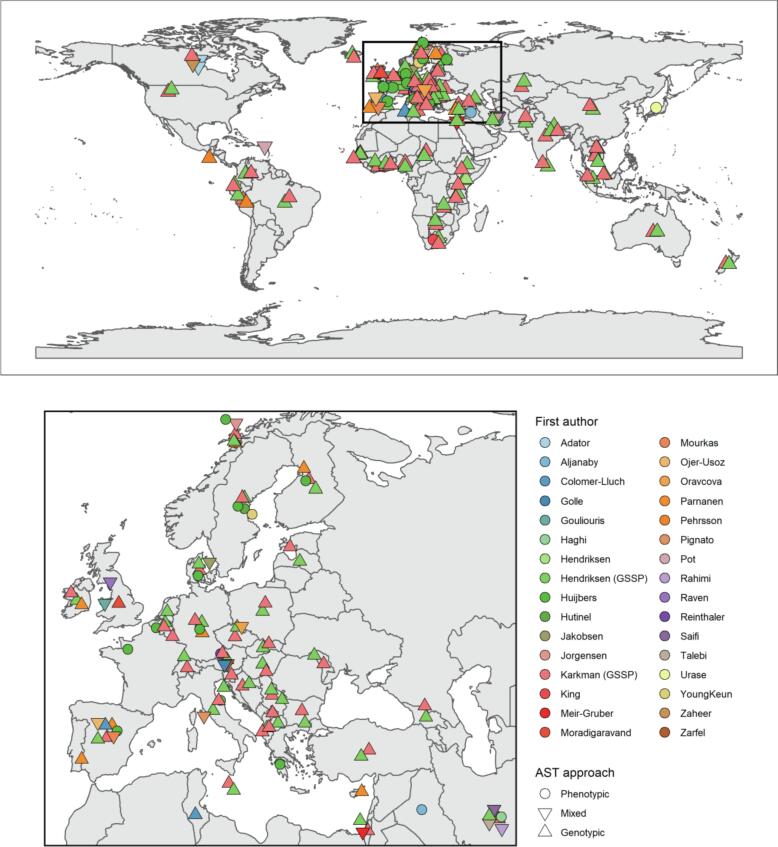
Geographic distribution of wastewater sampling and test approach of included studies. Centroids of countries sampled by included studies are plotted with colours and shapes according to citation and antimicrobial susceptibility test (AST) approach respectively. Centroid are plotted with jitter to avoid overplotting and do not represent exact sampling locations within countries.

Studies explicitly using wastewater for human population-level AMR surveillance made up 12/33 included studies. The remaining 21 studies included relevant comparisons between wastewater and human AMR, but were not directly set up as wastewater-based AMR surveillance studies (e.g. studies focussed on One Health). Amongst the 33 included studies, 73 unique countries were sampled, although most (48/73) were represented as part of a single global study (Hendriksen et al., 2019b) (Fig. 2). World Bank regions covered by the studies were as follows: East Asia and Pacific (n = 3 studies), Europe and Central Asia (n = 19), Latin America and the Caribbean (n = 4), Middle East and North Africa (n = 9), North America (n = 5), South Asia (n = 2), Sub-Saharan Africa (n = 4). World Bank income classifications showed a sampling skew towards high-income countries (high income [n = 24 studies]; middle income [n = 11] and low income [n = 3]) (Fig.S5). Three studies covered multiple regions and income classifications. Publication dates ranged from 2007 to 2020, with most published in the last three years (n = 24) (for full study descriptions, see Appendix A, Adator et al., 2020a, Aljanaby, 2018, King et al., 2020, Oravcova et al., 2017, Pot et al., 2020).

#### 3.1.4 Summary of AMR detection methods in included studies

Evaluations of AMR were undertaken using genotypic-only methods (n = 7), phenotypic-only methods (n = 8), or a mixed approach combining both (n = 18). Genotypic-only studies employed metagenomics (n = 4), qPCR (n = 2) and single isolate whole genome sequencing (WGS) (n = 1). Phenotypic-only studies employed disk-diffusion (n = 4), microbroth dilution (n = 3), or both (n = 1). Mixed approach studies combined disk-diffusion/microbroth dilution with qPCR (n = 1), PCR (n = 9) or single isolate WGS (n = 8). For data synthesis and analysis across studies, relevant phenotypic data was extracted from 22 studies and genotypic data from 12 studies (24 studies in total as both data types could be extracted from 10 studies). These 24 studies conducted phenotypic-only (n = 7), genotypic-only (n = 1) or combined (n = 16) AMR detection. All extracted genotypic data consisted of isolate-level qPCR, PCR or WGS; no metagenomic data at the sample-level was synthesised. For nine studies there were no relevant data that could be extracted for inclusion in a combined summary; these were either where isolate counts were not reported, or only raw sequencing data was available which was beyond the scope of our analysis.

### 3.2 Wastewater-human AMR concordance

#### 3.2.1 Phenotypic wastewater-human AMR concordance

**Fig. 3:**
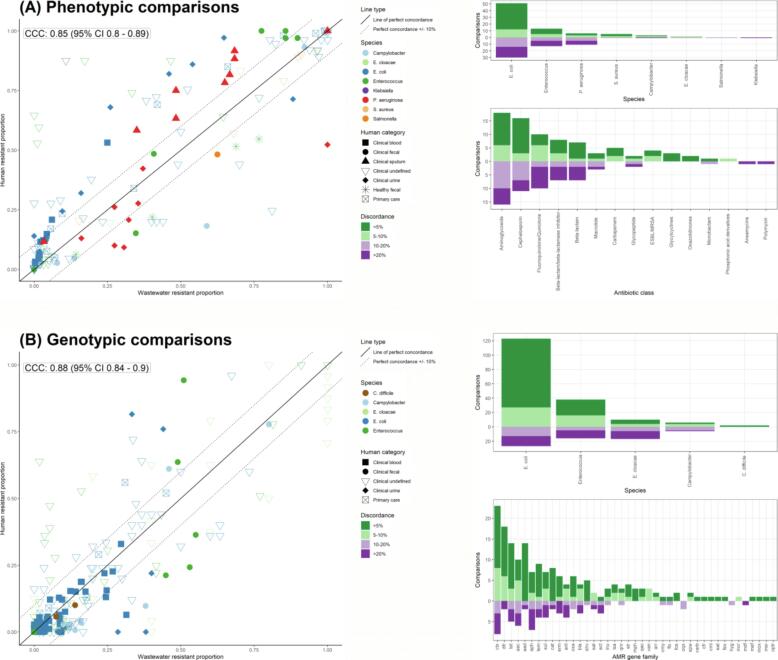
AMR in wastewater isolates and human isolates for phenotypic (A) and genotypic (B) comparisons. Left: Concordance plot of AMR prevalence in wastewater and human isolates stratified by AMR detection approach (i.e. phenotypic (A) versus genotypic (B) approaches). Each point represents a single wastewater-human comparison conducted where colour corresponds to bacterial species tested and shape corresponds to human sample type used. Lin’s concordance correlation coefficient (CCC) is labelled with 95% confidence intervals. Unbroken line of *y* = *x* is plotted as perfect concordance between wastewater and human resistance. Dashed lines of *y = x* + 0.1 and *y =* x-0.1 represent high concordance, i.e. ±10% from perfect concordance respectively. Right: Individual wastewater-human comparisons tallied by level of discordance (<5% and 5–10% coloured in green, 15–20% and >20% coloured in purple) between compared wastewater and human AMR prevalence estimates, and plotted to show number of comparisons at each level of discordance, stratified by the target species and antibiotic class (3A-right) or AMR gene family (3B-right).

Phenotypic data from 22 studies covered fifteen WHO Critically Important Antimicrobial (CIA) classes and comprised 139 comparisons for which overall concordance between resistance prevalence estimates in wastewater and human compartments was reasonably high (CCC = 0.85 [95% CI 0.8–0.89]) (Fig. 3**A**). The median number of comparisons (i.e. AMR prevalence for a specific species-drug across both wastewater and human compartments) per study was 6 (IQR: 3–9). For any comparison, the median number of isolates analysed in humans was 130 (IQR: 50–232), and in wastewater 98 (IQR: 58–186). AMR prevalence estimates were highly concordant (defined as wastewater AMR prevalence within ±10% of human AMR prevalence) for 80/139 (58%) comparisons.

Hutinel et al., 2019, Ojer-Usoz et al., 2017 contributed the most highly concordant comparisons (13/16 [81%] and 14/22 [64%] within-study comparisons respectively); however only the Hutinel study was therefore defined as a “high agreement” study. Most phenotypic comparisons utilised isolates cultured from human samples originating from healthcare settings (135/139 comparisons), with higher AMR prevalence than corresponding wastewater estimates (in 86/135 (64%) comparisons). The remaining four comparisons (all from Haghi et al. 2019) were unique in comparing fecal isolates from healthy volunteers with no recent history of antibiotic use to wastewater; all four showed reduced AMR prevalence in the human isolates. Sensitivity analysis using data from low bias studies only (n = 5; 31 comparisons) showed a slight decrease in overall concordance (CCC = 0.81 [95% CI 0.65–0.9]; 95% CI overlaps with that of the full phenotypic dataset described above) (Fig.S6).

The most common species and antibiotic class investigated using phenotypic susceptibilities were *E. coli* and aminoglycosides respectively. When considering discordance between wastewater and human AMR prevalence estimates across the top two represented species, 38/81 (47%) comparisons for *E. coli* showed <5% discordance and 51/81 (63%) of comparisons ≤10% discordance, whereas 8/26 (31%) of comparisons across compartments in *Enterococcus* spp. showed < 5% discordance and 13/26 (50%) ≤ 10% discordance (Fig. 3**A, top right panel**). Differences between the top antibiotic classes evaluated were similarly seen; those investigating aminoglycoside resistance showed <5% discordance for 11/34 (32%) and ≤10% discordance for 18/34 (53%) of comparisons, while cephalosporin resistance comparisons showed <5% discordance for 13/27 (48%) and ≤10% discordance for 16/27 (59%) (Fig. 3**A, bottom right panel**).

By individual WHO CIAs, AMR prevalence estimates (i.e. point estimate [95% confidence intervals]) in human and wastewater isolates overlapped for: (i) aminoglycosides (26/34 comparisons; Fig.S7; (ii) beta-lactams (10/14 comparisons; Fig.S8); (iii) beta-lactam/beta-lactamase inhibitor combinations (8/15 comparisons; Fig.S9); (iv) cephalosporins (18/26 comparisons; Fig.S10); (v) carbapenems (5/5 comparison; Fig.S11); (vi) fluoroquinolones/quinolones (7/20 comparisons; Fig.S12); (vii) glycopeptides (3/4 comparisons; Fig.S13); and (viii) erythromycin (5/6 comparisons; Fig.S14). Overlap was also seen specifically for extended-spectrum beta-lactamase (ESBL)-producing isolates (2/3 comparisons) and methicillin-resistant *Staphylococcus aureus* (MRSA) (1/1 comparison; both Fig.S15). Non-overlapping estimates were mostly associated with five studies (Zarfel et al., 2010, Huijbers et al., 2020, Reinthaler et al., 2013, Saifi et al., 2009, Zaheer et al., 2020).

#### 3.2.2 Genotypic wastewater-human AMR concordance

Extracted genotypic data (single isolate WGS and PCR) from 12 studies comprised 245 comparisons between AMR gene prevalence estimates between wastewater and human compartments. Correlation between compartments was slightly higher than for phenotypic data (CCC = 0.88 (95% CI 0.84–0.9)) and overall spread away from perfect concordance was reduced (Fig. 3**B**), with high concordance (wastewater AMR prevalence within ±10% of human AMR prevalence) in 179/245 (73%) comparisons. The median number of comparisons (i.e. AMR prevalence for a specific species-AMR gene combination across both wastewater and human compartments) per study was 11 (IQR: 5–23). For any comparison, the median number of isolates analysed in humans was 94 (IQR: 25–437), and in wastewater 56 (IQR: 30–388).

Using genotypic analyses, most high concordance comparisons were observed in Raven et al., 2019 (71/73), followed by Adator et al., 2020b (33/45) and Zaheer et al., 2020 (19/22). All genotypic comparisons evaluated isolates cultured from human samples originating from healthcare settings, with higher AMR prevalence than the corresponding wastewater estimate in 102/245 (42%). Sensitivity analysis of wastewater-human genotypic concordance using data from low bias studies only (n = 4; 154 comparisons) showed a slight increase (CCC = 0.9 [95% CI 0.87–0.93]; confidence interval overlapping the CCC estimate [95% CI] derived from the full genotypic dataset) (Fig.S6).

For genotypic comparisons, the most common species and AMR gene family investigated were *E. coli* and CTX-M β-lactamases respectively. When considering discordance across species, 95/150 (63%) comparisons for *E. coli* showed <5% discordance and 123/145 (85%) of comparisons ≤10% discordance, while comparisons in *Enterococcus* spp. showed 32/54 (59%) <5% discordance and 38/54 (70%) ≤10% discordance. *Enterobacter cloacae and Campylobacter* spp. comparisons shared more even distributions of discordance levels (Fig. 3**B top right panel**). For AMR gene family, comparisons of most gene families across compartments were in high concordance (i.e. ≤10% discordance) such as for CTX-M β-lactamases 23/31 (74%), *dfr* 18/20 (90%) and *aad* 14/16 (88%) (Fig. 3**B bottom right panel**).

For a subset of eight genes conferring resistance to WHO CIAs and investigated by multiple studies, 95% CIs around prevalence estimates in both compartments overlapped as follows: (i) *aac* (13/16 (81%) comparisons; Fig.S16 [displayed for individual allelic variants]); (ii) *arr* (1/2 comparisons; Fig.S17); (iii) CTX-M 25/29 comparisons; Fig.S18); (iv) *erm* (6/9 comparisons; Fig.S19); (v) *fos* (2/2 comparisons; Fig.S20); (vi) *oxa* (8/8 comparisons; Fig.S21); (vii) *van* (3/3 comparisons; Fig.S22) and (viii) *qnr* (3/4 comparisons; Fig.S23).

### 3.3 Synthesis of study features

#### 3.3.1 Logistic regression of study features and wastewater-human AMR agreement

If more than 70% of wastewater-human AMR prevalence estimate comparisons conducted by a study were highly concordant (i.e. prevalence estimates within ±10% of one another), studies were classed as high agreement overall; this was the case for 6/22 studies (27%) with phenotypic data and 5/12 (42%) studies with genotypic data. Of the ten studies with both phenotypic and genotypic data available only 1/10 showed high agreement for both approaches (Adator et al., 2020b); the remaining 9/10 either showed high agreement for only one approach (3/10) or neither approach (6/10). No statistically significant associations between study features and higher wastewater-human AMR concordance were identified (**Table S24**). The limited number of eligible studies, the substantial heterogeneity of combinations of approaches deployed across studies, and missingness of data meant power to detect independent associations was low (**Table S25**).

#### 3.3.2 Study features descriptive synthesis

We therefore synthesised study features descriptively, additionally assigning studies to moderate (30–70% of wastewater and human AMR prevalence estimate comparisons being within ±10% of each other) and low agreement categories (<30% of wastewater and human AMR prevalence estimate comparisons being within ±10% of each other), and treating phenotypic- and genotypic-approaches as separate study subsets **(Table S26).** Sampling of influent, either alone or in conjunction with effluent, appeared most consistently associated with moderate-/high-agreement (15/28; 54% where reported) in estimates of AMR prevalence between wastewater and human compartments**.** Longitudinal sampling was the most common study design (29/32; 91% where reported) with moderate-/high-agreement in most (22/29; 76%). The four studies undertaking snapshot (i.e. single-timepoint) sampling all had moderate-/high-agreement whereas the single study conducting a mixed sampling design was associated with low agreement. For longitudinal studies, the timeframe of sampling was potentially relevant: of the eight low-agreement longitudinal studies, 7/8 sampled for ≤12 months and only 1/8 for >12 months. Conversely, 14/20 medium/high-agreement longitudinal studies sampled for >12 months, and only 2/20 for <6 months. Studies deployed several wastewater sampling methods, with grab and flow-proportional sampling studies equally distributed across agreement categories (8/12 [67%] and 4/6 [67%] moderate-/high-agreement respectively) but interestingly, composite sampling was more associated with moderate-/high-agreement (8/9; 89%). Of note, sampling point or method was not reported by six and five studies respectively. Most studies performed comparisons on wastewater at least in part derived from the human population sampled (i.e. direct comparisons, 18/34; 53%), while eight conducted indirect comparisons, one conducted both and seven were unclear/unreported. Most moderate-/high-agreement studies conducted direct comparisons (14/21; 67% where reported). Lastly, studies investigating 1–2 WWTWs made up the majority (22/34) but were similarly associated to moderate-/high-agreement (17/22; 77%) as those investigating ≥3 sites (7/10; 70%).

### 3.4 Studies without extractable data

The nine studies without extractable data that could be synthesised are summarised below in terms of their overall ability to detect wastewater-human AMR associations based on reported conclusions. Full descriptive summaries including study details and specific findings are in supplementary data**set 8.**

Two studies performed direct AMR gene detection using qPCR of either 229 (Pärnänen et al., 2019) or eight AMR genes (Colomer-Lluch et al., 2014); both reported a relationship between wastewater AMR and national AMR data.

Four studies employed metagenomics to identify potential wastewater-human AMR associations. Two of these studies appeared to demonstrate an association while the other two were inconclusive.

Two studies used mixed approaches combining phenotypic AST with qPCR (2-targets) (Meir-Gruber et al., 2016) and single isolate WGS (Gouliouris et al., 2019); one study used a phenotypic approach only (YoungKeun et al., 2015). All three studies appeared to show a wastewater-human AMR association.

## 4 Discussion

From our review and synthesis of the available data, we found characterisation of AMR in wastewater shows promise in reflecting AMR in human populations, irrespective of diverse target species, target resistances and study locations, although associations may be stronger for some species and AMR mechanisms than others, and may vary by setting and over time. The strength of this relationship varied across studies and was likely influenced by study features (e.g. design, setting, spatiotemporal sampling strategies) and AMR detection method (i.e. genotypic/phenotypic); the heterogeneity of methodological approaches and lack of clear reporting of key study features made any quantitative synthesis very difficult.

### 4.1 Effect of AMR detection method on wastewater-human AMR concordance

Our estimates of concordance (Fig. 3**A, 3B**) supporting a wastewater-human AMR correlation are in line with estimates in individual studies (Huijbers et al., 2020, Hutinel et al., 2019, Karkman et al., 2020). In particular, Huijbers et al., 2020 reported coefficients of determination as 0.62–0.72 for individual antibiotics and 0.85 when data was combined for four antibiotic classes – similar to our findings of 0.85 and 0.88 for class-unrestricted phenotypic and genotypic data respectively. Although data was too limited to robustly estimate Lin’s CCC for individual species and AMR, variability in the level of discordance of wastewater-human comparisons (Fig. 3**A, 3B – right panels**) and overlap in 95% CI around point estimates (Fig.S7-**23**) is likely attributable in part to specific species and AMR mechanism that are optimally suited to wastewater-based AMR surveillance. This phenomenon was also reported in several studies without extractable prevalence data where specific AMR classes/genes exhibited notably higher/lower wastewater-human concordance.

Genotypic AMR detection methods showed a slight performance increase (non-significant) for both Lin’s CCC and in the reduced proportion of strongly discordant comparisons (Fig. 3**B right panels)** which may reflect their relatively species- and mechanism-agnostic nature (mostly WGS in extracted data) over phenotypic methods which may be more susceptible to variations from differing growth media/conditions and interpretation of resistance breakpoints. Problems with accurately characterising AMR prevalence when only small numbers of isolates are analysed (median of 94–130 for human and 91–98 for wastewater compartments across pheno-/genotypic comparisons here) is also a concern highlighted by previous researchers, particularly when few resistant isolates are available (Huijbers et al., 2020).

Although not a focus in our review, genotypic profiling potentially affords some additional advantages over phenotypic analyses, and is relevant to confirming that genetic mechanisms underpinning phenotypes are also similar. Genomic approaches, such as sequencing of isolates or whole sample metagenomics, enable a more agnostic approach to be adopted than for qPCR, in that analyses do not need to be restricted to a subset of predefined genes/gene variants. Genomic data and profiles can also be more readily shared with the wider community to allow for cross-study comparisons and data synthesis; as demonstrated by Karkman et al. Genomic approaches also allow for the evaluation of genetic relatedness and quantitation of either isolates or microbial populations across compartments (e.g. through phylogenetics, taxonomic/strain-level profiling and strain-based comparisons using metagenomes). Genomic approaches may be higher-resolution and more flexible, but at a higher resource cost; sensitivity for the detection of AMR genes is also dependent on sequencing depth, and accurately associating specific AMR gene markers with strains or species in short-read based metagenomes remains difficult (Gweon et al., 2019).

### 4.2 Effect of study features on wastewater-human AMR concordance

Our review highlights the need for clear guidance on performing and reporting these studies in a more standardised way, with a view to consolidating best-practice approaches in a workflow whilst enabling some flexibility to account for differences in any given setting. Although logistic regression found no significant associations, descriptive synthesis identified study features potentially associated with higher wastewater-human AMR concordance and these are discussed in the context of existing literature here.

WWTW influent is likely the most population-representative wastewater sample for AMR surveillance using either phenotypic or genotypic approaches. This is not unexpected, as previous studies have described transformation of microbial and AMR gene composition during treatment (Tong et al., 2019, Zhang et al., 2020). Transformed samples may remain useful for wastewater-based AMR surveillance, potentially dependent on treatment process; however differing levels of treatment have been shown to select for different species/AMR determinants (Tong et al., 2019). Additionally, a temporospatial overlap of wastewater sampled and the target surveillance population is likely helpful; a feature of the majority of moderate-/high-agreement studies as well as sampling fewer WWTWs which may also relate to the closeness of the populations compared.

Composite sampling also seems sensible as wastewater composition changes significantly over short timescales (Guo et al., 2019) and individual grab samples may be “flooded” by homogenous solid material (Reinthaler et al., 2013). However, grab sampling is convenient and avoids significant autosampler-associated workload and capital costs, and was the most common sampling method used in the studies analysed. Further research is needed into characterising how effectively single timepoint grab samples versus composite/proportional samples reflect temporal AMR changes.

Longitudinal sampling with timeframes over 12 months was most common in both phenotypic and genotypic high agreement studies, consistent with data from two studies which could not be directly synthesised (Hendriksen et al., 2019a, Pignato et al., 2010). In these studies, both used two-weekly sampling intervals over 12- and 3-month timeframes respectively. The former observed an association between ampicillin-resistant wastewater and contemporaneous clinical isolates, whereas the latter found no relationship between contemporaneous public health surveillance and wastewater metagenomic read abundances.

### 4.3 Recommendations regarding risk of bias

Only seven included studies were judged as low risk based on our risk of bias assessment (**Table S2**); however a sensitivity analysis of wastewater-human AMR prevalence concordance in these data (Fig.S6) showed similar results to the main analysis of all studies. Although high risk of bias does not necessarily mean that the data cannot be considered in a data synthesis, and our concordance sensitivity analysis appears to show this for included studies, minimising the risk of bias is key to producing robust data for future evaluations of wastewater-based AMR surveillance.

Several potential biases may be challenging to manage in the context of wastewater sampling, for example those that arise from logistical reasons concerning inaccessibility of sampling sites or sampling equipment. While unexpected wastewater site closures cannot be anticipated (Pärnänen et al., 2019) and achieving exactly the same sampling methods across highly variable sampling sites is challenging in large-scale collaborations (Hendriksen et al., 2019b), future studies should attempt to minimise any differences across wastewater sampling sites where feasible. For example, Huijbers et al. consistently sampled wastewater sites during the mid-week to avoid potential weekend effects known to affect chemical wastewater-based epidemiology, and Urase et al. purposefully avoided sampling combined sewer wastewater sites during rain events to avoid potential dilution and flow variation. Additional metadata such as sample storage conditions or freeze/thaw cycles are also pertinent to interpretation as investigated by Poulsen et al., who similarly suggested detailed reporting of these features.

Future studies should clearly report sewer inputs, including any unique AMR-associated inputs (e.g. hospitals, agricultural sources) that may confound AMR prevalence estimates (Fahrenfeld and Bisceglia, 2016). The importance of specific AMR-associated inputs is likely linked to whether the AMR mechanisms under evaluation are uniquely associated with that specific source, or already widely disseminated in the community. For example, one study (Raven et al., 2019) sampled WWTWs with and without hospital input, and found the most clinically-prevalent *E. coli* ESBL gene was ubiquitous in all WWTWs, indicating prior widespread dissemination in the community. Another study (Jakobsen et al., 2008) focussing on *E. coli* gentamicin resistance in hospital effluent, receiving WWTW influent and domestic-only wastewater, found significantly lower prevalence in domestic-only wastewater compared to hospital effluent and WWTW influent which shared similar prevalence, indicating that the presence of any hospital-associated wastewater in influent was not representative of community-based estimates.

### 4.4 Limitations

Our study has several limitations. Although we conducted a comprehensive risk of bias assessment, the study pool was too small to feasibly conduct risk-stratified synthesis and meta-regression without substantial loss of power (Higgins and Thompson, 2004). To mitigate this, a modified version of the GRADE system was used to incorporate summary assessments of the quality of the evidence into the interpretation of results (Higgins et al., 2011). Certainty of the evidence base was rated as “low to moderate” which potentially indicates reduced confidence in our conclusions/recommendations, however, our certainty assessment is a conservative estimate omitting two upgrade domains, and true certainty may be higher if data was available to assess these domains. Since most human datasets available in the included studies were clinical in origin, human compartment AMR prevalence estimates were potentially susceptible to biases outlined in our introduction (i.e. overestimation of the “true” population-level AMR burden). However, as seen in existing literature and in the results of this review, clinical and wastewater AMR prevalence estimates do appear to mirror each other. We excluded non-English publications, potentially missing some relevant studies. Studies were highly diverse in reported features, design and outcomes, making a comprehensive synthesis difficult. In particular, many features were poorly characterised and could not be explored in our analyses. For our study feature analysis we focused *a priori* on features that optimised the identification of an association between wastewater and human AMR prevalence, however it may be that in some circumstances there is genuinely no such association. In many settings globally, established wastewater infrastructures are not available, and an analysis of, for example, WWTW influent may not be feasible; open sewerage systems may represent an alternative sampling point in these contexts.

## 5 Conclusion

In conclusion our review suggests that overall, wastewater-based surveillance has significant potential for monitoring population-level AMR, particularly for some species, despite high diversity in study design, methods and metadata. We found that no specific study feature or AMR detection method conferred a clear increase in the ability of a study to detect an association between wastewater and human AMR prevalence. However, based on limited available data, we would recommend that where feasible, genotypic AMR detection, composite sampling of influent with longitudinal timeframe >12 months, and contemporaneous sampling of wastewater and human compartments that are directly associated (i.e. the human population sampled contributes to the wastewater sampled) are used to generate more robust data to better evaluate the strengths and limitations of this approach for surveillance purposes. Clear reporting of study methods and features are essential, and this will facilitate the iterative development of optimal practice guidelines for this emerging surveillance tool.

## 6 Contributors

A complete list of author contributions as per CRediT; Contributor Roles Taxonomy is given in the appendix (p23).

## Funding

This work was supported by the Medical Research Foundation National PhD Training Programme in Antimicrobial Resistance Research, the National Institute for Health Research (NIHR) Health Protection Research Unit in Healthcare Associated Infections and Antimicrobial Resistance at University of Oxford (NIHR200915) in partnership with the UK Health Security Agency (UKHSA), and the NIHR Oxford Biomedical Research Centre. The report presents independent research. The views expressed in this publication are those of the authors and not necessarily those of the NHS, NIHR, UKHSA or the Department of Health and Social Care.

KKC is a Medical Research Foundation PhD student (ref. MRF-145-0004-TPG-AVISO). NS is an Oxford Martin School fellow and an NIHR Oxford BRC Senior Research Fellow. ASW and DWC are NIHR Senior Investigators.

## Declaration of Competing Interest

The authors declare that they have no known competing financial interests or personal relationships that could have appeared to influence the work reported in this paper.

## References

[b0005] Aarestrup F.M., Woolhouse M.E.J. (2020). Using sewage for surveillance of antimicrobial resistance. Science (80-.).

[b0010] Adator E.H., Narvaez-Bravo C., Zaheer R., Cook S.R., Tymensen L., Hannon S.J., Booker C.W., Church D., Read R.R., McAllister T.A. (2020). A One Health Comparative Assessment of Antimicrobial Resistance in Generic and Extended-Spectrum Cephalosporin-Resistant Escherichia coli from Beef Production, Sewage and Clinical Settings. Microorganisms.

[b0015] Adator E.H., Walker M., Narvaez-Bravo C., Zaheer R., Goji N., Cook S.R., Tymensen L., Hannon S.J., Church D., Booker C.W., Amoako K., Nadon C.A., Read R., McAllister T.A. (2020). Whole Genome Sequencing Differentiates Presumptive Extended Spectrum Beta-Lactamase Producing Escherichia coli along Segments of the One Health Continuum. Microorganisms.

[b0020] AGISAR, 2018. Critically Important Antimicrobials for Human Medicine 6th Revision 2018. Ranking of medically important antimicrobials for risk management of antimicrobial resistance due to non-human use. https://Apps.Who.Int/Iris/Bitstream/Handle/10665/312266/9789241515528-Eng.Pdf?Ua=1.

[b0025] Ahmed W., Angel N., Edson J., Bibby K., Bivins A., O’Brien J.W., Choi P.M., Kitajima M., Simpson S.L., Li J., Tscharke B., Verhagen R., Smith W.J.M., Zaugg J., Dierens L., Hugenholtz P., Thomas K.V., Mueller J.F. (2020). First confirmed detection of SARS-CoV-2 in untreated wastewater in Australia: a proof of concept for the wastewater surveillance of COVID-19 in the community. Sci. Total Environ..

[b0030] Ahmed W., Bivins A., Bertsch P., Bibby K., Choi P., Farkas K., Gyawali P., Hamilton K., Haramoto E., Kitajima M., Simpson S., Tandukar S., Thomas K., Mueller J. (2020). Surveillance of SARS-CoV-2 RNA in wastewater: Methods optimization and quality control are crucial for generating reliable public health information. Curr. Opin. Environ. Sci. Health.

[b0035] Aljanaby A.A.J. (2018). Antibiotics susceptibility pattern and virulence-associated genes in clinical and environment strains of Pseudomonas aeruginosa in Iraq. Asian J. Sci. Res..

[b0040] Asghar H., Diop O.M., Weldegebriel G., Malik F., Shetty S., Bassioni L.E., Akande A.O., Maamoun E.A., Zaidi S., Adeniji A.J., Burns C.C., Deshpande J., Oberste M.S., Lowther S.A. (2014). Environmental surveillance for polioviruses in the global polio eradication initiative. J. Infect. Dis..

[bib356] Choi P., Tscharke B., Donner E., O’Brien J., Grant S., Kaserzon S., Mackie R., O’Malley E., Crosbie N., Thomas K., Mueller J. (2018). Wastewater-based epidemiology biomarkers: Past, present and future. Trends Anal. Chem..

[b0045] Colomer-Lluch M., Calero-Cáceres W., Jebri S., Hmaied F., Muniesa M., Jofre J. (2014). Antibiotic resistance genes in bacterial and bacteriophage fractions of Tunisian and Spanish wastewaters as markers to compare the antibiotic resistance patterns in each population. Environ. Int..

[b0050] Davies T.J. (2019).

[b0055] Davies T., Stoesser N., Sheppard A., Abuoun M., Fowler P., Swann J., Quan T., Griffiths D., Vaughan A., Morgan M., Phan H., Jeffery K., Andersson M., Ellington M., Ekelund O., Woodford N., Mathers A., Bonomo R., Crook D., Peto T., Anjum M., Walker A. (2020). Reconciling the Potentially Irreconcilable? Genotypic and Phenotypic Amoxicillin-Clavulanate Resistance in Escherichia coli. Antimicrob. Agents Chemother..

[b0060] CAB Abstracts, 2021. CABI. https://www.cabi.org/publishing-products/cab-abstracts.

[b0065] EMBASE, 2021. Elsevier. https://www.embase.com.

[b0070] Fahrenfeld N., Bisceglia K.J. (2016). Emerging investigators series: Sewer surveillance for monitoring antibiotic use and prevalence of antibiotic resistance: Urban sewer epidemiology. Environ. Sci. Water Res. Technol..

[b0075] Fernández M.D.B., Torres C., Poma H.R., Riviello-López G., Martínez L.C., Cisterna D.M., Rajal V.B., Nates S.V., Mbayed V.A. (2012). Environmental surveillance of norovirus in Argentina revealed distinct viral diversity patterns, seasonality and spatio-temporal diffusion processes. Sci. Total Environ..

[b0080] Global Health, 2021. EBSCO Information Services. https://www.ebsco.com/products/research-databases/global-health.

[b0085] González-Mariño, I., J. A. Baz-Lomba, N. A. Alygizakis, M. J. Andrés-Costa, R. Bade, A. Bannwarth, L., P. Barron, F. Been, L. Benaglia, J. D. Berset, L. Bijlsma, I. Bodík, A. Brenner, A. L. Brock, D. A. Burgard, E. Castrignanò, A. Celma, C. E. Christophoridis, A. Covaci, O. Delémont, P. de Voogt, D. A. Devault, M. J. Dias, E. Emke, P. Esseiva, D. Fatta-Kassinos, G. Fedorova, K. Fytianos, C. Gerber, R. Grabic, E. Gracia-Lor, S. Grüner, T. Gunnar, E. Hapeshi, E. Heath, B. Helm, F. Hernández, A. Kankaanpaa, S. Karolak, B. Kasprzyk-Hordern, I. Krizman-Matasic, F. Y. Lai, W. Lechowicz, A. Lopes, M. López de Alda, E. López-García, A. S. C. Löve, N. Mastroianni, G. L. McEneff, R. Montes, K. Munro, T. Nefau, H. Oberacher, J. W. O'Brien, R. Oertel, K. Olafsdottir, Y. Picó, B. G. Plósz, F. Polesel, C. Postigo, J. B. Quintana, P. Ramin, M. J. Reid, J. Rice, R. Rodil, N. Salgueiro-González, S. Schubert, I. Senta, S. M. Simões, M. M. Sremacki, K. Styszko, S. Terzic, N. S. Thomaidis, K. V. Thomas, B. J. Tscharke, R. Udrisard, A. L. N. van Nuijs, V. Yargeau, E. Zuccato, S. Castiglioni and C. Ort (2020). Spatio-temporal assessment of illicit drug use at large scale: evidence from 7 years of international wastewater monitoring. Addiction 115(1), 109-120. 10.1111/add.14767. PMC6973045 31642141

[b0090] Gouliouris T., Raven K.E., Moradigaravand D., Ludden C., Coll F., Blane B., Naydenova P., Horner C., Brown N.M., Corander J., Limmathurotsakul D., Parkhill J., Peacock S.J. (2019). Detection of vancomycin-resistant Enterococcus faecium hospital-adapted lineages in municipal wastewater treatment plants indicates widespread distribution and release into the environment. Genome Res..

[b0095] Guo B., Liu C., Gibson C., Frigon D. (2019). Wastewater microbial community structure and functional traits change over short timescales. Sci. Total Environ..

[b0100] Gweon, H.S., Shaw, L.P., Swann, J., De Maio, N., Abuoun, M., Niehus, R., Hubbard, A.T.M., Bowes, M.J., Bailey, M.J., Peto, T.E.A., Hoosdally, S.J., Walker, A.S., Sebra, R.P., Crook, D.W., Anjum, M.F., Read, D.S., Stoesser, N., 2019. The impact of sequencing depth on the inferred taxonomic composition and AMR gene content of metagenomic samples. Environ. Microbiomes. 10.1186/s40793-019-0347-1. PMC8204541 33902704

[b0105] Haghi, F., Shirmohammadlou, N., Bagheri, R., Jamali, S., Zeighami, H., 2019. High frequency of vancomycin-resistant enterococci in sewage and fecal samples of healthy carriers. Open Biotechnol. J. 13, 1–5. 10.2174/1874070701913010001.

[b0110] Hay S.I., Rao P.C., Dolecek C., Day N.P.J., Stergachis A., Lopez A.D., Murray C.J.L. (2018). Measuring and mapping the global burden of antimicrobial resistance. BMC Med..

[b0115] Hendriksen, R.S., Lukjancenko, O., Munk, P., Hjelmsø, M.H., Verani, J.R., Ng’eno, E., Bigogo, G., Kiplangat, S., Oumar, T., Bergmark, L., Röder, T., Neatherlin, J.C., Clayton, O., Hald, T., Karlsmose, S., Pamp, S.J., Fields, B., Montgomery, J.M., Aarestrup, F.M., 2019a. Pathogen surveillance in the informal settlement, Kibera, Kenya, using a metagenomics approach. PLoS One 14, e0222531. 10.1371/journal.pone.0222531. PMC6786639 31600207

[b0120] Hendriksen, R.S., Munk, P., Njage, P., van Bunnik, B., McNally, L., Lukjancenko, O., Roder, T., Nieuwenhuijse, D., Pedersen, S.K., Kjeldgaard, J., Kaas, R.S., Clausen, P.T.L.C., Vogt, J.K., Leekitcharoenphon, P., van de Schans, M.G.M., Zuidema, T., de Roda Husman, A.M., Rasmussen, S., Petersen, B., consortium, G.S.S. project, Amid, C., Cochrane, G., Sicheritz-Ponten, T., Schmitt, H., Alvarez, J.R.M., Aidara-Kane, A., Pamp, S.J., Lund, O., Hald, T., Woolhouse, M., Koopmans, M.P., Vigre, H., Petersen, T.N., Aarestrup, F.M., 2019b. Global monitoring of antimicrobial resistance based on metagenomics analyses of urban sewage. Nat. Commun. 10, 1124. 10.1038/s41467-019-08853-3. PMC6408512 30850636

[b0125] Higgins J.P.T., Altman D.G., Gøtzsche P.C., Jüni P., Moher D., Oxman A.D., Savović J., Schulz K.F., Weeks L., Sterne J.A.C. (2011). The Cochrane Collaboration’s tool for assessing risk of bias in randomised trials. BMJ.

[b0130] Higgins J., Thompson S. (2004). Controlling the risk of spurious findings from meta-regression. Stat. Med..

[b0135] Honda R., Tachi C., Yasuda K., Hirata T., Noguchi M., Hara-Yamamura H., Yamamoto-Ikemoto R., Watanabe T. (2020). Estimated discharge of antibiotic-resistant bacteria from combined sewer overflows of urban sewage system. npj Clean. Water.

[b0140] Huijbers P.M.C., Larsson D.G.J., Flach C.F. (2020). Surveillance of antibiotic resistant Escherichia coli in human populations through urban wastewater in ten European countries. Environ. Pollut..

[b0145] Hutinel M., Huijbers P.M.C., Fick J., Ahren C., Larsson D.G.J., Flach C.-F.-F. (2019). Population-level surveillance of antibiotic resistance in Escherichia coli through sewage analysis. Euro. Surveill..

[b0150] Jakobsen L., Sandvang D., Hansen L.H., Bagger-Skjøt L., Westh H., Jørgensen C., Hansen D.S., Pedersen B.M., Monnet D.L., Frimodt-Møller N., Sørensen S.J., Hammerum A.M. (2008). Characterisation, dissemination and persistence of gentamicin resistant Escherichia coli from a Danish university hospital to the wastewater environment. Environ. Int..

[b0155] Jørgensen S.B., Søraas A.V., Arnesen L.S., Leegaard T.M., Sundsfjord A., Jenum P.A. (2017). A comparison of extended spectrum beta-lactamase producing Escherichia coli from clinical, recreational water and wastewater samples associated in time and location. PLoS ONE.

[b0160] Karkman A., Berglund F., Flach C.-F., Kristiansson E., Larsson D.G.J. (2020). Predicting clinical resistance prevalence using sewage metagenomic data. Commun. Biol..

[b0165] King T., Schmidt S., Essack S. (2020). Antibiotic resistant Klebsiella spp. from a hospital, hospital effluents and wastewater treatment plants in the uMgungundlovu District, KwaZulu-Natal, South Africa. Sci. Total Environ..

[b0170] Knudsen, B., Bergmark, L., Munk, P., Lukjancenko, O., Priemé, A., Aarestrup, F. and Pamp, S., 2016. Impact of Sample Type and DNA Isolation Procedure on Genomic Inference of Microbiome Composition. mSystems, 1(5). 10.1128/mSystems.00095-16. PMC5080404 27822556

[b0175] Lal Gupta C., Kumar Tiwari R., Cytryn E. (2020). Platforms for elucidating antibiotic resistance in single genomes and complex metagenomes. Environ. Int..

[b0180] Larson R., Berman O., Nourinejad M. (2020). Sampling Manholes to Home in on SARS-CoV-2 Infections. SSRN Electron. J..

[b0185] MEDLINE, 2021. National Library of Medicine - National Institutes of Health. https://www.nlm.nih.gov/.

[b0190] Meir-Gruber, L., Manor, Y., Gefen-Halevi, S., Hindiyeh, M.Y., Mileguir, F., Azar, R., Smollan, G., Belausov, N., Rahav, G., Shamiss, A., Mendelson, E., Keller, N., 2016. Population Screening Using Sewage Reveals Pan-Resistant Bacteria in Hospital and Community Samples. PLoS One, [Erratum in: PLoS One. 2017 Jan 12;12 (1):e0170538; PMID: 28081232 [https://www.ncbi.nlm.nih.gov/pubmed/28081232]] 11, e0164873. 10.1371/journal.pone.0164873. PMC5079554 27780222

[b0195] Michael-Kordatou I., Karaolia P., Fatta-Kassinos D. (2020). Sewage analysis as a tool for the COVID-19 pandemic response and management: the urgent need for optimised protocols for SARS-CoV-2 detection and quantification. J. Environ. Chem. Eng..

[b0200] Moradigaravand, D., Gouliouris, T., Ludden, C., Reuter, S., Jamrozy, D., Blane, B., Naydenova, P., Judge, K., H Aliyu, S., F Hadjirin, N., A Holmes, M., Török, E., M Brown, N., Parkhill, J., Peacock, S., 2018. Genomic survey of Clostridium difficile reservoirs in the East of England implicates environmental contamination of wastewater treatment plants by clinical lineages. Microb. genomics 4. 10.1099/mgen.0.000162. PMC5885014 29498619

[b0205] Morgan R.L., Thayer K.A., Bero L., Bruce N., Falck-Ytter Y., Ghersi D., Guyatt G., Hooijmans C., Langendam M., Mandrioli D., Mustafa R.A., Rehfuess E.A., Rooney A.A., Shea B., Silbergeld E.K., Sutton P., Wolfe M.S., Woodruff T.J., Verbeek J.H., Holloway A.C., Santesso N., Schünemann H.J. (2016). GRADE: Assessing the quality of evidence in environmental and occupational health. Environ. Int..

[bib357] Mourkas E., Florez-Cuadrado D., Pascoe B., Calland J., Bayliss S., Mageiros L., Méric G., Hitchings M., Quesada A., Porrero C., Ugarte-Ruiz M., Gutiérrez-Fernández J., Domínguez L., Sheppard S. (2019). Gene pool transmission of multidrug resistance among *Campylobacter* from livestock, sewage and human disease. Environ. Microbiol..

[b0210] Newton R.J., McLellan S.L., Dila D.K., Vineis J.H., Morrison H.G., Murat Eren A., Sogin M.L. (2015). Sewage reflects the microbiomes of human populations. MBio.

[b0215] O’Neill, J., 2016. Tackling Drug-Resistant Infections Globally: Final Report and Recommendations. https://amr-review.org/sites/default/files/160525_Final%20paper_with%20cover.pdf.

[b0220] Ojer-Usoz, E., González, D., Vitas, A.I., 2017. Clonal diversity of ESBL-producing Escherichia coli isolated from environmental, human and food samples. Int. J. Environ. Res. Public Health 14, 676. 10.3390/ijerph14070676. PMC5551114 28644413

[b0225] Oravcova V., Mihalcin M., Zakova J., Pospisilova L., Masarikova M., Literak I. (2017). Vancomycin-resistant enterococci with vanA gene in treated municipal wastewater and their association with human hospital strains. Sci. Total Environ..

[b0230] Pärnänen K.M.M., Narciso-Da-Rocha C., Kneis D., Berendonk T.U., Cacace D., Do T.T., Elpers C., Fatta-Kassinos D., Henriques I., Jaeger T., Karkman A., Martinez J.L., Michael S.G., Michael-Kordatou I., O’Sullivan K., Rodriguez-Mozaz S., Schwartz T., Sheng H., Sørum H., Stedtfeld R.D., Tiedje J.M., Giustina S.V.D., Walsh F., Vaz-Moreira I., Virta M., Manaia C.M. (2019). Antibiotic resistance in European wastewater treatment plants mirrors the pattern of clinical antibiotic resistance prevalence. Sci. Adv..

[b0235] Pehrsson E.C., Tsukayama P., Patel S., Mejia-Bautista M., Sosa-Soto G., Navarrete K.M., Calderon M., Cabrera L., Hoyos-Arango W., Bertoli M.T., Berg D.E., Gilman R.H., Dantas G. (2016). Interconnected microbiomes and resistomes in low-income human habitats. Nature.

[b0240] Pignato S., Coniglio M.A., Faro G., Lefevre M., Weill F.X., Giammanco G. (2010). Molecular epidemiology of ampicillin resistance in Salmonella spp. and Escherichia coli from wastewater and clinical specimens. Foodborne Pathog. Dis..

[b0245] Pot, M., Guyomard-Rabenirina, S., Couvin, D., Ducat, C., Enouf, V., Ferdinand, S., Gruel, G., Malpote, E., Talarmin, A., Breurec, S., Reynaud, Y., 2020. Dissemination of ESBL-producing Enterobacter cloacae complex from a hospital to the nearby environment in Guadeloupe (French West Indies): ST114 lineage coding for successful IncHI2/ST1 plasmid. Antimicrob. Agents Chemother. 10.1128/AAC.02146-20. PMC8092524 33361294

[b0250] Poulsen, C.S., Kaas, R.S., Aarestrup, F.M., Pamp, S.J., 2021. Standard Sample Storage Conditions Impact on Inferred Microbiome Composition and Antimicrobial Resistance Patterns. bioRxiv. 2021.05.24.445395. 10.1101/2021.05.24.445395. PMC8510183 34612701

[b0255] Rahimi F., Bouzari M. (2015). Biochemical fingerprinting of methicillin-resistant staphylococcus aureus isolated from sewage and hospital in Iran. Jundishapur J. Microbiol..

[b0260] Raven K.E., Ludden C., Gouliouris T., Blane B., Naydenova P., Brown N.M., Parkhill J., Peacock S.J. (2019). Genomic surveillance of Escherichia coli in municipal wastewater treatment plants as an indicator of clinically relevant pathogens and their resistance genes. Microb. Genomics.

[b0265] Reinthaler F.F., Galler H., Feierl G., Haas D., Leitner E., Mascher F., Melkes A., Posch J., Pertschy B., Winter I., Himmel W., Marth E., Zarfel G. (2013). Resistance patterns of Escherichia coli isolated from sewage sludge in comparison with those isolated from human patients in 2000 and 2009. J. Water Health.

[b0270] Saifi M., Pourshafie M.R., Dallal M.M.S., Katouli M. (2009). Clonal groups of high-level gentamicin-resistant Enterococcus faecium isolated from municipal wastewater and clinical samples in Tehran. Iran. Lett. Appl. Microbiol..

[b0275] SCOPUS, 2021. Elsevier. https://www.scopus.com.

[b0280] Shanks O., Newton R., Kelty C., Huse S., Sogin M., McLellan S. (2013). Comparison of the Microbial Community Structures of Untreated Wastewaters from Different Geographic Locales. Appl. Environ. Microbiol..

[b0285] Shaw L., Chau K., Kavanagh J., AbuOun M., Stubberfield E., Gweon H., Barker L., Rodger G., Bowes M., Hubbard A., Pickford H., Swann J., Gilson D., Smith R., Hoosdally S., Sebra R., Brett H., Peto T., Bailey M., Crook D., Read D., Anjum M., Walker A., Stoesser N. (2021). Niche and local geography shape the pangenome of wastewater- and livestock-associated Enterobacteriaceae. Sci. Adv..

[b0290] Shchegolkova N., Krasnov G., Belova A., Dmitriev A., Kharitonov S., Klimina K., Melnikova N., Kudryavtseva A. (2016). Microbial Community Structure of Activated Sludge in Treatment Plants with Different Wastewater Compositions. Front. Microbiol..

[b0295] Su, J.-Q., An, X.-L., Li, B., Chen, Q.-L., Gillings, M.R., Chen, H., Zhang, T., Zhu, Y.-G., 2017. Metagenomics of urban sewage identifies an extensively shared antibiotic resistome in China. Microbiome, [Erratum in: Microbiome. 2018 Jul 9;6(1):127; PMID: 29986764 [https://www.ncbi.nlm.nih.gov/pubmed/29986764]] 5, 84. 10.1186/s40168-017-0298-y. PMC5517792 28724443

[b0300] Tacconelli E., Sifakis F., Harbarth S., Schrijver R., van Mourik M., Voss A., Sharland M., Rajendran N.B., Rodríguez-Baño J., Bielicki J., de Kraker M., Gandra S., Gastmeier P., Gilchrist K., Gikas A., Gladstone B.P., Goossens H., Jafri H., Kahlmeter G., Leus F., Luxemburger C., Malhotra-Kumar S., Marasca G., McCarthy M., Navarro M.D., Nuñez-Nuñez M., Oualim A., Price J., Robert J., Sommer H., von Cube M., Vuong C., Wiegand I., Witschi A.T., Wolkewitz M. (2018). Surveillance for control of antimicrobial resistance. Lancet Infect. Dis..

[b0305] Tong J., Tang A., Wang H., Liu X., Huang Z., Wang Z., Zhang J., Wei Y., Su Y., Zhang Y. (2019). Microbial community evolution and fate of antibiotic resistance genes along six different full-scale municipal wastewater treatment processes. Bioresour. Technol..

[b0310] Urase T., Okazaki M., Tsutsui H. (2020). Prevalence of ESBL-producing Escherichia coli and carbapenem-resistant Enterobacteriaceae in treated wastewater: a comparison with nosocomial infection surveillance. J. Water Health.

[b0315] Web of Science Core Collection, 2021. Web of Science Group - Clarivate. https://clarivate.com/webofsciencegroup/solutions/web-of-science-core-collection.

[b0320] WHO, 2019. Global action plan on AMR: Objective 2. https://apps.who.int/iris/bitstream/handle/10665/193736/9789241509763_eng.pdf?sequence=1.

[b0325] WHO, 2018. Global Antimicrobial Resistance Surveillance System (GLASS) Report Early implementation 2017-18. https://apps.who.int/iris/bitstream/handle/10665/279656/9789241515061-eng.pdf?ua=1.

[b0330] Woodruff, T. and Sutton, P., 2011. An Evidence-Based Medicine Methodology To Bridge The Gap Between Clinical And Environmental Health Sciences. Health Affairs, 30(5), pp.931-937. 10.1377/hlthaff.2010.1219. PMC6663095 21555477

[b0335] Wright, G.D., 2007. The antibiotic resistome: The nexus of chemical and genetic diversity. Nat. Rev. Microbiol. 5, 175–186. 10.1038/nrmicro1614. 17277795

[b0340] YoungKeun K., Colque P., Byfors S., Giske C.G., Mollby R., Kuhn I. (2015). Surveillance of antimicrobial resistance among Escherichia coli in wastewater in Stockholm during 1 year: does it reflect the resistance trends in the society?. Int. J. Antimicrob. Agents.

[b0345] Zaheer R., Cook S.R., Barbieri R., Goji N., Cameron A., Petkau A., Polo R.O., Tymensen L., Stamm C., JiMing S., Hannon S., Jones T., Church D., Booker C.W., Amoako K., van Domselaar G., Read R.R., McAllister T.A. (2020). Surveillance of Enterococcus spp. reveals distinct species and antimicrobial resistance diversity across a One-Health continuum. Sci. Rep..

[b0350] Zarfel G., Feierl G., Galler H., Haas D., Leitner E., Mascher F., Melkes A., Posch J., Winter I., Masoud L., Grisold A.J., Marth E. (2010). Comparison of ESBL genes from extended-spectrum beta-lactamase carrying Escherichia coli from sewage sludge and human urinary tract infection. Clin. Microbiol. Infect., 20th ECCMID. Vienna Austria..

[b0355] Zhang L., Cheng Y., Qian C., Lu W. (2020). Bacterial community evolution along full-scale municipal wastewater treatment processes. J. Water Health.

