# Supplementary material for: Systematic review of wastewater surveillance of antimicrobial resistance in human populations
Source: Environ Int. 2022 Apr;162:107171. doi: 10.1016/j.envint.2022.107171 (PMC8960996; doi:10.1016/j.envint.2022.107171)
Supplement: Supplementary data 1 [file mmc1.docx]

**SUPPORTING MATERIAL**

**Figure S1: Detailed screening flowchart**

**
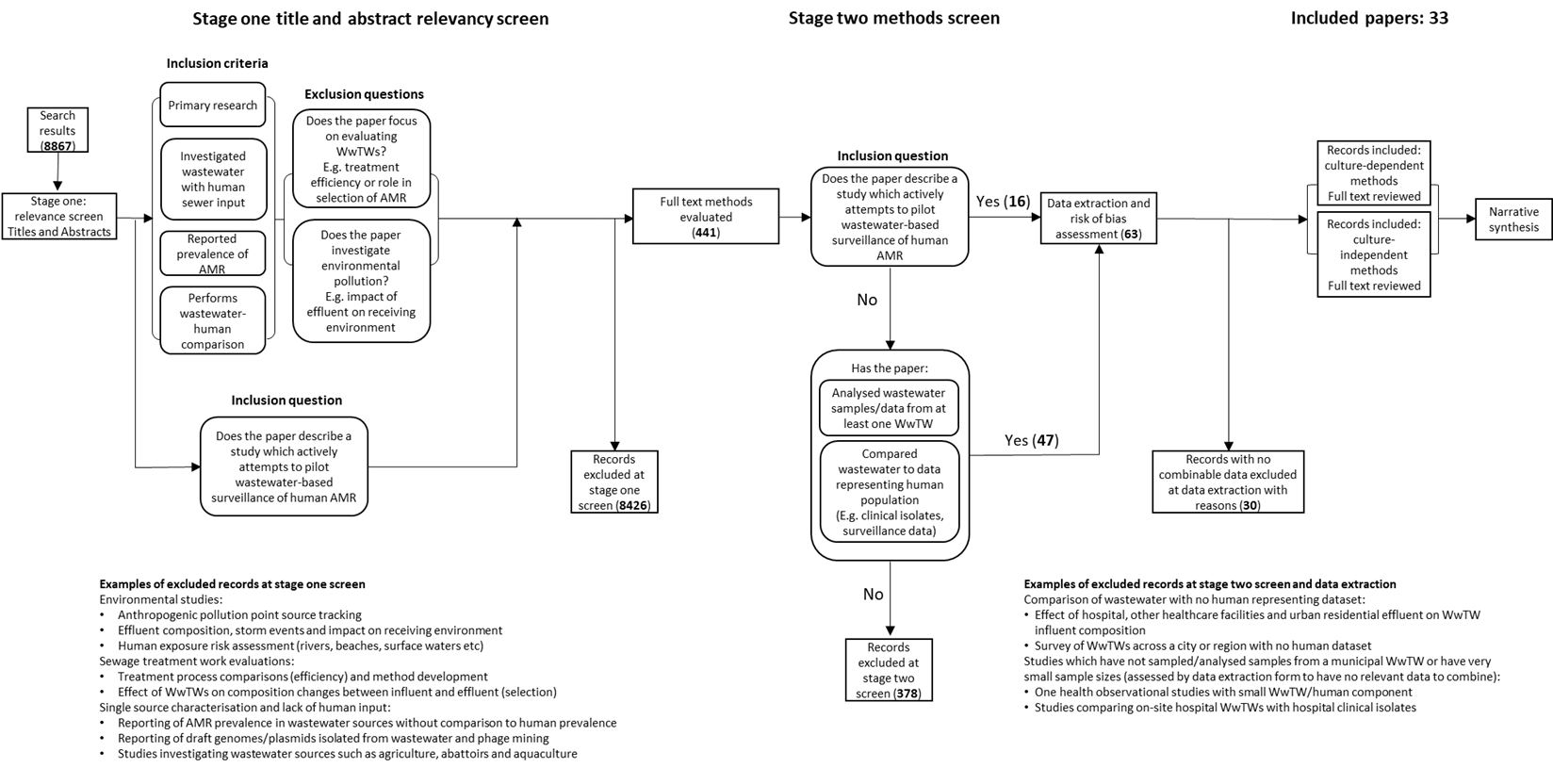
**Screening criteria described for all stages with numbers of passing/excluded records. Examples of excluded records are described at each stage.

**Table S2: Modifications of Cochrane risk of bias tool used for risk of bias assessment**

| **Bias domain** | **Cochrane tool source of bias** | **Cochrane tool bias description** | **Modified tool source of bias** | **Modified tool bias description** |
| --- | --- | --- | --- | --- |
| *Selection bias* | Random sequence generation | Selection bias (biased allocation to interventions) due to inadequate generation of a randomised sequence | Selective sampling of wastewater sites | Selection bias introduced by use of significantly different wastewater sampling method/strategy for specific sampling locations (e.g. WWTWs receiving hospital waste vs domestic waste only) or points (e.g. influent vs effluent) |
|  | Allocation concealment | Selection bias (biased allocation to interventions) due to inadequate concealment of allocations before assignment |  |  |
| *Performance bias* | Blinding of participants and personnel | Performance bias due to knowledge of the allocated interventions by participants and personnel during the study | Use of non-uniform performing methods during outcome assessment | Performance bias introduced between and within sample groups by the use of different processing/AMR detection approaches |
| *Attrition bias* | Incomplete outcome data | Attrition bias due to amount, nature, or handling of incomplete outcome data | Incomplete outcome data | Attrition bias introduced by incomplete outcome data where data fields may be missing across comparison groups (e.g. longitudinal studies in particular may be missing timepoints due to logistical reasons) |
| *Reporting bias* | Selective reporting | Reporting bias due to selective outcome reporting | Selective reporting | Reporting bias due to selective outcome reporting where outcomes may be measured but not reported or disproportionately reported compared to other outcomes |
| *Other bias* | Anything else, ideally prespecified | Bias due to problems not covered elsewhere | Uncertainty of sewer inputs | Confounding bias referring to the uncertain presence of AMR/AMR-influencing sources which could have impacted study outcomes |
| *Detection bias* | Blinding of outcome assessment | Detection bias due to knowledge of the allocated interventions by outcome assessment | Not applicable as knowledge of allocated groups is essential for specific sample processing | NA |

**Table S3: Adapted GRADE domains used for certainty assessment**

|  | **GRADE domain** | **Summary of criteria** | **Example for further clarification where required** |
| --- | --- | --- | --- |
| Domains considered for downgrading | Risk of bias | Summary assessment of study limitations consisting of attrition-, performance-, reporting-, selection- and other-bias. | Examples explained in the main text |
|  | Inconsistency | High variability in the direction of effect. | The body of evidence contains studies reporting both positive and negative correlation between wastewater and human AMR prevalence |
|  | Indirectness | Evidence is not directly related to the systematic review question. | Studies not specifically set up to pilot population-level surveillance using wastewater |
|  | Imprecision | Relating to the overall number of isolates tested in a body of evidence. |  |
|  | Publication bias | Incomplete body of evidence resulting from inadequate search strategy. |  |
| Domains considered for upgrading | Dose response (adapted for this study to “concordance”) | Effect size mirrored across compartments | AMR prevalence estimates in the human compartment is associated with AMR prevalence in the wastewater compartment (i.e. High AMR prevalence estimate in wastewater concords with high AMR prevalence estimate in human compartment). |
|  | Large effect | Not applicable to our systematic review |  |
|  | Confounding minimizes effect | Not applicable to our systematic review |  |

**Table S4: Certainty assessment results and rationale for upgrades and downgrades**

| **Identification of association between AMR prevalence in humans and wastewater** | | | | | | | | | |
| --- | --- | --- | --- | --- | --- | --- | --- | --- | --- |
| **Initial confidence (no. of records)** | **Domains decreasing confidence**  **“---” if not present; “↓” if enough evidence to downgrade** | | | | | **Domains increasing confidence**  **“---” if not present; “↑” if enough evidence to upgrade** | | | **Final confidence rating** |
|  | Risk of Bias | Inconsistency | Indirectness | Imprecision | Publication bias | ”Concordance” | Large effect | Residual confounding |  |
| *Surveillance studies* | | | | | | | | | |
| Initial moderate  (12 records) | ↓ | --- | --- | --- | --- | ↑ | NA | NA | Moderate |
| *Relevant comparison studies* | | | | | | | | | |
| Initial moderate  (21 records) | ↓ | --- | ↓ | --- | --- | ↑ | NA | NA | Low |
| *Rationale* | | | | | | | | | |
|  | Majority of records judged to have overall high risk of bias across both bodies of evidence (8/12, 11/21). | Judging magnitude of effect consistency is not feasible due to heterogenous outcomes but direction of effects is mostly consistent (i.e. a wastewater-human association is observed). | Research evidence for surveillance studies is not dissimilar to our review questions but majority relevant comparison studies aim to answer dissimilar questions. | High total number of participants (i.e. isolates tested) across all studies in both bodies of evidence. | It was not feasible to assess publication bias via funnel plot in our bodies of evidence, but bias is less likely when a comprehensive search strategy like ours is used. | Majority of records conducting statistical testing show a statistically significant association between human and wastewater AMR prevalence across both bodies of evidence. | Not applicable to our bodies of evidence or review question so not assessed. | Not applicable to our bodies of evidence or review question so not assessed. |  |

**Figure S5: Global density of wastewater sampling of included studies**

Count of studies sampling countries plotted by points on country centroids with size reflecting number of studies.


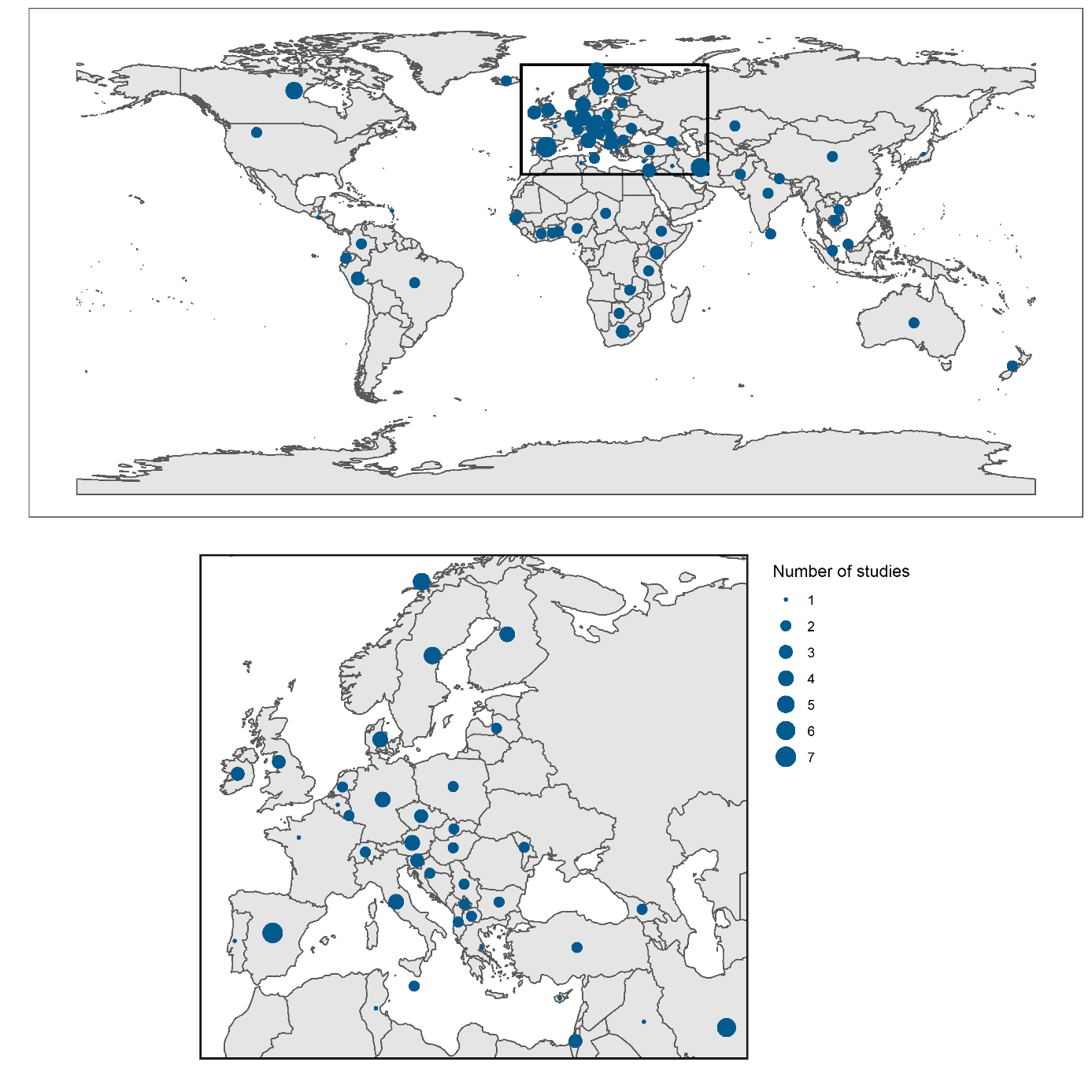


**Figure S6 AMR prevalence in wastewater and human isolates from low bias studies only**

Concordance plot of AMR prevalence in wastewater and human isolates utilising data from low bias studies only. Each point represents a single wastewater-human comparison conducted where colour corresponds to bacterial species tested and shape corresponds to human sample type used. Lin’s concordance correlation coefficient (CCC) is labelled with 95% confidence intervals. Unbroken line of *y*=*x* is plotted as perfect concordance between wastewater and human resistance. Dashed lines of *y=x*+0.1 and *y=*x-0.1 represent high agreement, i.e. ±10% from perfect concordance respectively.


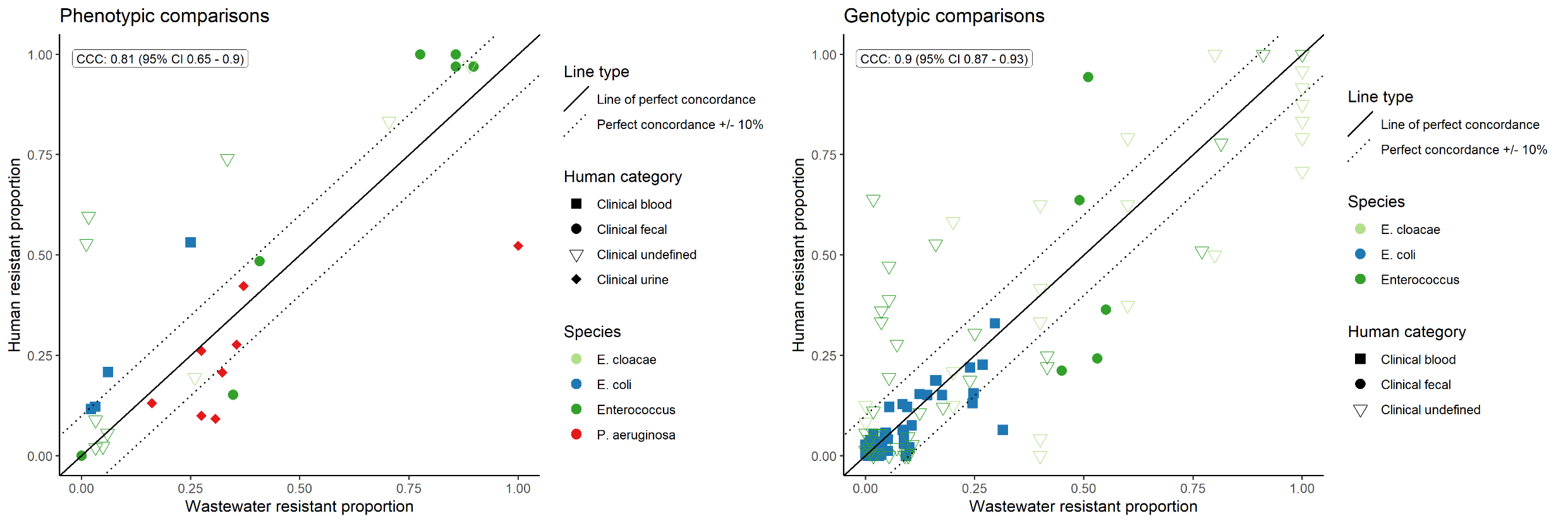


**
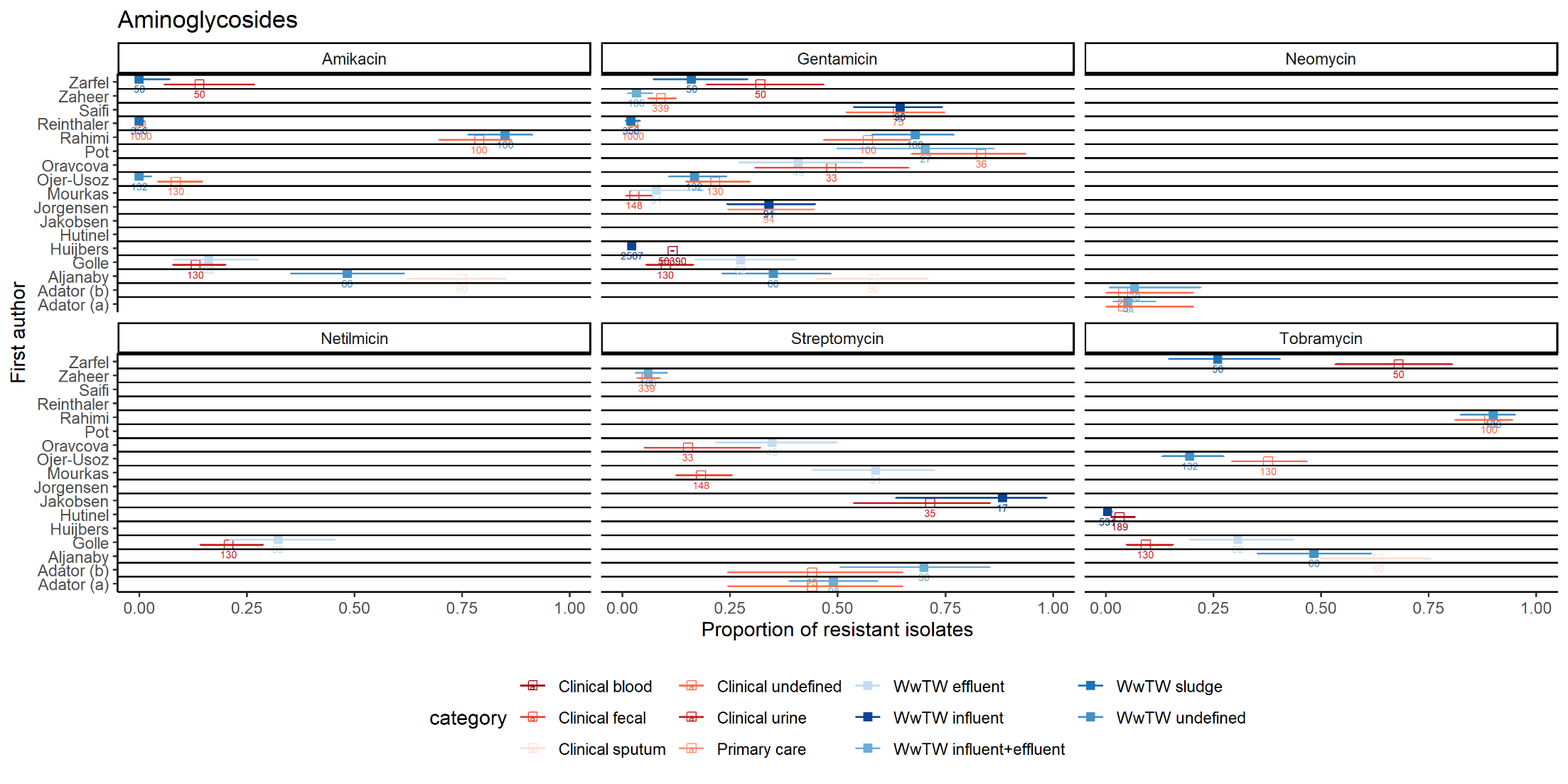
Figure S7: Phenotypic aminoglycoside resistance prevalence estimates in human and wastewater isolates for aminoglycosides by study**Point estimates with 95% confidence intervals plotted for human isolates (hollow squares coloured human sample category) and wastewater isolates (filled squares coloured by wastewater sample category). Total number of isolates analysed labelled under each estimate.

**
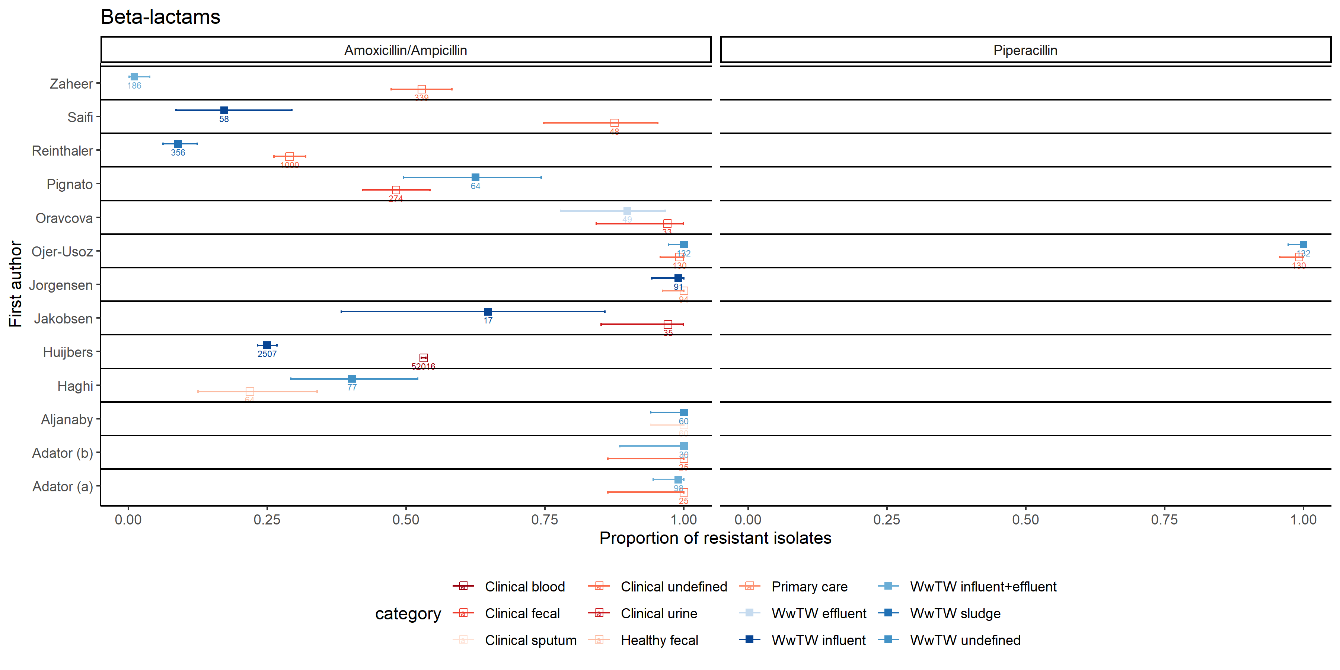
Figure S8: Phenotypic resistance prevalence estimates in human and wastewater isolates for beta-lactams by study**Point estimates with 95% confidence intervals plotted for human isolates (hollow squares coloured human sample category) and wastewater isolates (filled squares coloured by wastewater sample category). Total number of isolates analysed labelled under each estimate.

**
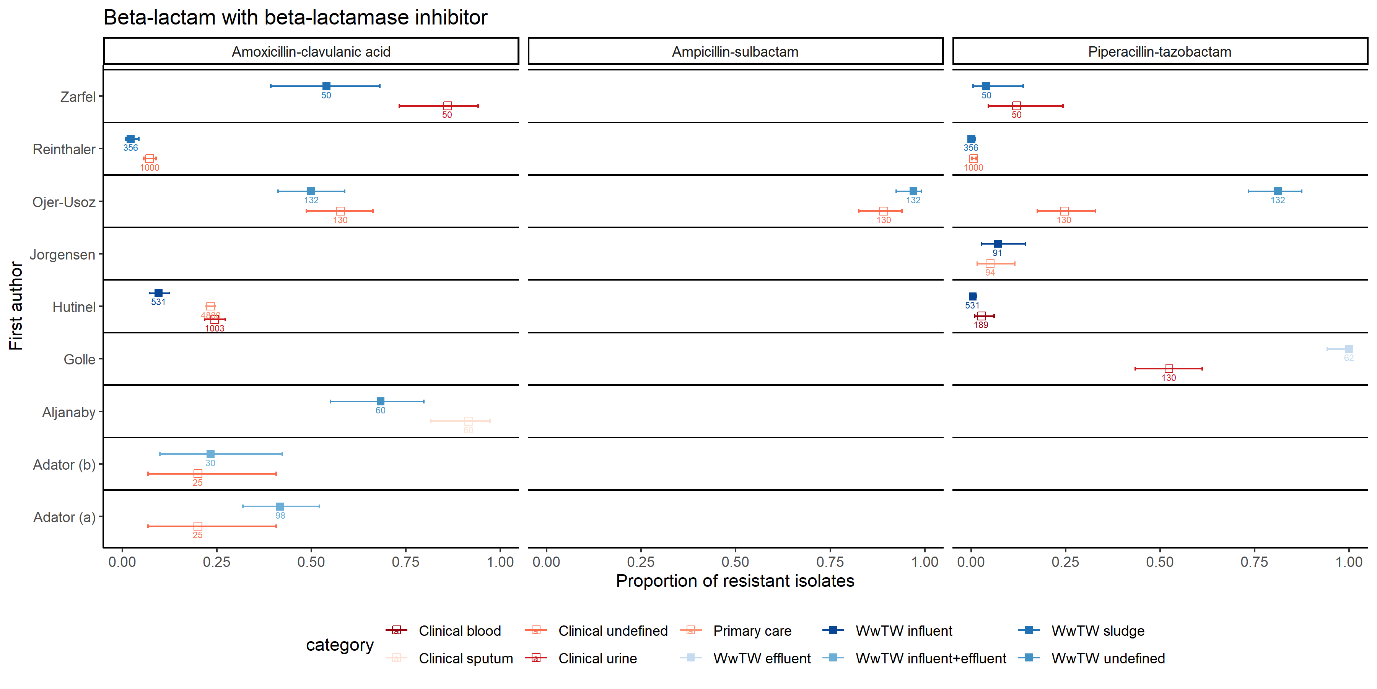
Figure S9: Phenotypic resistance prevalence estimates in human and wastewater isolates for beta-lactam/beta-lactamase inhibitor combinations by study**Point estimates with 95% confidence intervals plotted for human isolates (hollow squares coloured human sample category) and wastewater isolates (filled squares coloured by wastewater sample category). Total number of isolates analysed labelled under each estimate.

**
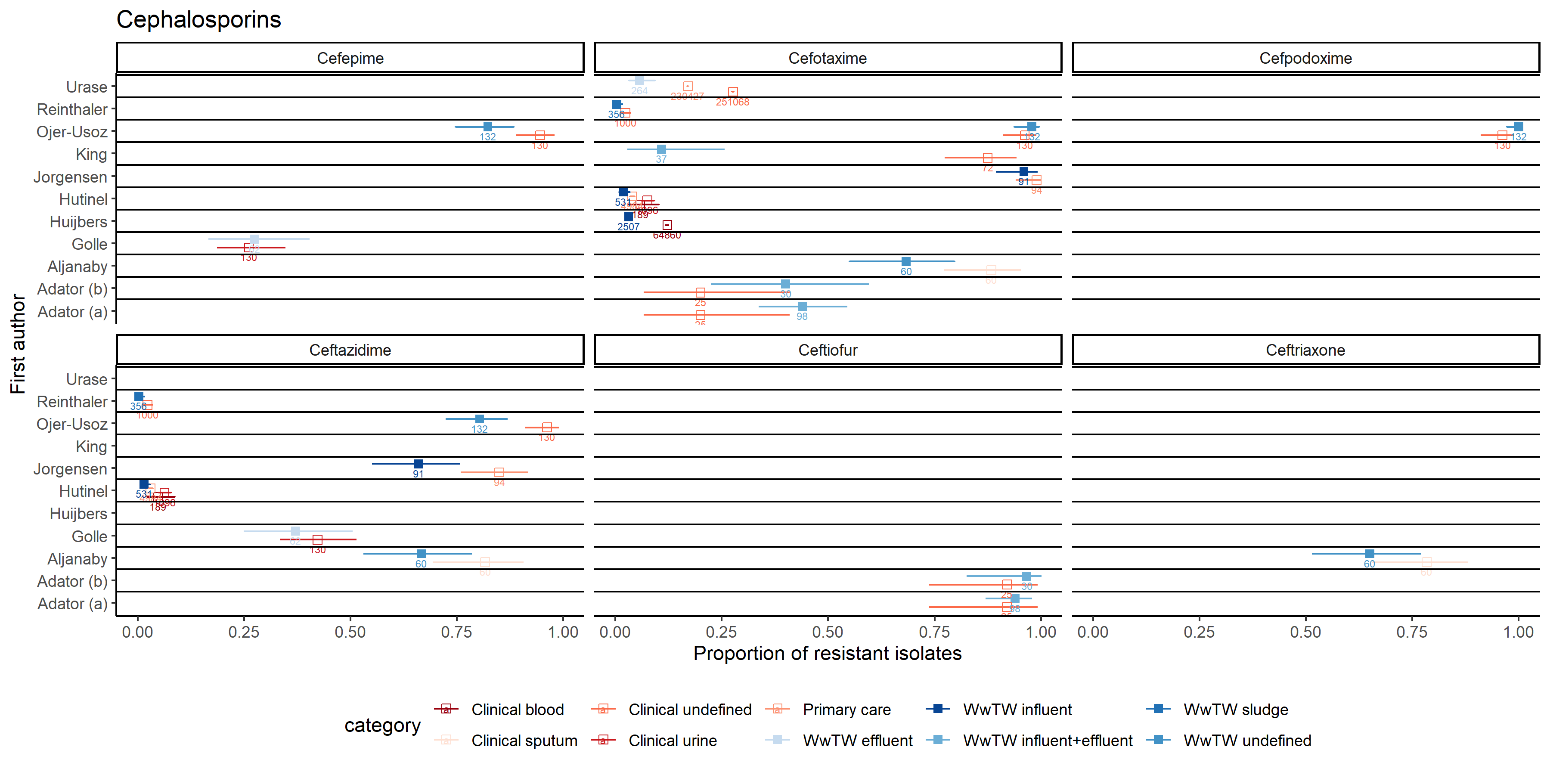
Figure S10: Phenotypic resistance prevalence estimates in human and wastewater isolates for cephalosporins combinations by study**Point estimates with 95% confidence intervals plotted for human isolates (hollow squares coloured human sample category) and wastewater isolates (filled squares coloured by wastewater sample category). Total number of isolates analysed labelled under each estimate.

**
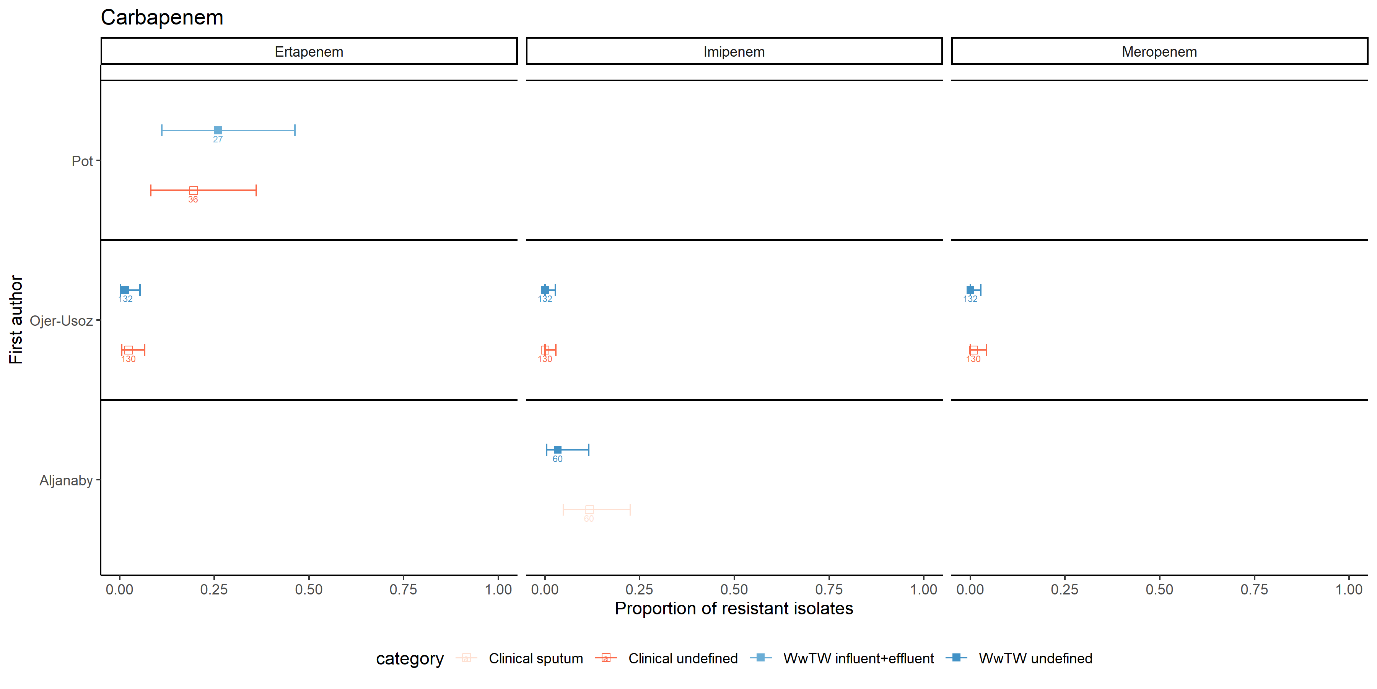
Figure S11: Phenotypic resistance prevalence estimates in human and wastewater isolates for carbapenems by study**Point estimates with 95% confidence intervals plotted for human isolates (hollow squares coloured human sample category) and wastewater isolates (filled squares coloured by wastewater sample category). Total number of isolates analysed labelled under each estimate.

**Figure S12: Phenotypic resistance prevalence estimates in human and wastewater isolates for fluoroquinolones/quinolones by study**Point estimates with 95% confidence intervals plotted for human isolates (hollow squares coloured human sample category) and wastewater isolates (filled squares coloured by wastewater sample category). Total number of isolates analysed labelled under each estimate.

**
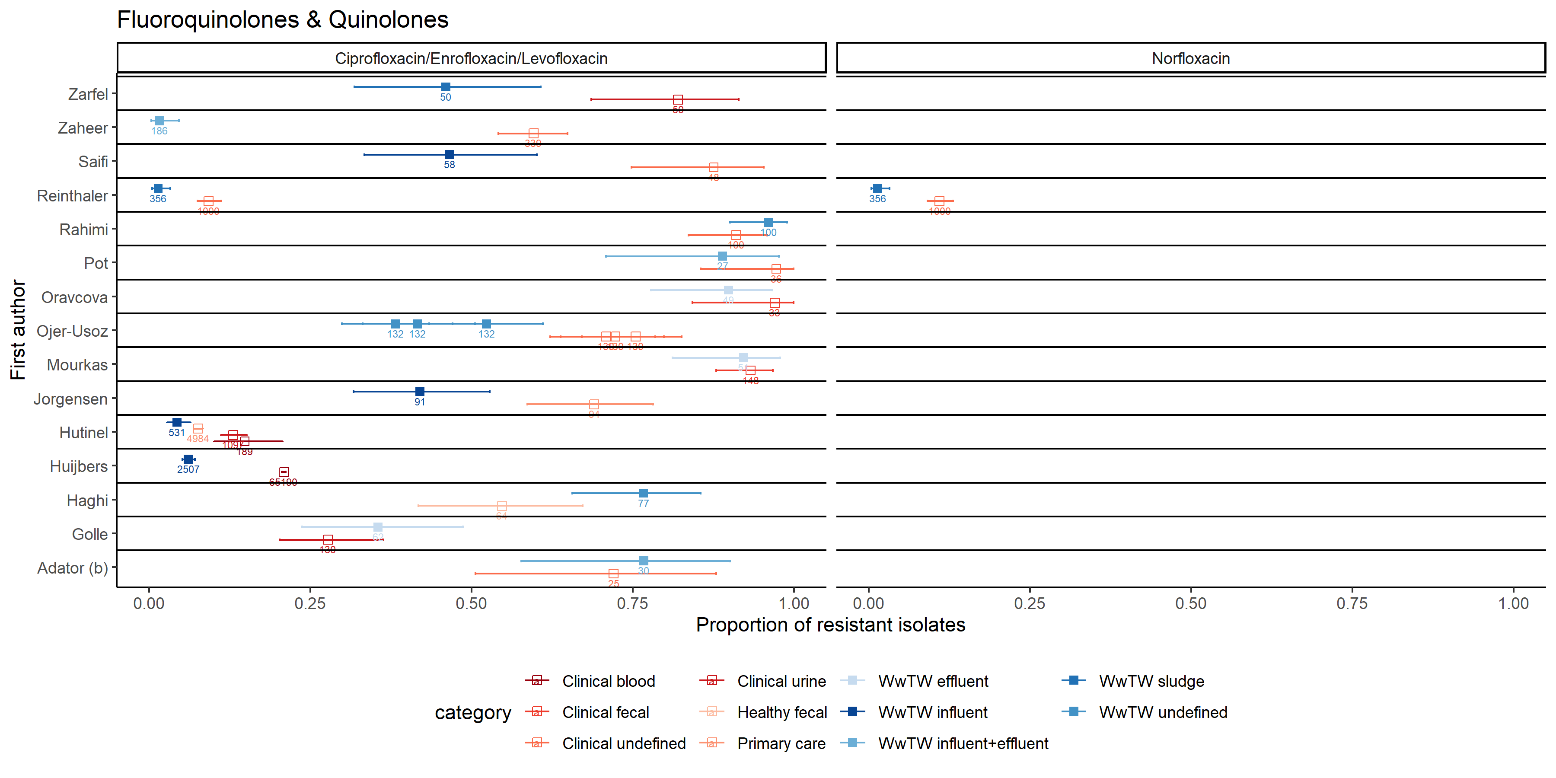
**

**Figure S13: Phenotypic resistance prevalence estimates in human and wastewater isolates for glycopeptides by study**Point estimates with 95% confidence intervals plotted for human isolates (hollow squares coloured human sample category) and wastewater isolates (filled squares coloured by wastewater sample category). Total number of isolates analysed labelled under each estimate.

**
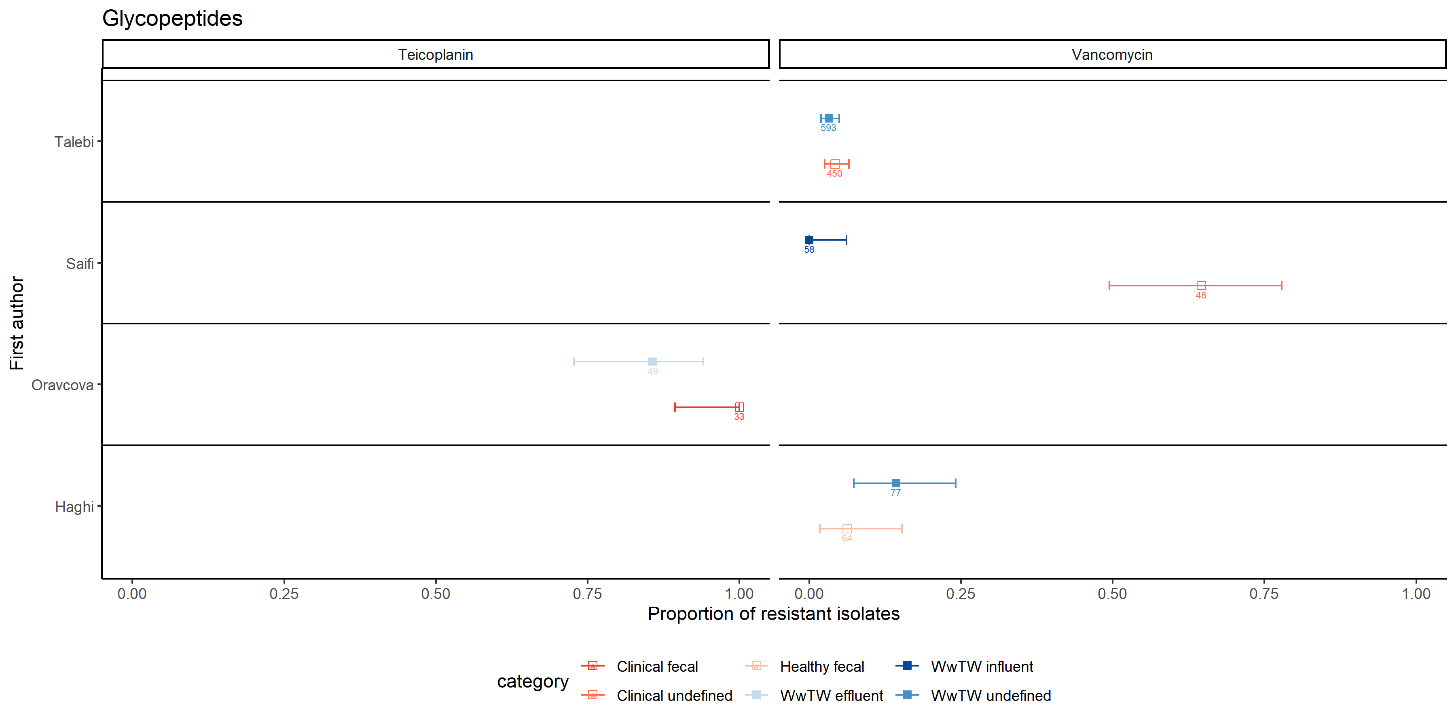
**

**Figure S14: Phenotypic resistance prevalence estimates in human and wastewater isolates for macrolides by study**Point estimates with 95% confidence intervals plotted for human isolates (hollow squares coloured human sample category) and wastewater isolates (filled squares coloured by wastewater sample category). Total number of isolates analysed labelled under each estimate.

**
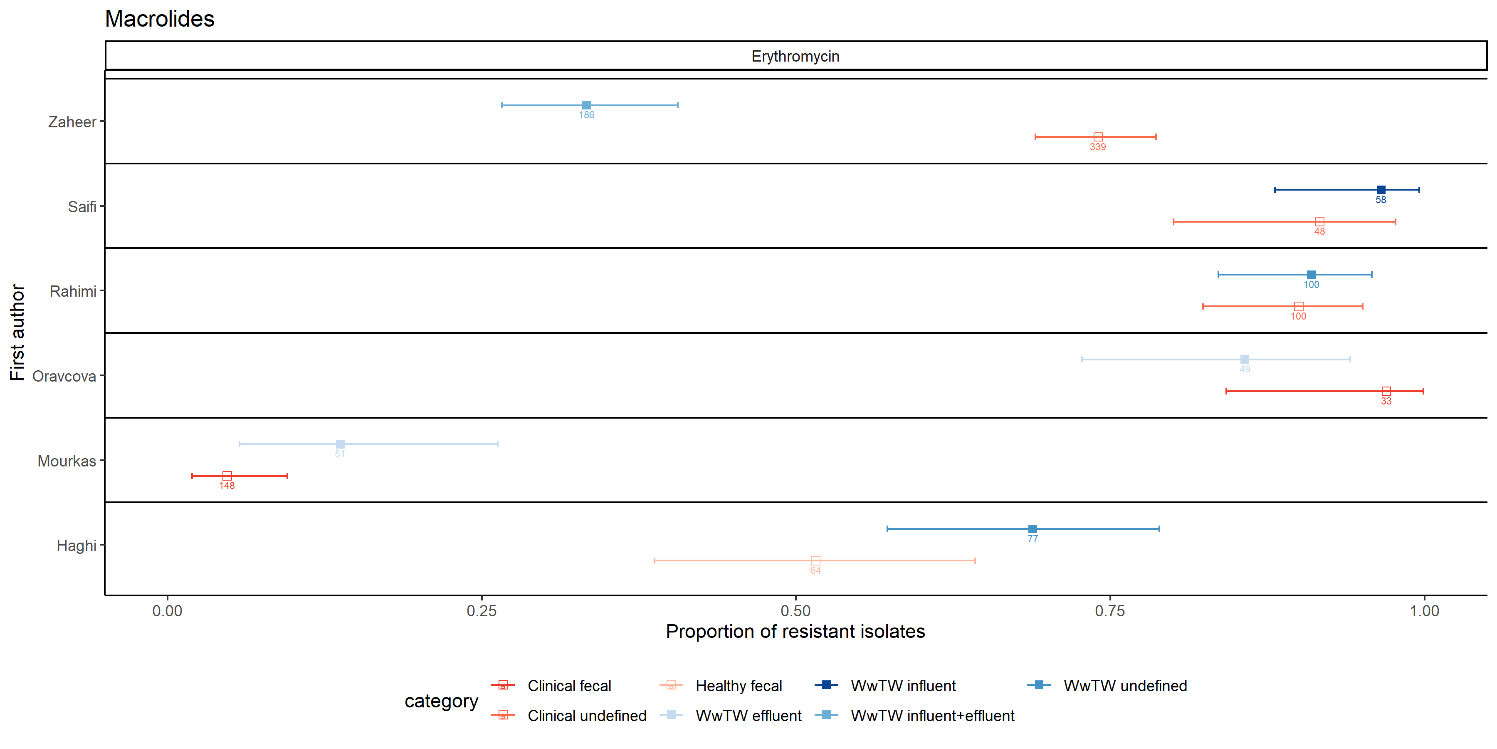
**

**
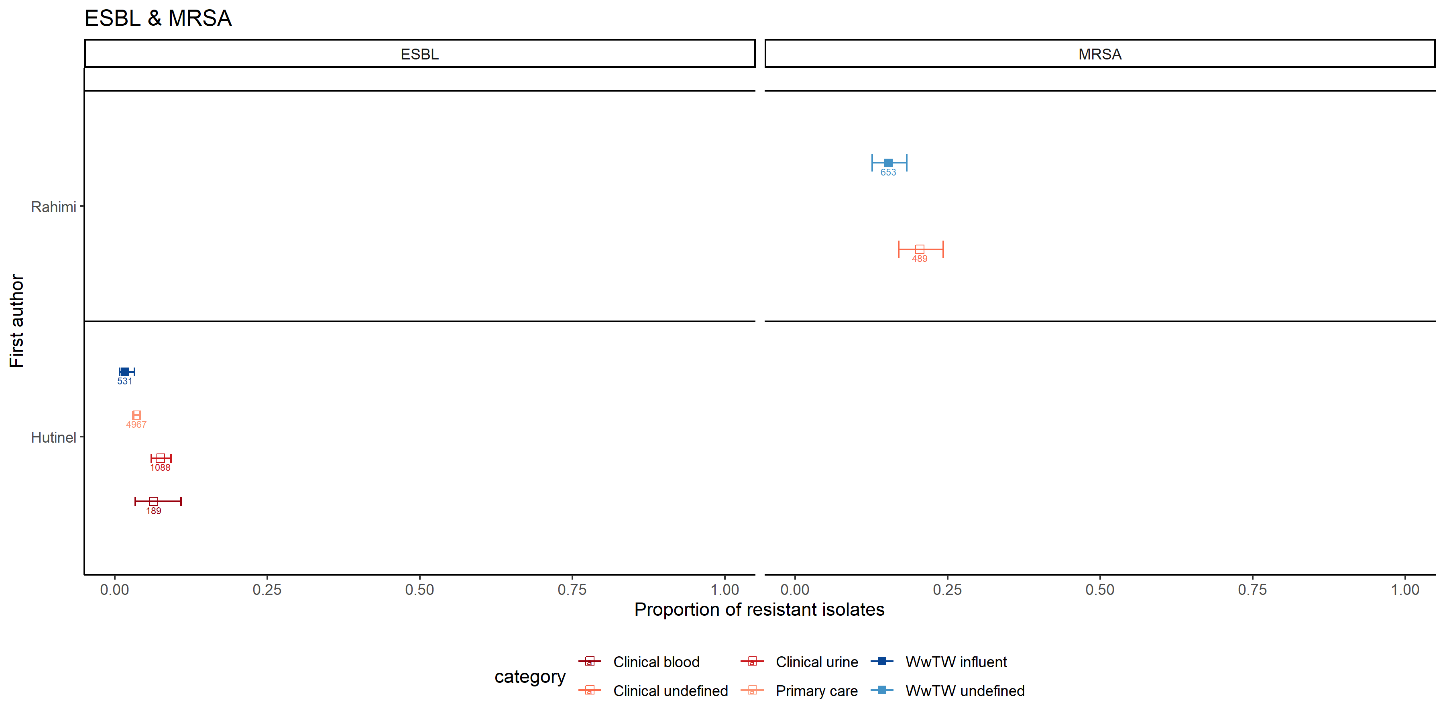
Figure S15: Phenotypic resistance prevalence estimates in human and wastewater isolates for ESBLs and MRSA by study**Point estimates with 95% confidence intervals plotted for human isolates (hollow squares coloured human sample category) and wastewater isolates (filled squares coloured by wastewater sample category). Total number of isolates analysed labelled under each estimate.

**
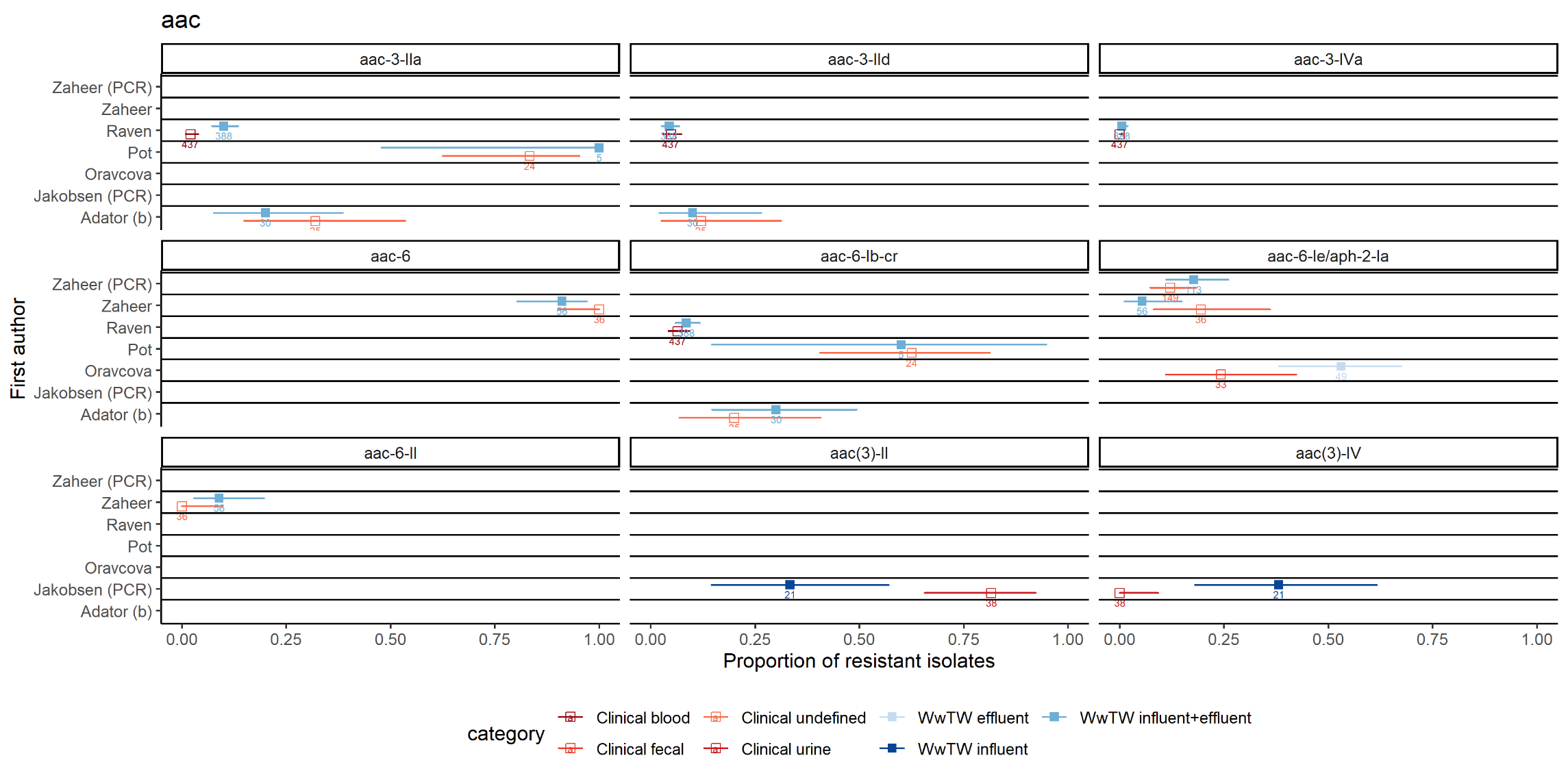
Figure S16: Genotypic prevalence estimates in human and wastewater isolates for *aac* variants by study**Point estimates with 95% confidence intervals plotted for human isolates (hollow squares coloured human sample category) and wastewater isolates (filled squares coloured by wastewater sample category). Total number of isolates analysed labelled under each estimate.

***
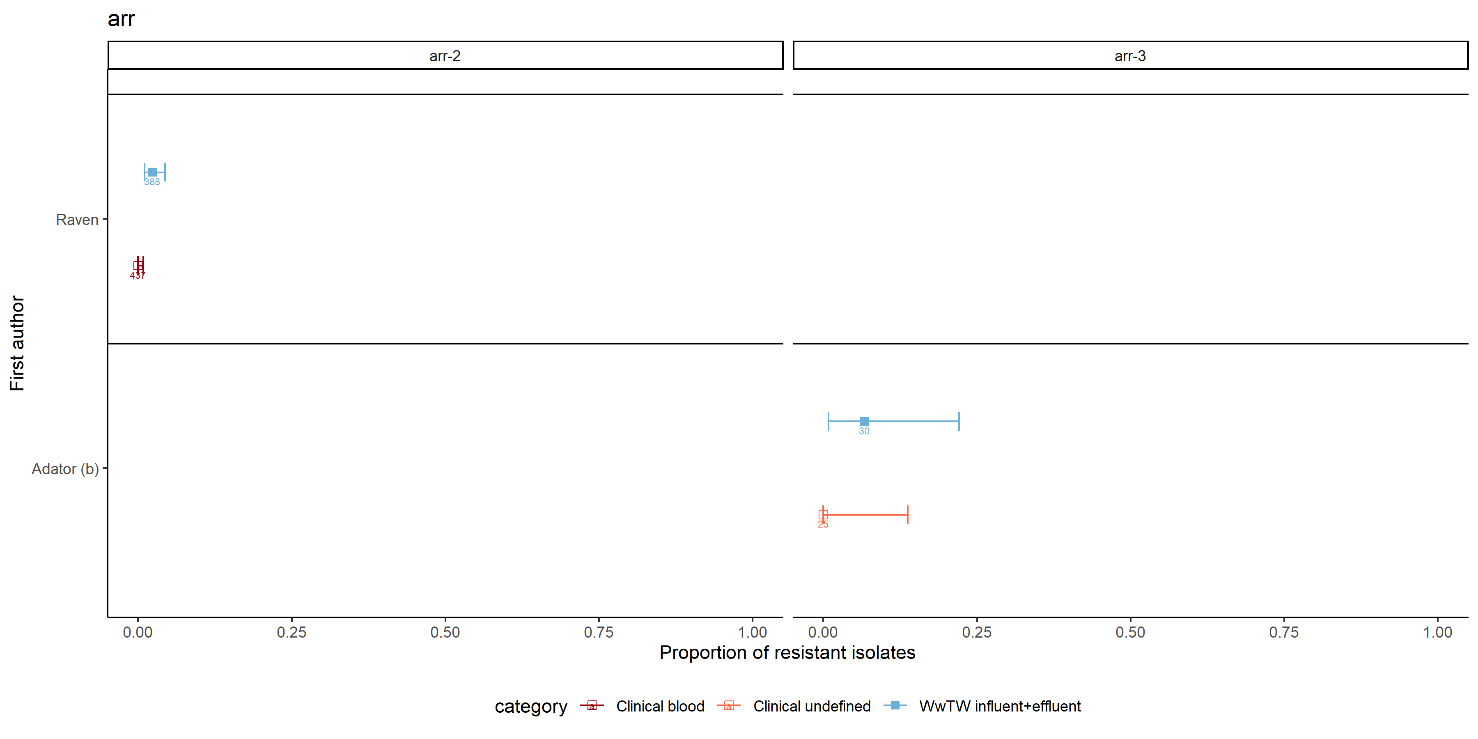
*Figure S17: Genotypic prevalence estimates in human and wastewater isolates for *arr* variants by study**Point estimates with 95% confidence intervals plotted for human isolates (hollow squares coloured human sample category) and wastewater isolates (filled squares coloured by wastewater sample category). Total number of isolates analysed labelled under each estimate.

***
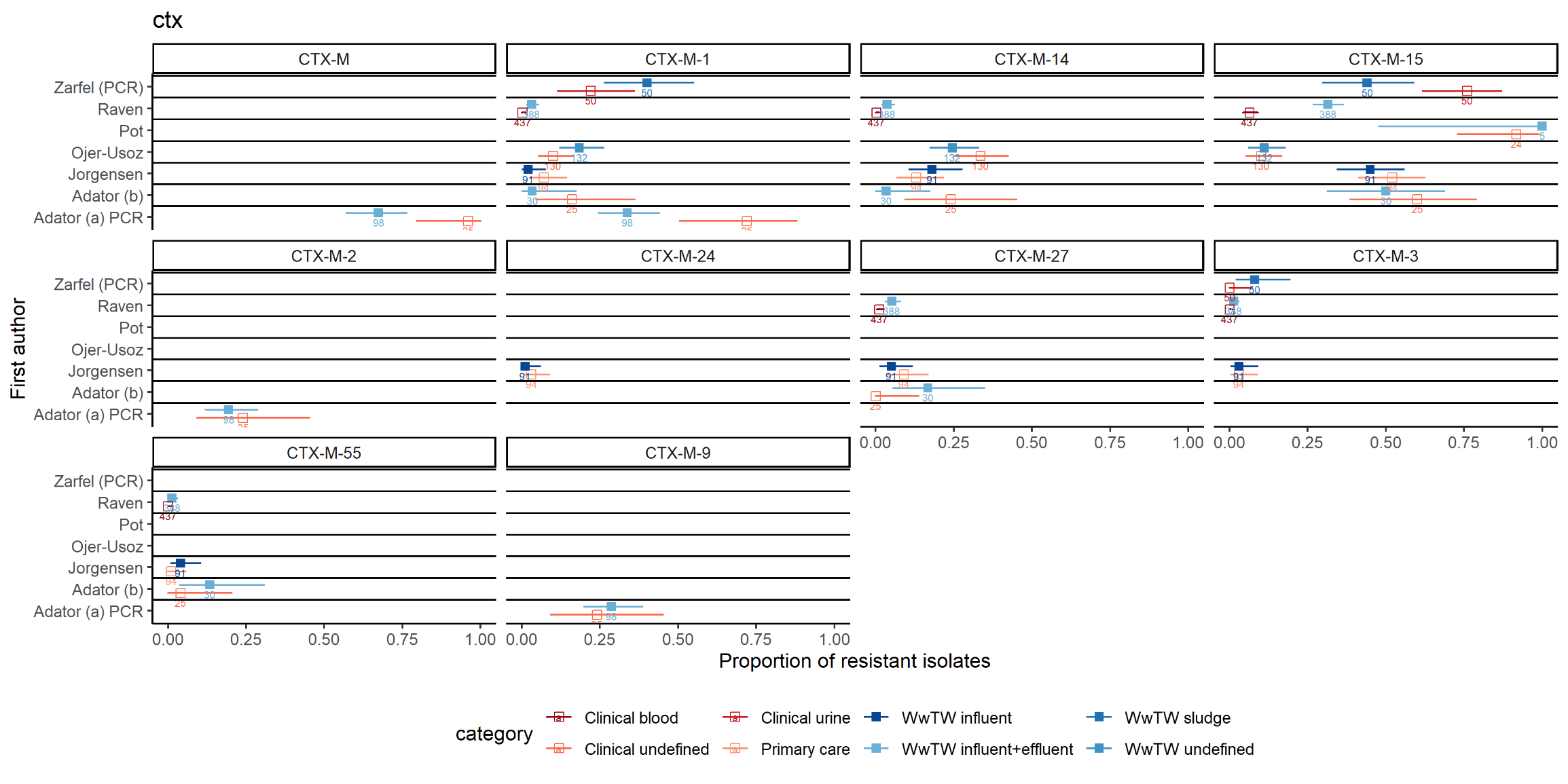
*Figure S18: Genotypic prevalence estimates in human and wastewater isolates for *ctx* variants by study**Point estimates with 95% confidence intervals plotted for human isolates (hollow squares coloured human sample category) and wastewater isolates (filled squares coloured by wastewater sample category). Total number of isolates analysed labelled under each estimate.

***
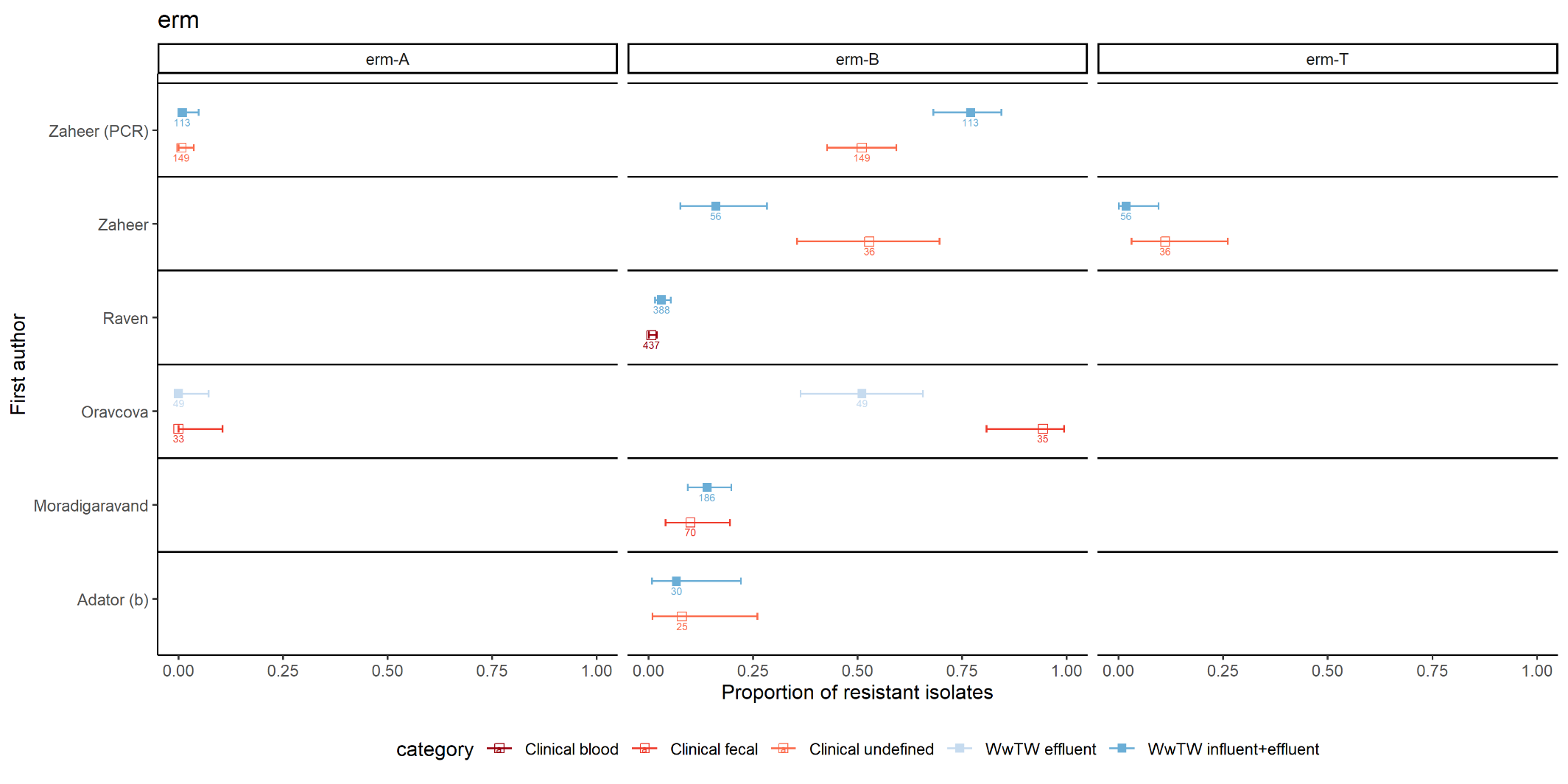
*Figure S19: Genotypic prevalence estimates in human and wastewater isolates for *erm* variants by study**Point estimates with 95% confidence intervals plotted for human isolates (hollow squares coloured human sample category) and wastewater isolates (filled squares coloured by wastewater sample category). Total number of isolates analysed labelled under each estimate.

***
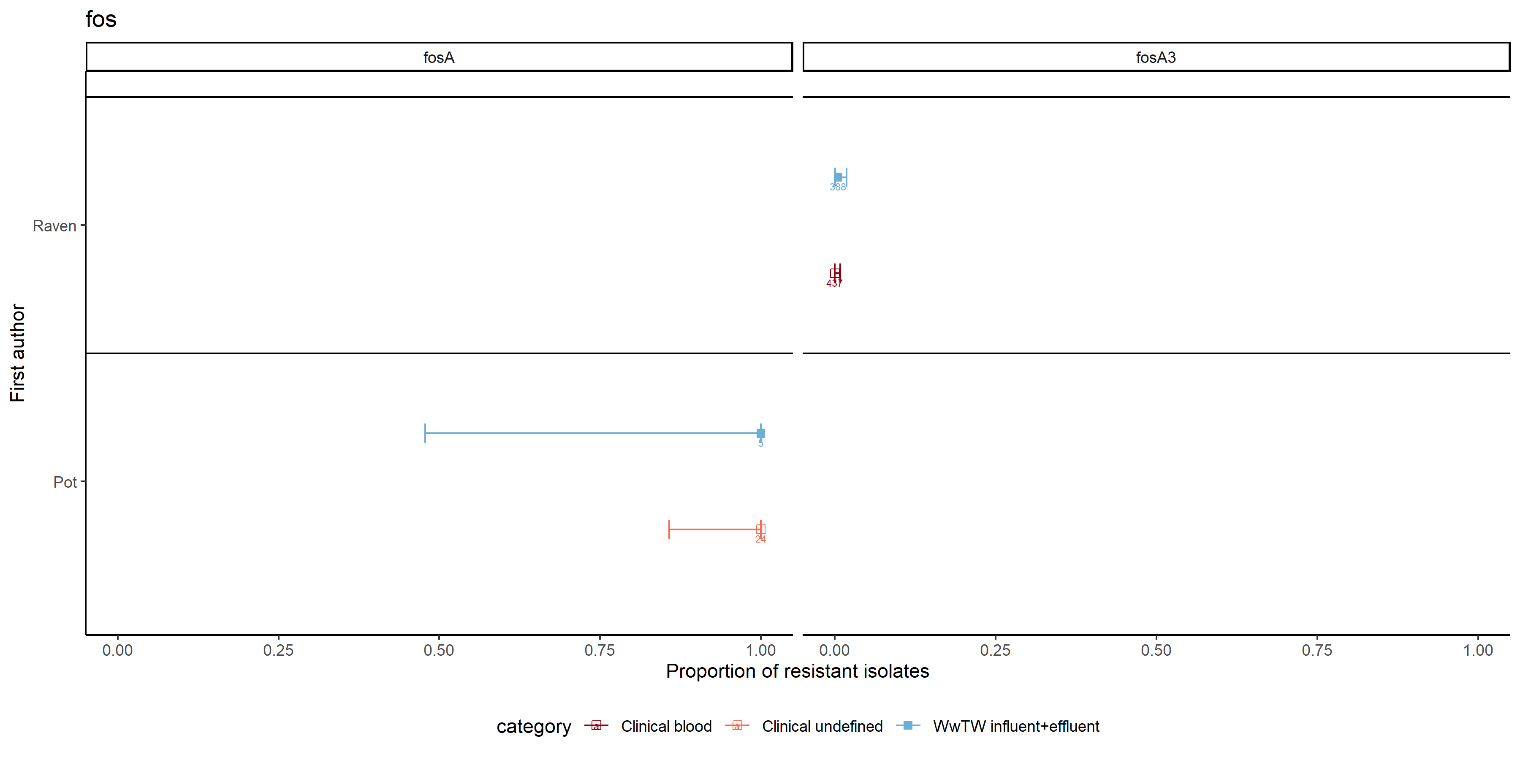
*Figure S20: Genotypic prevalence estimates in human and wastewater isolates for *fos* variants by study**Point estimates with 95% confidence intervals plotted for human isolates (hollow squares coloured human sample category) and wastewater isolates (filled squares coloured by wastewater sample category). Total number of isolates analysed labelled under each estimate.

**
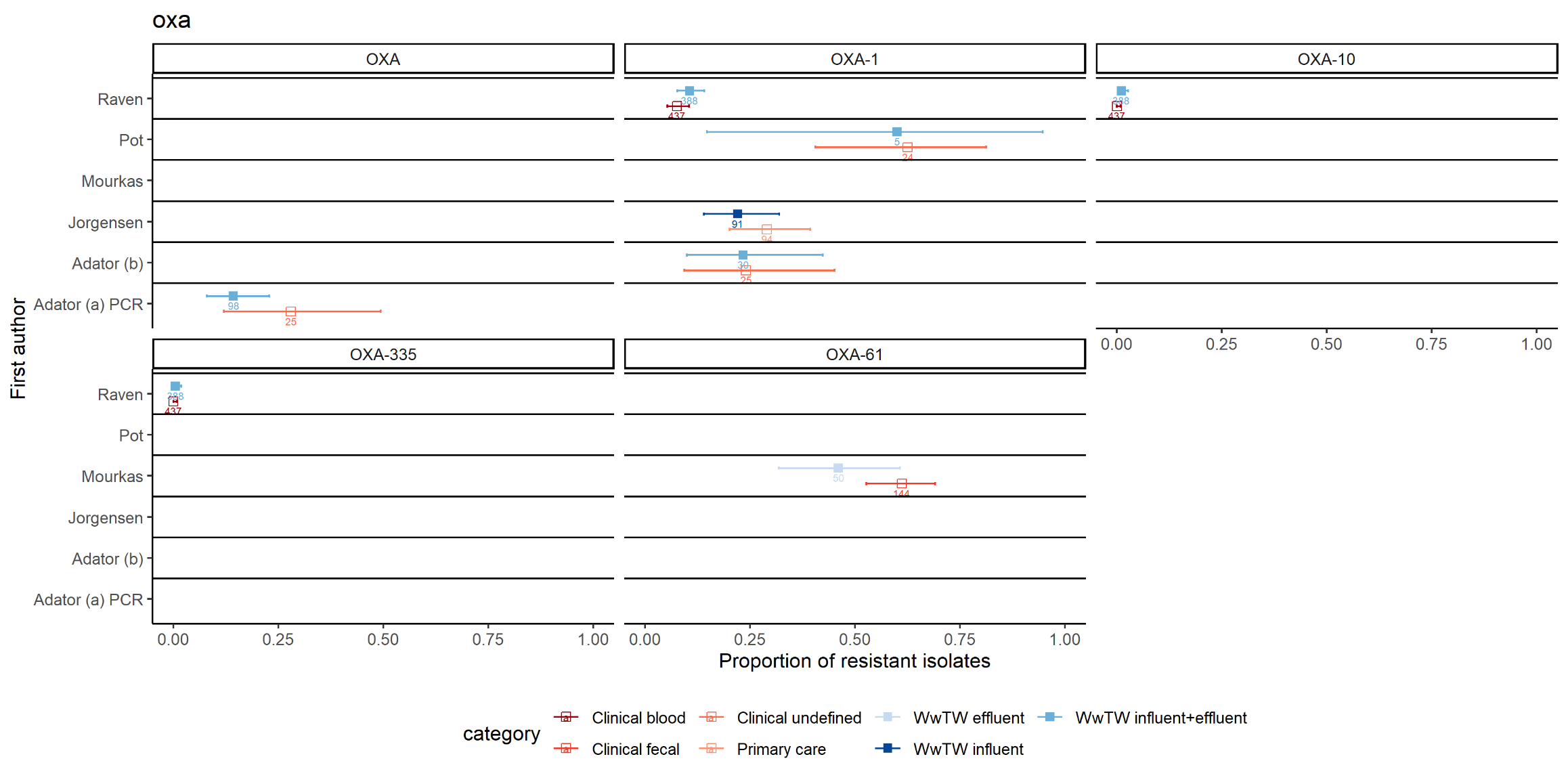
Figure S21: Genotypic prevalence estimates in human and wastewater isolates for *oxa* variants by study**Point estimates with 95% confidence intervals plotted for human isolates (hollow squares coloured human sample category) and wastewater isolates (filled squares coloured by wastewater sample category). Total number of isolates analysed labelled under each estimate.

***
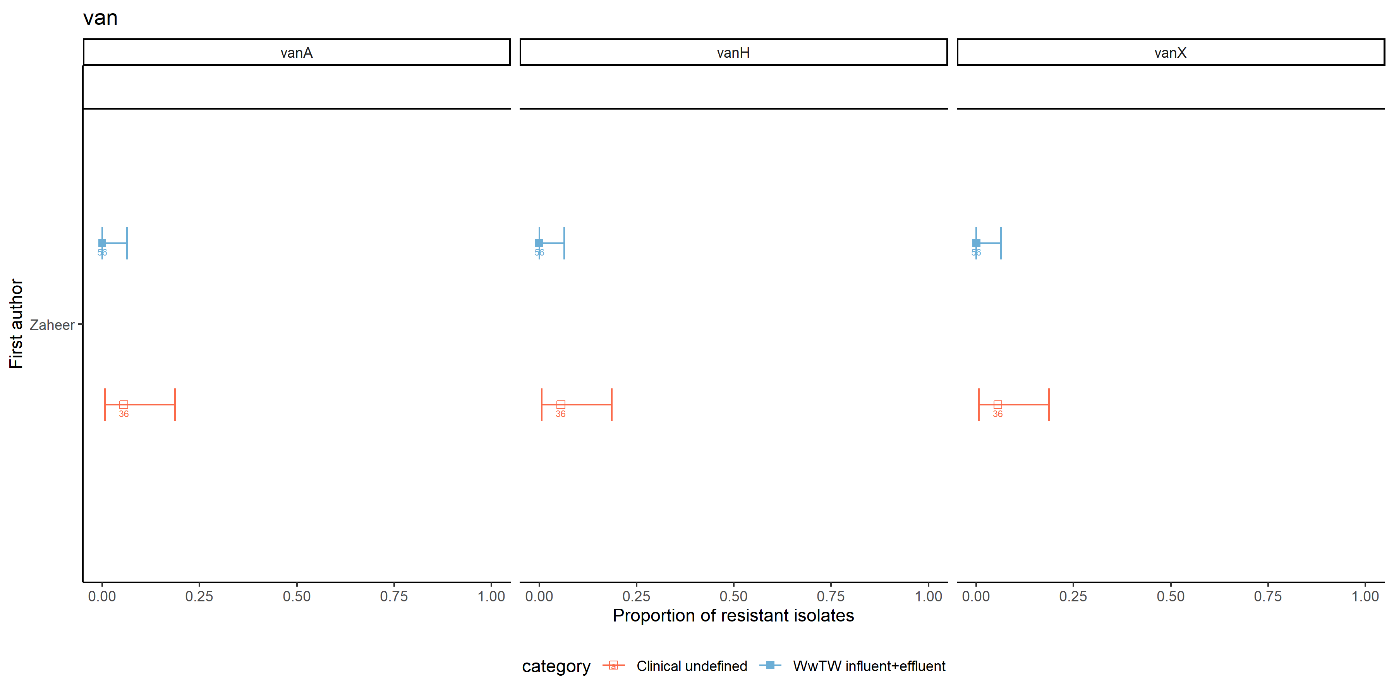
*Figure S22: Genotypic prevalence estimates in human and wastewater isolates for *van* variants by study**Point estimates with 95% confidence intervals plotted for human isolates (hollow squares coloured human sample category) and wastewater isolates (filled squares coloured by wastewater sample category). Total number of isolates analysed labelled under each estimate.

***
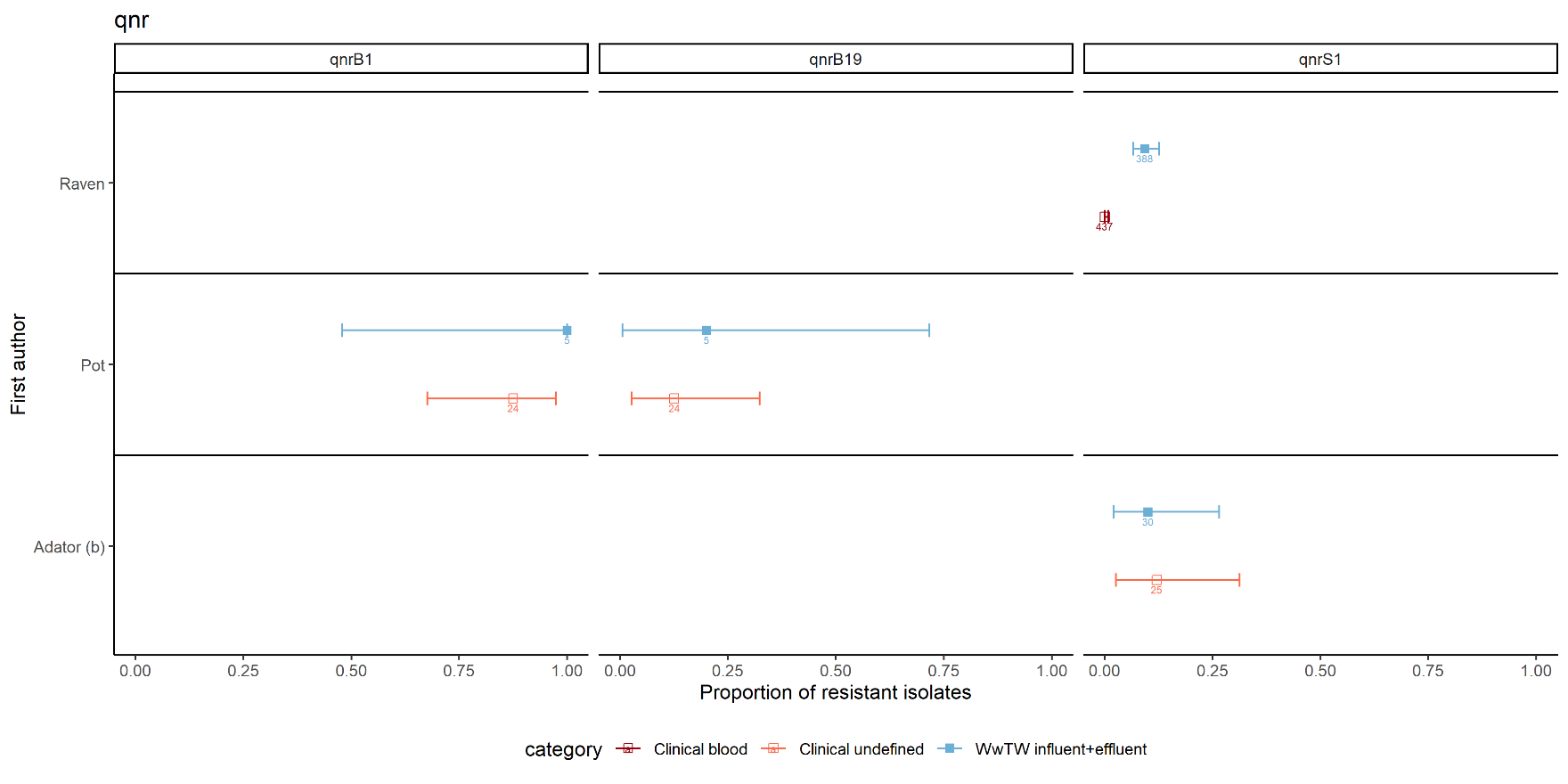
*Figure S23: Genotypic prevalence estimates in human and wastewater isolates for *qnr* variants by study**Point estimates with 95% confidence intervals plotted for human isolates (hollow squares coloured human sample category) and wastewater isolates (filled squares coloured by wastewater sample category). Total number of isolates analysed labelled under each estimate.

**Table S24: Univariable logistic regression of study features associated with high-agreement.**

| **Study feature** | **n (% high) or IQR total (IQR high)** | **OR (95% CI)** | **p** |
| --- | --- | --- | --- |
| **Study design** | 24/24 reported | 1.61 (0.34-7.69) | 0.55 |
| *longitudinal* | 19/24 (32%) |  |  |
| *snapshot* | 4/24 (75%) |  |  |
| *both* | 1/24 (0%) |  |  |
| **Target species** | 24/24 reported | 0.97 (0.69-1.38) | 0.88 |
| *C. difficile* | 1/24 (100%) |  |  |
| *Campylobacter spp.* | 1/24 (0%) |  |  |
| *E. coli* | 11/24 (45%) |  |  |
| *E. cloacae* | 1/24 (0%) |  |  |
| *Enterococcus spp.* | 5/24 (40% |  |  |
| *Klebsiella spp.* | 1/24 (0%) |  |  |
| *P. aeruginosa* | 2/24 (0%) |  |  |
| *S. aureus* | 1/24 (100%) |  |  |
| *Salmonella spp.* | 1/24 (0%) |  |  |
| **AMR detection approach of extracted data** | 24/24 reported | 2.59 (0.62-10.80) | 0.19 |
| *genotypic* | 2/24 (100%) |  |  |
| *phenotypic* | 12/24 (33%) |  |  |
| *mixed* | 10/24 (30%) |  |  |
| **Human sample type^1^** | 24/24 reported | 0.76 (0.43-1.35) | 0.35 |
| *clinical blood* | 2/24 (50%) |  |  |
| *clinical faecal* | 4/24 (25%) |  |  |
| *clinical sputum* | 1/24 (0%) |  |  |
| *clinical urine* | 3/24 (0%) |  |  |
| *clinical unspecified* | 10/24 (50%) |  |  |
| *faecal carriage* | 1/24 (0%) |  |  |
| *primary care* | 3/24 (67%) |  |  |
| **Number of human isolates analysed** | 24/24 reported | 0.99 (0.99-1.00) | 0.56 |
| *IQRs* | 46.5-314.75 (70-450) |  |  |
| **Region sampled income classification** | 24/24 reported | 0.53 (0.09-2.83) | 0.45 |
| *high* | 17/24 (41%) |  |  |
| *medium* | 6/24 (33%) |  |  |
| *low* | 1/24 (0%) |  |  |
| **Number of wastewater isolates analysed** | 24/24 reported | 1.00 (0.99-1.00) | 0.92 |
| *IQRs* | 50.75-205.5 (91-388) |  |  |
| **Pre-defined surveillance study** | 24/24 reported | 1.14 (0.15-8.59) | 0.89 |
| *yes* | 5/24 (40%) |  |  |
| *no* | 19/24 (37%) |  |  |
| **Number of WwTWs sampled** | 23/24 reported (1 missing) | 1.09 (0.93-1.27) | 0.29 |
| IQRs | 1-5 (1-5) |  |  |
| **Wastewater sampling method** | 20/24 reported (4 missing) | 0.76 (0.34-1.68) | 0.49 |
| *grab* | 9/24 (33%) |  |  |
| *composite* | 6/24 (67%) |  |  |
| *flow-proportional* | 3/24 (67%) |  |  |
| *time-flow-volume-proportional* | 1/24 (0%) |  |  |
| *moore swab* | 1/24 (0%) |  |  |
| *not reported* | 4/24 (0%) |  |  |
| **Wastewater sampled has a direct input from the human population surveyed** | 18/24 reported (6 missing) | 0.44 (0.07-2.69) | 0.38 |
| *yes* | 11/24 (45%) |  |  |
| *no* | 6/24 (33%) |  |  |
| *both* | 1/24 (0%) |  |  |
| *not reported* | 6/24 (33%) |  |  |
| **Wastewater sampling point** | 17/24 reported (5 missing) | 0.93 (0.38-2.26) | 0.87 |
| *influent* | 5/24 (40%) |  |  |
| *effluent* | 4/24 (0%) |  |  |
| *influent & effluent* | 8/24 (57%) |  |  |
| *sludge* | 2/24 (50%) |  |  |
| *not reported* | 5/24 (40%) |  |  |

^1^Primary care was removed from testing due to the uncertainty to which this category belongs in respect to other sample types. Sensitivity analysis including primary care showed no significant difference (p=0.363)

**Table S25: Study features excluded from logistic regression due to missingness**

| **Study feature** | **n (% high) or IQR total (IQR high)** |
| --- | --- |
| **Wastewater samples undergone freeze-thawing?** | 14/24 reported (10 missing) |
| *no* | 14/24 (36%) |
| *not reported* | 10/24 (40%) |
| **Presence of unique sewer inputs** | 12/24 reported (12 missing) |
| *agriculture* | 2/24 (100%) |
| *healthcare* | 7/24 (29%) |
| *healthcare and industry* | 2/24 (0%) |
| *no unique sewer inputs* | 1/24 (100%) |
| *not reported* | 12/24 (33%) |
| **Wastewater storage temperature** | 10/24 reported (14 missing) |
| *4°C* | 10/24 (40%) |
| *not reported* | 14/24 (36%) |
| **Wastewater sample time-to-processing (hours)** | 8/24 reported (16 missing) |
| IQRs | 3-15 (8.25-18.75) |
| *not reported* | 16/24 (44%) |
| **WwTW population equivalent** | 7/24 reported (17 missing) |
| IQRs | 11722-186837.5 (10261-140867) |
| *not reported* | 17/24 (41%) |
| **WwTW sampling point flow rate (m^3^ day)** | 6/24 reported (18 missing) |
| IQRs | 1987.2-51861.6 (5464.8-54086.4) |
| *not reported* | 18/24 (44%) |
| **Weather conditions during wastewater sampling** | 2/24 reported (22 missing) |
| *specifically avoiding rain events* | 1/24 (0%) |
| *mixed but mostly during rain events* | 1/24 (0%) |
| *not reported* | 22/24 (38%) |

| **Table S26: Tally of features and agreement** | | Overall study agreement | | |
| --- | --- | --- | --- | --- |
|  | Study features | <30% | 30-70% | >70% |
| Sampling point | influent | 2 | 3 | 2 |
|  | influent & effluent | 2 | 5 | 5 |
|  | effluent | 1 | 4 | 1 |
|  | sludge | 1 | 1 | 1 |
|  | not reported | 2 | 2 | 2 |
| Study design | longitudinal | 7 | 14 | 8 |
|  | snapshot | 0 | 1 | 3 |
|  | both | 1 | 0 | 0 |
| Longitudinal timeframe | <6 months | 2 | 1 | 1 |
|  | 6-12 months | 5 | 3 | 1 |
|  | >12 months | 1 | 8 | 6 |
|  | not reported | 0 | 2 | 0 |
|  | NA (snapshot studies) | 0 | 1 | 3 |
| Sampling method | grab | 4 | 4 | 4 |
|  | composite | 1 | 3 | 5 |
|  | flow-proportional | 2 | 1 | 2 |
|  | flow-proportional+ | 0 | 1 | 0 |
|  | moore swab | 0 | 2 | 0 |
|  | not reported | 1 | 4 | 0 |
| Comparison type | direct | 4 | 8 | 6 |
|  | indirect | 1 | 4 | 3 |
|  | both | 1 | 0 | 0 |
|  | unclear | 2 | 3 | 2 |
| Sewer AMR input | agriculture | 0 | 1 | 3 |
|  | hospital | 2 | 5 | 2 |
|  | hospital & industry | 2 | 0 | 1 |
|  | none | 0 | 0 | 1 |
|  | not reported | 4 | 8 | 5 |
| Number of WwTWs | 1 | 3 | 6 | 4 |
|  | 2 | 2 | 4 | 3 |
|  | 3-10 | 2 | 3 | 2 |
|  | >10 | 1 | 0 | 2 |
|  | not reported | 0 | 2 | 0 |
| Human category | clinical blood | 0 | 1 | 1 |
|  | clinical urine | 3 | 2 | 0 |
|  | clinical sputum | 1 | 0 | 0 |
|  | clinical fecal | 1 | 3 | 2 |
|  | clinical undefined | 1 | 8 | 6 |
|  | primary care | 1 | 1 | 2 |
|  | healthy fecal | 1 | 0 | 0 |
| Surveillance study | yes | 2 | 1 | 2 |
|  | no | 6 | 14 | 9 |

**Complete Author contributions (as per CRediT; Contributor Roles Taxonomy)**

**Conceptualization**

KK Chau, DS Read, N Stoesser, AS Walker

**Methodology**

KK Chau, E Harriss, DS Read, N Stoesser, AS Walker

**Software**

**N/A**

**Validation**

L Barker, EP Budgell, KK Chau, E Harriss, N Sims,

**Formal Analysis**

KK Chau, N Stoesser, Vihta KD

**Investigation**

L Barker, KK Chau, N Sims, N Stoesser

**Resources**

KK Chau, EP Budgell, E Harriss

**Data curation**

KK Chau, E Harriss, N Stoesser

**Writing – original draft**

KK Chau, DS Read, N Stoesser

**Writing – review & editing**

L Barker, EP Budgell, KK Chau, E Harriss, B Kasprzyk-Hordern, DS Read, N Sims, N Stoesser, AS Walker

**Visualization**

KK Chau, N Stoesser

**Supervision**

KK Chau, DW Crook, DS Read, N Stoesser, AS Walker

**Project administration**

KK Chau, DS Read, N Stoesser, AS Walker

**Funding acquisition**

KK Chau, DW Crook, DS Read, N Stoesser, AS Walker
