# Supplementary material for: Systematic review of wastewater surveillance of antimicrobial resistance in human populations
Source: Environ Int. 2022 Apr;162:107171. doi: 10.1016/j.envint.2022.107171 (PMC8960996; doi:10.1016/j.envint.2022.107171)
Supplement: Supplementary data 2 [file mmc2.docx]

**Title of submitted paper and corresponding author**: Systematic review of wastewater surveillance of antimicrobial resistance in human populations (Kevin Chau)

| **#** | **Item** | **Guidance** | **On page #** | **Author Comments** |
| --- | --- | --- | --- | --- |
| **Title** | | | | |
| 1 | Title | Identify the report as a systematic review, or systematic review and meta-analysis, as appropriate. | 1 | “Systematic review of wastewater surveillance of antimicrobial resistance in human populations” |
| **Abstract** | | | | |
| 2 | Structured summary | Provide a structured summary including, as applicable:   - Background; - Objectives; - Data sources; - Study eligibility criteria, participants, and interventions; - Study appraisal and synthesis methods; - Results; - Limitations; conclusions and implications of key findings; - Systematic review registration number. | 3,4 | Abstract structured around Objectives, Results and Conclusion. |
| **Introduction** | | | | |
| 3 | Rationale | Describe the rationale for the review in the context of what is already known. | 6-8; Table 1 | Lines 107-130 “However, heterogeneous study designs and methods likely contribute to differences in outcomes/interpretations. The impact of methodological approaches such as grab sampling (i.e. taking single samples at a single timepoint (Reinthaler et al., 2013), snapshot versus longitudinal study design, sampling in the presence of unrepresentative and “contaminating” AMR-associated point sources, and/or characterising AMR based on phenotypic testing of isolates versus genotypic profiling remains poorly understood (Table 1).” Cont. |
| 4 | Objectives | Provide an explicit Population-Intervention-Comparator-Outcome-Study Design (PICOS) or Population-Exposure-Comparator-Outcome-Study Design (PECOS) statement as appropriate, detailing the following in relation to the research questions being asked:   - Participants - Interventions / Exposures (as appropriate) - Comparisons - Outcomes - Study design | 9 | Lines 134-141 “For this systematic review, we sought firstly to evaluate concordance between wastewater and human AMR prevalence estimates for each study, stratified by the AMR detection method used (i.e. phenotypic versus genotypic). Secondly, we adapted the PECOTS (Population, Exposure/Intervention, Comparator, Outcome, Target Condition, Study Design) systematic review framework and formulated the following statement to assess association between study methods and outcomes: Among studies jointly evaluating AMR prevalence in wastewater and humans, what is the effect of methodological approaches (e.g. wastewater sampling methods, AMR detection methods) on the concordance between these metrics?” |
| **Methods** | | | | |
| 5 | Protocol and registration | Indicate if a review protocol exists, if and where it can be accessed (e.g. web address), and registration information including registration number (if available). | 9 | Lines 144-146 “the complete PROSPERO protocol is available at: https://www.crd.york.ac.uk/prospero/display_record.php?ID=CRD42019134946.” |
| 6 | Eligibility criteria | Specify study characteristics (e.g. PICOS/PECOS, length of exposure) and report characteristics (e.g. years considered, language, publication status) used as criteria for eligibility, giving rationale. | 10, Fig. S1, Supplementary dataset 3 | Lines 158-160 “Records were assessed through a two-stage screen detailed in (Fig.S1, Supplementary dataset 3) to capture both studies piloting wastewater-based AMR surveillance and studies conducting relevant wastewater-human AMR comparisons.” Cont. |
| 7 | Information sources | Describe all information sources (e.g. databases with dates of coverage, contact with study authors to identify additional studies) in the search, and date last searched. | 9 | Lines 141-147  “Searches were conducted on 01/02/2019 in: MEDLINE, EMBASE, Global Health, CAB Abstracts, Scopus and Web of Science Core Collection. Searches were updated on 09/01/2021 using identical search strings” |
| 8 | Search | Present full electronic search strategy for at least one database, including any limits used, such that it could be repeated. | 9, Supplementary dataset 2 | Lines 148-155 “The search string was developed through iterative preliminary searches in consultation with a librarian experienced with systematic reviews. Full search strings adapted for each database are presented in (Supplementary dataset 2). |
| 9 | Study selection | State the process for selecting studies (i.e., screening, eligibility, included in systematic review, and, if applicable, included in the meta-analysis). | 10, Fig.S1, Supplementary dataset 3 | Lines 158-160 “Records were assessed through a two-stage screen detailed in (Fig.S1, Supplementary dataset 3) to capture both studies piloting wastewater-based AMR surveillance and studies conducting relevant wastewater-human AMR comparisons.” Cont. |
| 10 | Data collection process | Describe method of data extraction from reports (e.g., piloted forms, independently, in duplicate) and any processes for obtaining and confirming data from investigators. | 10, Supplementary dataset 4 | Lines 169-170 “Records were independently screened in duplicate (KKC, LB) and data was extracted from included records using a pre-tested data extraction form piloted on five random included records (Supplementary dataset 4)” |
| 11 | Data items | List and define all variables for which data were sought (e.g., PICOS/PECOS, funding sources) and any assumptions and simplifications made. | 11, Supplementary dataset 4 | Lines 177-179 “Records were independently screened in duplicate (by the authors KKC and LB) and data was extracted from included records using a pre-tested data extraction form piloted on five random included records (Supplementary dataset 4).” Cont. |
| 12 | Risk of bias in individual studies | Describe methods used for assessing risk of bias of individual studies (including specification of whether this was done at the study or outcome level), and how this information is to be used in any data synthesis. | 11-12, Supplementary dataset 5, Table S2 | Lines 192-211  “Risk of bias was assessed independently by two reviewers (the authors KKC and LB) using a qualitative approach based on the Cochrane risk of bias tool (Higgins et al., 2011);our modified tool focused on systematic differences at the study level as outcomes reported were highly diverse.” Cont. |
| 13 | Summary measures | State the principal summary measures (e.g., risk ratio, difference in means). | 13-14 | Lines 223-232 “. As perfect concordance is unrealistic, we arbitrarily defined “high concordance” to represent a ±10% difference in AMR prevalence between wastewater and human compartments.” Cont. |
| 14 | Synthesis of results | Describe the methods of handling data and combining results of studies, if done, including measures of consistency (e.g., I^2^) for each meta-analysis. | 13 | Lines 220-223  “For extracted resistance prevalence data, we used Lin’s concordance correlation coefficient (CCC – R package DescTools) with 95% confidence intervals (CIs) to quantify the concordance between the proportion of resistant wastewater isolates (i.e. wastewater AMR prevalence) and the proportion of resistant human isolates (i.e. human AMR prevalence), with the latter representing the reference standard.” Cont. |
| 15 | Risk of bias across studies | Specify any assessment of risk of bias that may affect the cumulative evidence (e.g., publication bias, selective reporting within studies). | 12-13, Supplementary dataset 5, Table S3 and S4 | Lines 208-228 “an overall qualitative measure (high, low and unclear) was assigned to each study; as per the Cochrane risk of bias tool approach to summary assessment (Higgins et al., 2011).  Certainty assessment (assessment of overall confidence in the evidence included) was conducted using an adaptation (Woodruff and Sutton, 2011) of the GRADE (Grading of Recommendations, Assessment” Cont. |
| 16 | Additional analyses | Describe methods of additional analyses (e.g., sensitivity or subgroup analyses, meta-regression), if done, indicating which were pre-specified. | 14, 20, Fig.S6, Table S23 | Lines 253-257 “In addition, given the heterogeneity of study features, their inconsistent reporting across studies, and the small number of studies limiting power to detect associations, we descriptively synthesised features potentially associated with “high agreement” studies (i.e. where >70% of wastewater and human AMR prevalence estimate comparisons were within ±10% of each other)”  Table S23  “Primary care was removed from testing due to the uncertainty to which this category belongs in respect to other sample types. Sensitivity analysis including primary care showed no significant difference (p=0.363)”  Lines 357-359  “. Sensitivity analysis using data from low bias studies only (n=5; 31 comparisons) showed a slight decrease in overall concordance (CCC=0.81 [95% CI 0.65-0.9]; 95% CI overlaps with that of the full phenotypic dataset described above) (Fig.S6).” |
| **Results** | | | | |
| 17 | Study selection | Give numbers of studies screened, assessed for eligibility, and included in the review, with reasons for exclusions at each stage, illustrated with a PRISMA flow diagram. | 15, Fig.1, Fig.S1 | Lines 249-251  “Of 8,867 de-duplicated studies identified using our search strategy, full-text methods for 441 relevant studies were reviewed, and based on pre-specified inclusion criteria (see Methods), 33 studies were included in the review (Fig.1 and Fig.S1).” Cont. |
| 18 | Study characteristics | For each study, present in a summary table the characteristics for which data were extracted (e.g., study size, PICOS/PECOS, follow-up period) and provide the citations. | Supplementary dataset 6 and 7 | Line 315 “for full study descriptions, see Supplementary datasets 6 and 7).” |
| 19 | Risk of bias within studies | Present data on risk of bias of each study and, if available, any outcome level assessment (see item 12). | 16-17, Supplementary dataset 5 | Lines 272-300  “Based on our modified bias domains (Table S2), 19/33 studies were judged to have overall high-risk of bias, 7/33 with an unclear-risk and 7/33 with low-risk (Supplementary dataset 5).” Cont. |
| 20 | Results of individual studies | For all outcomes considered (benefits or harms), present, for each study: (a) simple summary data for each intervention group (b) effect estimates and confidence intervals, ideally with a forest plot (unless such a plot would be misleading) | Appendix | “Table S23: Univariable logistic regression of study features associated with high-agreement studies.”  “Table S25: Counts of study features according to overall study agreement presented as harvest plots in Fig.4 and Fig.S21.” |
| 21 | Synthesis of results | Present results of each meta-analysis done, including confidence intervals and measures of consistency. | 19-26 | Lines 338-486  Sections: 3.2.1 Phenotypic wastewater-human AMR concordance  3.2.2 Genotypic wastewater-human AMR concordance  3.3.1 Logistic regression of study features and wastewater-human AMR agreement  3.3.2 Study features descriptive synthesis |
| 22 | Risk of bias across studies | Present results of any assessment of risk of bias across studies (see Item 15). | 16-17, Table S4 | Lines 272-300  “Based on our certainty assessment, we rated the overall quality of included bodies of evidence as “low to moderate” regarding the outcome of identifying concordance between wastewater and human AMR prevalence estimates (Table S4).” Cont. |
| 23 | Additional analysis | Give results of additional analyses, if done (e.g., sensitivity or subgroup analyses, meta-regression [see Item 16]). | 20,24, 25-27, Supplementary dataset 8, Fig.S6, Table S25 | Lines 446-505  Sections  3.2.4 Study features potentially associated with higher wastewater-human AMR agreement.  3.3 Studies without extractable data |
| **Discussion** | | | | |
| 24 | Summary of evidence | Summarize the main findings including the strength of evidence for each main outcome; consider their relevance to key groups (e.g., researchers, users, and policy makers). | 27-31 | Lines 508-593 “From our review and synthesis of the available data, we found characterisation of AMR in wastewater shows promise in reflecting AMR in human populations, irrespective of diverse target species, target resistances and study locations, although associations may be stronger for some species and AMR mechanisms than others, and may vary by setting and over time. The strength of this relationship varied across studies and was likely influenced by study features (e.g. design, setting, spatiotemporal sampling strategies) and AMRAMR detection method (i.e. genotypic/phenotypic); the heterogeneity of methodological approaches and lack of clear reporting of key study features made any quantitative synthesis very difficult.” Cont. |
| 25 | Limitations | Discuss limitations at study and outcome level (e.g., risk of bias), and at review-level (e.g., incomplete retrieval of identified research, reporting bias). | 31-33 | Lines 596-654 Sections  4.3 Recommendations regarding risk of bias  4.4 Limitations |
| 26 | Conclusions | Provide a general interpretation of the results in the context of other evidence, and implications for future research. | 33 | Lines 657-669 Section  5. Conclusion |
| **Funding** | | | | |
| 27 | Funding | Describe sources of funding for the systematic review and other support (e.g., supply of data); role of funders for the systematic review. | 35 | Lines 677-687 Section  Funding |

*Environment International* modified PRISMA report adapted from: Moher D, Liberati A, Tetzlaff J, Altman DG, The PRISMA Group (2009). Preferred Reporting Items for Systematic Reviews and Meta-Analyses: The PRISMA Statement. PLoS Med 6(7): e1000097. doi:10.1371/journal.pmed1000097. (Changes are minor, with text edits to accommodate the subject matter of the journal and formatting to fit page.)
