# Supplementary material for: Systematic review of wastewater surveillance of antimicrobial resistance in human populations
Source: Environ Int. 2022 Apr;162:107171. doi: 10.1016/j.envint.2022.107171 (PMC8960996; doi:10.1016/j.envint.2022.107171)
Supplement: Supplementary data 3 [file mmc3.docx]

**Full search strings**

*Database: Medline (Ovid MEDLINE® Epub Ahead of Print, In-Process & Other Non-Indexed Citations, Ovid MEDLINE® Daily and Ovid MEDLINE®) 1946 to present*

*Search Strategy:*

--------------------------------------------------------------------------------

1 exp Drug Resistance, Bacterial/

2 (AMR or ESBL or VRE or CPE or CRE or CRO or MDR or KPC).ti,ab.

3 (anti-bacterial* adj3 (resistan* or susceptib* or sensitiv* or tolerance*)).ti,ab.

4 (anti-biotic* adj3 (resistan* or susceptib* or sensitiv* or tolerance*)).ti,ab.

5 (anti-microbial* adj3 (resistan* or susceptib* or sensitiv* or tolerance*)).ti,ab.

6 (antibacterial* adj3 (resistan* or susceptib* or sensitiv* or tolerance*)).ti,ab.

7 (antibiotic* adj3 (resistan* or susceptib* or sensitiv* or tolerance*)).ti,ab.

8 (antimicrobial* adj3 (resistan* or susceptib* or sensitiv* or tolerance*)).ti,ab.

9 (bacteria* adj3 (resistan* or susceptib* or sensitiv* or tolerance*)).ti,ab.

10 (("multi-drug" or drug) adj3 (resistan* or susceptib* or sensitiv* or tolerance*)).ti,ab.

11 (resistan* adj3 (trend* or distribution* or pattern* or "community-level" or "population-level")).ti,ab.

12 (resistan* adj3 (detect* or monitor* or report* or surveillan*)).ti,ab.

13 1 or 2 or 3 or 4 or 5 or 6 or 7 or 8 or 9 or 10 or 11 or 12

Annotation: AMR concept

14 exp waste water/

15 exp Environmental Monitoring/

16 Waste Disposal, Fluid/

17 Medical Waste Disposal/

18 (sewer* or sewershed* or watershed* or "septic tank" or septage).ti,ab.

19 (wastewater* or "waste-water*" or sewage* or influent* or effluent* or "untreated waste*" or "raw waste*" or "raw inflow*" or "untreated inflow*").ti,ab.

20 ("water-treatment" or "sewage treatment work*" or STW* or "wastewater treatment plant*" or WWTP* or "sewage treatment plant*" or STP* or WWTW* or "wastewater treatment work*" or "waste-water treatment plant*" or "waste-water treatment work*").ti,ab.

21 (water* adj3 (sample* or specimen*)).ti,ab.

22 14 or 15 or 16 or 17 or 18 or 19 or 20 or 21

Annotation: wastewater concept

23 exp Bacteriological Techniques/

24 exp Population Surveillance/

25 whole genome sequencing/ or Metagenomics/ or high-throughput nucleotide sequencing/

26 sequence alignment/ or phylogeny/ or molecular epidemiology/

27 Water Microbiology/

28 Drug Prescriptions/

29 Tandem Mass Spectrometry/ or Chromatography, Liquid/

30 cross-sectional studies/ or Longitudinal Studies/

31 epidemiologic research design/ or epidemiological monitoring/

32 (longitudinal or "cross-sectional" or spatial or geospatial or quantitative evaluation).ti,ab.

33 ("antibiotic-residue*" or "antibiotic-compound*").ti,ab.

34 ((antibiotic or anti-biotic or antibacterial or anti-bacterial or antimicrobial or anti-microbial) adj3 (quantit* or concentration* or use* or usage*)).ti,ab.

35 ("disc-diffusion" or "selective media*" or "antimicrobial susceptibility testing" or AST or "chromogenic" or PCR or "polymerase chain reaction" or "PFGE pulsed field gel electrophoresis" or "Multilocus sequence typing" or MLST).ti,ab.

36 (autosampler or "24-h composite").ti,ab.

37 chemical analys*.ti,ab.

38 ("liquid chromotography" or "tandem mass spectrometry" or "LC-MS/MS" or "solid-phase extraction*" or "liquid chromatography-tandem mass spectrometry").ti,ab.

39 (phylogen* or "sequence analys*" or "high-throughput nucleotide sequencing" or "DNA sequen*" or "whole-genome sequenc*" or WGS* or "HiSeq" or "molecular-epidemiolog*" or metagenom* or "Hi-C").ti,ab.

40 (surveillance or "early warning*" or epidemiolog* or monitor* or "public health").ti,ab.

41 ((antibiotic or anti-biotic or antibacterial or anti-bacterial or antimicrobial or anti-microbial) adj3 prescri*).ti,ab.

42 ((associat* or compar*) adj3 (genotyp* or prescri* or "DNA sequen*" or trend* or distribution*)).ti,ab.

43 (local* adj3 (clinic* or hospital* or isolate* or strain* or communit*)).ti,ab.

44 ((associat* or compar*) adj3 (time* or location* or geographic* or region* or communit*)).ti,ab.

45 (relat* adj3 (isolate* or strain* or cluster*)).ti,ab.

46 ((epidemiolog* or environment* or sentinel or domestic or communit* or population or "public health") adj3 (surveillance or monitor* or report* or detect*)).ti,ab.

47 ("community-level" adj3 (trend* or distribution* or detect* or monitor* or report* or surveillan* or epidemiolog*)).ti,ab.

48 ("population-level" adj3 (trend* or distribution* or detect* or monitor* or report* or surveillan* or epidemiolog*)).ti,ab.

49 23 or 24 or 25 or 26 or 27 or 28 or 29 or 30 or 31 or 32 or 33 or 34 or 35 or 37 or 38 or 39 or 40 or 41 or 42 or 43 or 44 or 45 or 46 or 47 or 48

Annotation: methods / surveillance

50 exp Bacteria/

51 ("escherichia coli" or "e. coli" or "e.coli" or "e coli" or klebsiella* or citrobacter* or enterobacter* or gram-negative*).ti,ab.

52 50 or 51

Annotation: Bacteria concept

53 13 and 22 and 49 and 52

Annotation: Combined concepts

54 limit 53 to english language

***************************

*Database: Embase 1974 to present*

*Search Strategy:*

--------------------------------------------------------------------------------

1 exp antibiotic resistance/

2 (AMR or ESBL or VRE or CPE or CRE or CRO or MDR or KPC).ti,ab.

3 (anti-bacterial* adj3 (resistan* or susceptib* or sensitiv* or tolerance*)).ti,ab.

4 (anti-biotic* adj3 (resistan* or susceptib* or sensitiv* or tolerance*)).ti,ab.

5 (anti-microbial* adj3 (resistan* or susceptib* or sensitiv* or tolerance*)).ti,ab.

6 (antibacterial* adj3 (resistan* or susceptib* or sensitiv* or tolerance*)).ti,ab.

7 (antibiotic* adj3 (resistan* or susceptib* or sensitiv* or tolerance*)).ti,ab.

8 (antimicrobial* adj3 (resistan* or susceptib* or sensitiv* or tolerance*)).ti,ab.

9 (bacteria* adj3 (resistan* or susceptib* or sensitiv* or tolerance*)).ti,ab.

10 (("multi-drug" or drug) adj3 (resistan* or susceptib* or sensitiv* or tolerance*)).ti,ab.

11 (resistan* adj3 (trend* or distribution* or pattern* or "community-level" or "population-level")).ti,ab.

12 (resistan* adj3 (detect* or monitor* or report* or surveillan*)).ti,ab.

13 1 or 2 or 3 or 4 or 5 or 6 or 7 or 8 or 9 or 10 or 11 or 12

14 exp waste water/

15 exp Environmental Monitoring/

16 Waste Disposal, Fluid/

17 Medical Waste Disposal/

18 (sewer* or sewershed* or watershed* or "septic tank" or septage).ti,ab.

19 (wastewater* or "waste-water*" or sewage* or influent* or effluent* or "untreated waste*" or "raw waste*" or "raw inflow*" or "untreated inflow*").ti,ab.

20 ("water-treatment" or "sewage treatment work*" or STW* or "wastewater treatment plant*" or WWTP* or "sewage treatment plant*" or STP* or WWTW* or "wastewater treatment work*" or "waste-water treatment plant*" or "waste-water treatment work*").ti,ab.

21 (water* adj3 (sample* or specimen*)).ti,ab.

22 14 or 15 or 16 or 17 or 18 or 19 or 20 or 21

23 exp Bacteriological Techniques/

24 exp Population Surveillance/

25 whole genome sequencing/ or Metagenomics/ or high-throughput nucleotide sequencing/

26 sequence alignment/ or phylogeny/ or molecular epidemiology/

27 Water Microbiology/

28 Drug Prescriptions/

29 Tandem Mass Spectrometry/ or Chromatography, Liquid/

30 cross-sectional studies/ or Longitudinal Studies/

31 epidemiologic research design/ or epidemiological monitoring/

32 (longitudinal or "cross-sectional" or spatial or geospatial or quantitative evaluation).ti,ab.

33 ("antibiotic-residue*" or "antibiotic-compound*").ti,ab.

34 ((antibiotic or anti-biotic or antibacterial or anti-bacterial or antimicrobial or anti-microbial) adj3 (quantit* or concentration* or use* or usage*)).ti,ab.

35 ("disc-diffusion" or "selective media*" or "antimicrobial susceptibility testing" or AST or "chromogenic" or PCR or "polymerase chain reaction" or "PFGE pulsed field gel electrophoresis" or "Multilocus sequence typing" or MLST).ti,ab.

36 (autosampler or "24-h composite").ti,ab.

37 chemical analys*.ti,ab.

38 ("liquid chromotography" or "tandem mass spectrometry" or "LC-MS/MS" or "solid-phase extraction*" or "liquid chromatography-tandem mass spectrometry").ti,ab.

39 (phylogen* or "sequence analys*" or "high-throughput nucleotide sequencing" or "DNA sequen*" or "whole-genome sequenc*" or WGS* or "HiSeq" or "molecular-epidemiolog*" or metagenom* or "Hi-C").ti,ab.

40 (surveillance or "early warning*" or epidemiolog* or monitor* or "public health").ti,ab.

41 ((antibiotic or anti-biotic or antibacterial or anti-bacterial or antimicrobial or anti-microbial) adj3 prescri*).ti,ab.

42 ((associat* or compar*) adj3 (genotyp* or prescri* or "DNA sequen*" or trend* or distribution*)).ti,ab.

43 (local* adj3 (clinic* or hospital* or isolate* or strain* or communit*)).ti,ab.

44 ((associat* or compar*) adj3 (time* or location* or geographic* or region* or communit*)).ti,ab.

45 (relat* adj3 (isolate* or strain* or cluster*)).ti,ab.

46 ((epidemiolog* or environment* or sentinel or domestic or communit* or population or "public health") adj3 (surveillance or monitor* or report* or detect*)).ti,ab.

47 ("community-level" adj3 (trend* or distribution* or detect* or monitor* or report* or surveillan* or epidemiolog*)).ti,ab.

48 ("population-level" adj3 (trend* or distribution* or detect* or monitor* or report* or surveillan* or epidemiolog*)).ti,ab.

49 23 or 24 or 25 or 26 or 27 or 28 or 29 or 30 or 31 or 32 or 33 or 34 or 35 or 37 or 38 or 39 or 40 or 41 or 42 or 43 or 44 or 45 or 46 or 47 or 48

50 exp Bacteria/

51 ("escherichia coli" or "e. coli" or "e.coli" or "e coli" or klebsiella* or citrobacter* or enterobacter* or gram-negative*).ti,ab.

52 50 or 51 (1532136)

53 13 and 22 and 49 and 52

54 limit 53 to english language

***************************

*Database: Global Health <1973 to 2019 Week 04>*

*Search Strategy:*

--------------------------------------------------------------------------------

1 exp drug resistance/

2 (AMR or ESBL or VRE or CPE or CRE or CRO or MDR or KPC).ti,ab.

3 (anti-bacterial* adj3 (resistan* or susceptib* or sensitiv* or tolerance*)).ti,ab.

4 (anti-biotic* adj3 (resistan* or susceptib* or sensitiv* or tolerance*)).ti,ab.

5 (anti-microbial* adj3 (resistan* or susceptib* or sensitiv* or tolerance*)).ti,ab.

6 (antibacterial* adj3 (resistan* or susceptib* or sensitiv* or tolerance*)).ti,ab.

7 (antibiotic* adj3 (resistan* or susceptib* or sensitiv* or tolerance*)).ti,ab.

8 (antimicrobial* adj3 (resistan* or susceptib* or sensitiv* or tolerance*)).ti,ab.

9 (bacteria* adj3 (resistan* or susceptib* or sensitiv* or tolerance*)).ti,ab.

10 (("multi-drug" or drug) adj3 (resistan* or susceptib* or sensitiv* or tolerance*)).ti,ab.

11 (resistan* adj3 (trend* or distribution* or pattern* or "community-level" or "population-level")).ti,ab.

12 (resistan* adj3 (detect* or monitor* or report* or surveillan*)).ti,ab.

13 1 or 2 or 3 or 4 or 5 or 6 or 7 or 8 or 9 or 10 or 11 or 12

14 exp waste water/

15 monitoring/

16 wastewater treatment/

17 exp wastewater treatment plants/

18 (sewer* or sewershed* or watershed* or "septic tank" or septage).ti,ab.

19 (wastewater* or "waste-water*" or sewage* or influent* or effluent* or "untreated waste*" or "raw waste*" or "raw inflow*" or "untreated inflow*").ti,ab.

20 ("water-treatment" or "sewage treatment work*" or STW* or "wastewater treatment plant*" or WWTP* or "sewage treatment plant*" or STP* or WWTW* or "wastewater treatment work*" or "waste-water treatment plant*" or "waste-water treatment work*").ti,ab.

21 (water* adj3 (sample* or specimen*)).ti,ab.

22 14 or 15 or 16 or 17 or 18 or 19 or 20 or 21

23 bacteriology/

24 exp surveillance/

25 exp DNA sequencing/

26 phylogeny/ or molecular epidemiology/

27 Water Microbiology/

28 prescriptions/

29 Mass Spectrometry/ or Liquid chromatography/

30 Longitudinal Studies/

31 disease prevalence/ or disease distribution/

32 (longitudinal or "cross-sectional" or spatial or geospatial or quantitative evaluation).ti,ab.

33 ("antibiotic-residue*" or "antibiotic-compound*").ti,ab.

34 ((antibiotic or anti-biotic or antibacterial or anti-bacterial or antimicrobial or anti-microbial) adj3 (quantit* or concentration* or use* or usage*)).ti,ab.

35 ("disc-diffusion" or "selective media*" or "antimicrobial susceptibility testing" or AST or "chromogenic" or PCR or "polymerase chain reaction" or "PFGE pulsed field gel electrophoresis" or "Multilocus sequence typing" or MLST).ti,ab.

36 (autosampler or "24-h composite").ti,ab.

37 chemical analys*.ti,ab.

38 ("liquid chromotography" or "tandem mass spectrometry" or "LC-MS/MS" or "solid-phase extraction*" or "liquid chromatography-tandem mass spectrometry").ti,ab.

39 (phylogen* or "sequence analys*" or "high-throughput nucleotide sequencing" or "DNA sequen*" or "whole-genome sequenc*" or WGS* or "HiSeq" or "molecular-epidemiolog*" or metagenom* or "Hi-C").ti,ab.

40 (surveillance or "early warning*" or epidemiolog* or monitor* or "public health").ti,ab.

41 ((antibiotic or anti-biotic or antibacterial or anti-bacterial or antimicrobial or anti-microbial) adj3 prescri*).ti,ab.

42 ((associat* or compar*) adj3 (genotyp* or prescri* or "DNA sequen*" or trend* or distribution*)).ti,ab.

43 (local* adj3 (clinic* or hospital* or isolate* or strain* or communit*)).ti,ab.

44 ((associat* or compar*) adj3 (time* or location* or geographic* or region* or communit*)).ti,ab.

45 (relat* adj3 (isolate* or strain* or cluster*)).ti,ab.

46 ((epidemiolog* or environment* or sentinel or domestic or communit* or population or "public health") adj3 (surveillance or monitor* or report* or detect*)).ti,ab.

47 ("community-level" adj3 (trend* or distribution* or detect* or monitor* or report* or surveillan* or epidemiolog*)).ti,ab.

48 ("population-level" adj3 (trend* or distribution* or detect* or monitor* or report* or surveillan* or epidemiolog*)).ti,ab.

49 23 or 24 or 25 or 26 or 27 or 28 or 29 or 30 or 31 or 32 or 33 or 34 or 35 or 37 or 38 or 39 or 40 or 41 or 42 or 43 or 44 or 45 or 46 or 47 or 48

50 exp Bacteria/

51 ("escherichia coli" or "e. coli" or "e.coli" or "e coli" or klebsiella* or citrobacter* or enterobacter* or gram-negative*).ti,ab.

52 50 or 51

53 13 and 22 and 49 and 52

54 limit 53 to english language

***************************

*Database: CAB Abstracts <1973 to 2019 Week 04>*

*Search Strategy:*

--------------------------------------------------------------------------------

1 exp drug resistance/ (54946)

2 (AMR or ESBL or VRE or CPE or CRE or CRO or MDR or KPC).ti,ab.

3 (anti-bacterial* adj3 (resistan* or susceptib* or sensitiv* or tolerance*)).ti,ab.

4 (anti-biotic* adj3 (resistan* or susceptib* or sensitiv* or tolerance*)).ti,ab.

5 (anti-microbial* adj3 (resistan* or susceptib* or sensitiv* or tolerance*)).ti,ab.

6 (antibacterial* adj3 (resistan* or susceptib* or sensitiv* or tolerance*)).ti,ab.

7 (antibiotic* adj3 (resistan* or susceptib* or sensitiv* or tolerance*)).ti,ab.

8 (antimicrobial* adj3 (resistan* or susceptib* or sensitiv* or tolerance*)).ti,ab.

9 (bacteria* adj3 (resistan* or susceptib* or sensitiv* or tolerance*)).ti,ab.

10 (("multi-drug" or drug) adj3 (resistan* or susceptib* or sensitiv* or tolerance*)).ti,ab.

11 (resistan* adj3 (trend* or distribution* or pattern* or "community-level" or "population-level")).ti,ab.

12 (resistan* adj3 (detect* or monitor* or report* or surveillan*)).ti,ab.

13 1 or 2 or 3 or 4 or 5 or 6 or 7 or 8 or 9 or 10 or 11 or 12

14 exp waste water/

15 monitoring/

16 wastewater treatment/

17 exp wastewater treatment plants/

18 (sewer* or sewershed* or watershed* or "septic tank" or septage).ti,ab.

19 (wastewater* or "waste-water*" or sewage* or influent* or effluent* or "untreated waste*" or "raw waste*" or "raw inflow*" or "untreated inflow*").ti,ab.

20 ("water-treatment" or "sewage treatment work*" or STW* or "wastewater treatment plant*" or WWTP* or "sewage treatment plant*" or STP* or WWTW* or "wastewater treatment work*" or "waste-water treatment plant*" or "waste-water treatment work*").ti,ab.

21 (water* adj3 (sample* or specimen*)).ti,ab.

22 14 or 15 or 16 or 17 or 18 or 19 or 20 or 21

23 bacteriology/

24 exp surveillance/

25 exp DNA sequencing/

26 phylogeny/ or molecular epidemiology/

27 Water Microbiology/

28 prescriptions/

29 Mass Spectrometry/ or Liquid chromatography/

30 Longitudinal Studies/

31 disease prevalence/ or disease distribution/

32 (longitudinal or "cross-sectional" or spatial or geospatial or quantitative evaluation).ti,ab.

33 ("antibiotic-residue*" or "antibiotic-compound*").ti,ab.

34 ((antibiotic or anti-biotic or antibacterial or anti-bacterial or antimicrobial or anti-microbial) adj3 (quantit* or concentration* or use* or usage*)).ti,ab.

35 ("disc-diffusion" or "selective media*" or "antimicrobial susceptibility testing" or AST or "chromogenic" or PCR or "polymerase chain reaction" or "PFGE pulsed field gel electrophoresis" or "Multilocus sequence typing" or MLST).ti,ab.

36 (autosampler or "24-h composite").ti,ab.

37 chemical analys*.ti,ab.

38 ("liquid chromotography" or "tandem mass spectrometry" or "LC-MS/MS" or "solid-phase extraction*" or "liquid chromatography-tandem mass spectrometry").ti,ab.

39 (phylogen* or "sequence analys*" or "high-throughput nucleotide sequencing" or "DNA sequen*" or "whole-genome sequenc*" or WGS* or "HiSeq" or "molecular-epidemiolog*" or metagenom* or "Hi-C").ti,ab.

40 (surveillance or "early warning*" or epidemiolog* or monitor* or "public health").ti,ab.

41 ((antibiotic or anti-biotic or antibacterial or anti-bacterial or antimicrobial or anti-microbial) adj3 prescri*).ti,ab.

42 ((associat* or compar*) adj3 (genotyp* or prescri* or "DNA sequen*" or trend* or distribution*)).ti,ab.

43 (local* adj3 (clinic* or hospital* or isolate* or strain* or communit*)).ti,ab.

44 ((associat* or compar*) adj3 (time* or location* or geographic* or region* or communit*)).ti,ab.

45 (relat* adj3 (isolate* or strain* or cluster*)).ti,ab.

46 ((epidemiolog* or environment* or sentinel or domestic or communit* or population or "public health") adj3 (surveillance or monitor* or report* or detect*)).ti,ab.

47 ("community-level" adj3 (trend* or distribution* or detect* or monitor* or report* or surveillan* or epidemiolog*)).ti,ab.

48 ("population-level" adj3 (trend* or distribution* or detect* or monitor* or report* or surveillan* or epidemiolog*)).ti,ab.

49 23 or 24 or 25 or 26 or 27 or 28 or 29 or 30 or 31 or 32 or 33 or 34 or 35 or 37 or 38 or 39 or 40 or 41 or 42 or 43 or 44 or 45 or 46 or 47 or 48

50 exp Bacteria/

51 ("escherichia coli" or "e. coli" or "e.coli" or "e coli" or klebsiella* or citrobacter* or enterobacter* or gram-negative*).ti,ab.

52 50 or 51

53 13 and 22 and 49 and 52

54 limit 53 to english language

***************************

*Database: Scopus*

*Search Strategy:*

--------------------------------------------------------------------------------

( ( TITLE-ABS-KEY ( amr OR esbl OR vre OR cpe OR cre OR cro OR mdr OR kpc ) ) OR ( TITLE-ABS-KEY ( anti-bacterial* W/3 ( resistan* OR susceptib* OR sensitiv* OR tolerance* ) ) ) OR ( TITLE-ABS-KEY ( anti-biotic* W/3 ( resistan* OR susceptib* OR sensitiv* OR tolerance* ) ) ) OR ( TITLE-ABS-KEY ( anti-microbial* W/3 ( resistan* OR susceptib* OR sensitiv* OR tolerance* ) ) ) OR ( TITLE-ABS-KEY ( antibacterial* W/3 ( resistan* OR susceptib* OR sensitiv* OR tolerance* ) ) ) OR ( TITLE-ABS-KEY ( antibiotic* W/3 ( resistan* OR susceptib* OR sensitiv* OR tolerance* ) ) ) OR ( TITLE-ABS-KEY ( antimicrobial* W/3 ( resistan* OR susceptib* OR sensitiv* OR tolerance* ) ) ) OR ( TITLE-ABS-KEY ( bacteria* W/3 ( resistan* OR susceptib* OR sensitiv* OR tolerance* ) ) ) OR ( TITLE-ABS-KEY ( ( "multi-drug" OR drug ) W/3 ( resistan* OR susceptib* OR sensitiv* OR tolerance* ) ) ) OR ( TITLE-ABS-KEY ( resistan* W/3 ( trend* OR distribution* OR pattern* OR "community-level" OR "population-level" ) ) ) OR ( TITLE-ABS-KEY ( resistan* W/3 ( detect* OR monitor* OR report* OR surveillan* ) ) ) ) AND ( ( TITLE-ABS-KEY ( sewer* OR sewershed* OR watershed* OR "septic tank" OR septage OR wastewater* OR "waste-water*" OR sewage* OR influent* OR effluent* OR "untreated waste*" OR "raw waste*" OR "raw inflow*" OR "untreated inflow*" OR "water-treatment" ) ) OR ( TITLE-ABS-KEY ( "sewage treatment work*" OR stw* OR "wastewater treatment plant*" OR wwtp* OR "sewage treatment plant*" OR stp* OR wwtw* OR "wastewater treatment work*" OR "waste-water treatment plant*" OR "waste-water treatment work*" ) ) OR ( TITLE-ABS-KEY ( water* W/3 ( sample* OR specimen* ) ) ) ) AND ( ( TITLE-ABS-KEY ( longitudinal OR "cross-sectional" OR spatial OR geospatial OR quantitative AND evaluation OR "antibiotic-residue*" OR "antibiotic-compound*" ) ) OR ( TITLE-ABS-KEY ( ( ( antibiotic OR anti-biotic OR antibacterial OR anti-bacterial OR antimicrobial OR anti-microbial ) W/3 ( quantit* OR concentration* OR use* OR usage* ) ) ) ) OR ( TITLE-ABS-KEY ( "disc-diffusion" OR "selective media*" OR "antimicrobial susceptibility testing" OR ast OR "chromogenic" OR pcr OR "polymerase chain reaction" OR "PFGE pulsed field gel electrophoresis" OR "Multilocus sequence typing" OR mlst ) ) OR ( TITLE-ABS-KEY ( autosampler OR "24-h composite" OR "chemical analys*" OR "liquid chromotography" OR "tandem mass spectrometry" OR "LC-MS/MS" OR "solid-phase extraction*" OR "liquid chromatography-tandem mass spectrometry" ) ) OR ( TITLE-ABS-KEY ( phylogen* OR "sequence analys*" OR "high-throughput nucleotide sequencing" OR "DNA sequen*" OR "whole-genome sequenc*" OR wgs* OR "HiSeq" OR "molecular-epidemiolog*" OR metagenom* OR "Hi-C" ) ) OR ( TITLE-ABS-KEY ( surveillance OR "early warning*" OR epidemiolog* OR monitor* OR "public health" ) ) OR ( TITLE-ABS-KEY ( ( ( antibiotic OR anti-biotic OR antibacterial OR anti-bacterial OR antimicrobial OR anti-microbial ) W/3 prescri* ) ) ) OR ( TITLE-ABS-KEY ( ( ( associat* OR compar* ) W/3 ( genotyp* OR prescri* OR "DNA sequen*" OR trend* OR distribution* ) ) ) ) OR ( TITLE-ABS-KEY ( ( local* W/3 ( clinic* OR hospital* OR isolate* OR strain* OR communit* ) ) ) ) OR ( TITLE-ABS-KEY ( ( ( associat* OR compar* ) W/3 ( time* OR location* OR geographic* OR region* OR communit* ) ) ) ) OR ( TITLE-ABS-KEY ( ( relat* W/3 ( isolate* OR strain* OR cluster* ) ) ) ) OR ( TITLE-ABS-KEY ( ( ( epidemiolog* OR environment* OR sentinel OR domestic OR communit* OR population OR "public health" ) W/3 ( surveillance OR monitor* OR report* OR detect* ) ) ) ) OR ( TITLE-ABS-KEY ( ( "community-level" W/3 ( trend* OR distribution* OR detect* OR monitor* OR report* OR surveillan* OR epidemiolog* ) ) ) ) OR ( TITLE-ABS-KEY ( ( "population-level" W/3 ( trend* OR distribution* OR detect* OR monitor* OR report* OR surveillan* OR epidemiolog* ) ) ) ) ) AND ( TITLE-ABS-KEY ( ( bacteri* OR gram-positive* OR "escherichia coli" OR "e. coli" OR "e.coli" OR "e coli" OR klebsiella* OR citrobacter* OR enterobacter* OR gram-negative* ) ) ) AND ( LIMIT-TO ( LANGUAGE , "English" ) )

***************************

*Database: Web of Science*

*Search Strategy:*

--------------------------------------------------------------------------------

( ( TITLE-ABS-KEY ( amr OR esbl OR vre OR cpe OR cre OR cro OR mdr OR kpc ) ) OR ( TITLE-ABS-KEY ( anti-bacterial* near/3 ( resistan* OR susceptib* OR sensitiv* OR tolerance* ) ) ) OR ( TITLE-ABS-KEY ( anti-biotic* near/3 ( resistan* OR susceptib* OR sensitiv* OR tolerance* ) ) ) OR ( TITLE-ABS-KEY ( anti-microbial* near/3 ( resistan* OR susceptib* OR sensitiv* OR tolerance* ) ) ) OR ( TITLE-ABS-KEY ( antibacterial* near/3 ( resistan* OR susceptib* OR sensitiv* OR tolerance* ) ) ) OR ( TITLE-ABS-KEY ( antibiotic* near/3 ( resistan* OR susceptib* OR sensitiv* OR tolerance* ) ) ) OR ( TITLE-ABS-KEY ( antimicrobial* near/3 ( resistan* OR susceptib* OR sensitiv* OR tolerance* ) ) ) OR ( TITLE-ABS-KEY ( bacteria* near/3 ( resistan* OR susceptib* OR sensitiv* OR tolerance* ) ) ) OR ( TITLE-ABS-KEY ( ( "multi-drug" OR drug ) near/3 ( resistan* OR susceptib* OR sensitiv* OR tolerance* ) ) ) OR ( TITLE-ABS-KEY ( resistan* near/3 ( trend* OR distribution* OR pattern* OR "community-level" OR "population-level" ) ) ) OR ( TITLE-ABS-KEY ( resistan* near/3 ( detect* OR monitor* OR report* OR surveillan* ) ) ) ) AND ( ( TITLE-ABS-KEY ( sewer* OR sewershed* OR watershed* OR "septic tank" OR septage OR wastewater* OR "waste-water*" OR sewage* OR influent* OR effluent* OR "untreated waste*" OR "raw waste*" OR "raw inflow*" OR "untreated inflow*" OR "water-treatment" ) ) OR ( TITLE-ABS-KEY ( "sewage treatment work*" OR stw* OR "wastewater treatment plant*" OR wwtp* OR "sewage treatment plant*" OR stp* OR wwtw* OR "wastewater treatment work*" OR "waste-water treatment plant*" OR "waste-water treatment work*" ) ) OR ( TITLE-ABS-KEY ( water* near/3 ( sample* OR specimen* ) ) ) ) AND ( ( TITLE-ABS-KEY ( longitudinal OR "cross-sectional" OR spatial OR geospatial OR quantitative AND evaluation OR "antibiotic-residue*" OR "antibiotic-compound*" ) ) OR ( TITLE-ABS-KEY ( ( ( antibiotic OR anti-biotic OR antibacterial OR anti-bacterial OR antimicrobial OR anti-microbial ) near/3 ( quantit* OR concentration* OR use* OR usage* ) ) ) ) OR ( TITLE-ABS-KEY ( "disc-diffusion" OR "selective media*" OR "antimicrobial susceptibility testing" OR ast OR "chromogenic" OR pcr OR "polymerase chain reaction" OR "PFGE pulsed field gel electrophoresis" OR "Multilocus sequence typing" OR mlst ) ) OR ( TITLE-ABS-KEY ( autosampler OR "24-h composite" OR "chemical analys*" OR "liquid chromotography" OR "tandem mass spectrometry" OR "LC-MS/MS" OR "solid-phase extraction*" OR "liquid chromatography-tandem mass spectrometry" ) ) OR ( TITLE-ABS-KEY ( phylogen* OR "sequence analys*" OR "high-throughput nucleotide sequencing" OR "DNA sequen*" OR "whole-genome sequenc*" OR wgs* OR "HiSeq" OR "molecular-epidemiolog*" OR metagenom* OR "Hi-C" ) ) OR ( TITLE-ABS-KEY ( surveillance OR "early warning*" OR epidemiolog* OR monitor* OR "public health" ) ) OR ( TITLE-ABS-KEY ( ( ( antibiotic OR anti-biotic OR antibacterial OR anti-bacterial OR antimicrobial OR anti-microbial ) near/3 prescri* ) ) ) OR ( TITLE-ABS-KEY ( ( ( associat* OR compar* ) near/3 ( genotyp* OR prescri* OR "DNA sequen*" OR trend* OR distribution* ) ) ) ) OR ( TITLE-ABS-KEY ( ( local* near/3 ( clinic* OR hospital* OR isolate* OR strain* OR communit* ) ) ) ) OR ( TITLE-ABS-KEY ( ( ( associat* OR compar* ) near/3 ( time* OR location* OR geographic* OR region* OR communit* ) ) ) ) OR ( TITLE-ABS-KEY ( ( relat* near/3 ( isolate* OR strain* OR cluster* ) ) ) ) OR ( TITLE-ABS-KEY ( ( ( epidemiolog* OR environment* OR sentinel OR domestic OR communit* OR population OR "public health" ) near/3 ( surveillance OR monitor* OR report* OR detect* ) ) ) ) OR ( TITLE-ABS-KEY ( ( "community-level" near/3 ( trend* OR distribution* OR detect* OR monitor* OR report* OR surveillan* OR epidemiolog* ) ) ) ) OR ( TITLE-ABS-KEY ( ( "population-level" near/3 ( trend* OR distribution* OR detect* OR monitor* OR report* OR surveillan* OR epidemiolog* ) ) ) ) ) AND ( TITLE-ABS-KEY ( ( bacteri* OR gram-positive* OR "escherichia coli" OR "e. coli" OR "e.coli" OR "e coli" OR klebsiella* OR citrobacter* OR enterobacter* OR gram-negative* ) ) ) AND ( LIMIT-TO ( LANGUAGE , "English" ) )

***************************
