# Supplementary material for: Systematic review of wastewater surveillance of antimicrobial resistance in human populations
Source: Environ Int. 2022 Apr;162:107171. doi: 10.1016/j.envint.2022.107171 (PMC8960996; doi:10.1016/j.envint.2022.107171)
Supplement: Supplementary data 4 [file mmc4.docx]

**
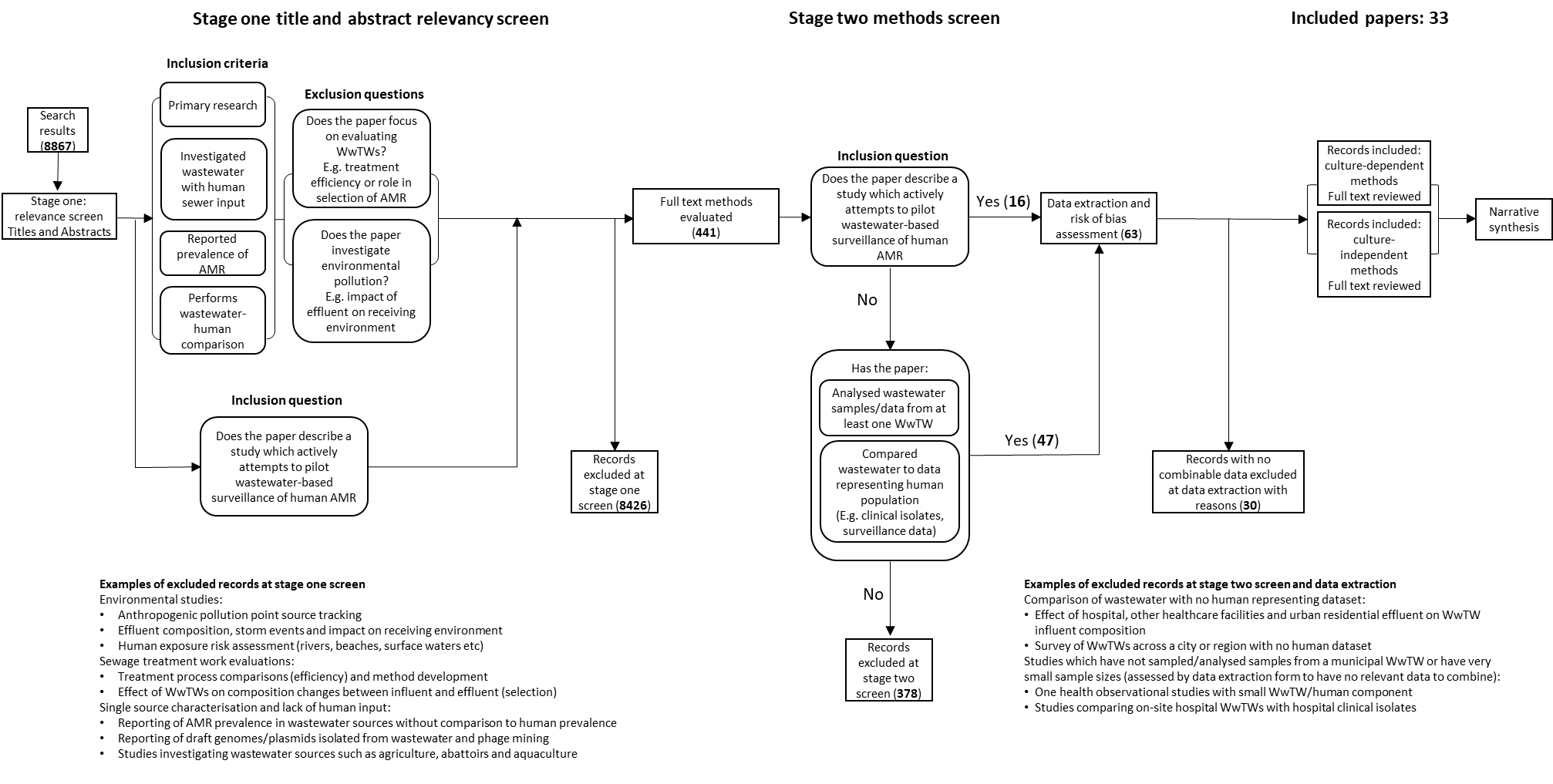
**

**Stage one – Titles and abstract relevance screening**

Only record titles and abstracts are screened

*Inclusion criteria:*

- Primary research

Record describes research actively conducted by the author/s and does not describe a synthesis or summary of previously published work, i.e. review articles. This criterion does not preclude the inclusion of records performing their own original analyses on previously published datasets such as in the use of metagenomes from collaborations or online repositories.

- Investigated wastewater including human sewer input

Record must have investigated wastewater which at least in part was constituted by human sewer inputs (e.g. municipal wastewater treatment works). As with the criterion of primary research, investigation of wastewater includes studies utilising data derived from wastewater but not produced by the record itself. Records specifically investigating wastewater without human input such as agricultural, industrial and abattoir effluents are to be excluded.

- Reported prevalence of AMR

Record must have reported prevalence of AMR as a result of the work/analyses undertaken. This can include but is not limited to counts of resistant and susceptible isolates, counts of isolates with and without specific AMR determinants, and antibiograms. Records reporting features such as virulence factors without AMR data are to be excluded.

- Performs comparative analyses

Record must perform a comparison of the wastewater compartment of the study to a separate non-wastewater compartment which potentially represents AMR prevalence in a human population. This criterion is kept intentionally broad to capture diverse study designs utilising varied human compartments – criterion is checked in detail during stage two full text methods screen.

*Exclusion questions:*

*Records answering yes to these questions may meet several inclusion criteria but are not relevant to the review question and are to be excluded.*

- Does the study solely evaluate WWTWs?

E.g. records evaluating treatment process efficiency, transformation of microbial communities during treatment, occurrence of horizontal gene transfer in WWTWs, selection of AMR in WWTWs, and WWTWs as a reservoir of AMR.

- Does the paper investigate environmental pollution?

E.g. records investigating WWTW effluents as a point-sources of AMR, river environments and surface waters impacted by wastewater, the effect of WWTW overflow events and faecal source tracking method development.

**Stage two – Design screening**

The methods section from the full text is screened in detail

*Inclusion criteria:*

- Analysed wastewater from at least one WWTW

The wastewater analysed in the record must originate from at least one conventional WWTW whereby multiple waste streams converge. This criterion screens out studies investigating waste specific WWTWs such as on-site hospital and agricultural WWTWs.

- The non-wastewater comparator compartment must represent a human population

The compartment which is being used as a comparator to wastewater AMR prevalence must directly represent a human population. Examples include clinical isolates, faecal carriage isolates, resistance network data and local resistance prevalence data.

**Stage one and two – Universal inclusion**

*Inclusion question:*

- Does the record describe a study which actively attempts to pilot wastewater-based surveillance of AMR?

Records which describe studies explicitly set up as wastewater-based surveillance studies of AMR in human populations are to be included irrespective on if they meet other criteria.
