# Supplementary material for: Systematic review of wastewater surveillance of antimicrobial resistance in human populations
Source: Environ Int. 2022 Apr;162:107171. doi: 10.1016/j.envint.2022.107171 (PMC8960996; doi:10.1016/j.envint.2022.107171)
Supplement: Supplementary data 7 [file mmc7.docx]

| **Citation** | **Country** | **Relevant comparison** | **Sampling method** | **WwTW details** | **AMR detection method** | **Human dataset** | **Wastewater sample sizes** | **Data analyses methods** | **Relevant outcomes** | **Overall risk of bias** |
| --- | --- | --- | --- | --- | --- | --- | --- | --- | --- | --- |
| Adator et al. 2020a | Canada | Comparison of E. coli antibiograms from wastewater isolates (selective and non-selective media) and human clinical isolates. | Composite of influent and effluent (1L). | Influent and effluent (post-grit tank) from 2 WwTWs – no details reported. | Selective and non selective culture - AST via disk diffusion and PCR of 8 AMR targets. | Clinical isolates from blood, urine and abdominal samples (n=25). | 12 wastewater samples and 194 wastewater isolates. | To compare wastewater and human group effect on AMR prevalence, univariate multinomial logistic regression models were used. | Wastewater and human components showed more similar phenotypic and genotypic resistance prevalence than when compared to other one health samples. Human isolates were not exclusively higher or lower in MDR or genotypic AMR testing than wastewater isolates. | Unclear |
| Adator et al. 2020b | Canada | Comparison of ESBL E.coli between wastewater and human clinical isolates. | Composite of influent and effluent (1L). | Influent and effluent (post-grit tank) from 2 WwTWs – with agriculture input. | Disk-diffusion and WGS. | Human clinical isolates randomly selected from the same period as sampling (subset for WGS = 25). | Unclear number of wastewater samples, WGS of wastewater isolates = 30. | Analyses conducted on generated pan genomes. Separate binary logistic regression used to example wastewater-human ARG prevalence differences. Maximum likelihood phylogenetic tree also generated. | Phylogenetic AST showed human clinical samples and wastewater isolates had similar resistance prevalence. Both groups had higher prevalence for certain antibiotics than the other. Genotypic analyses showed a similar outcome with genotype-phenotype agreement ranging from 93.2-100% depending on the antibiotic. | Unclear |
| Aljanaby et al. 2018 | Iraq | Antibiotic susceptibility of P. aeruginosa to 10 antibiotics compared for 60 isolates from patients with pneumonia and 60 wastewater isolates from the same city. | Single grab samples of "sewage water" from undefined sampling point. | "Purified water station" located in the same city as where clinical isolates were collected. | Disk diffusion for 10 antibiotics and ESBL kit tests. | 60 clinical isolates from sputum samples from patients with pneumonia. | 60 wastewater samples and 60 wastewater isolates analysed. | Chi-squared test. | Significantly higher prevalence of MDR in clinical isolates compared to wastewater isolates. Overall higher prevalence of individual antibiotic resistance in clinical isolates also. Non-significant difference in prevalence of ESBLs between the two compartments. | Unclear |
| Colomer-Lluch et al. 2014 | Tunisia, Spain | Abundance of several AMR genes in geographically-distant wastewater sources with differing characteristics and historical resistance backgrounds. Country-level comparisons of resistance rates - indirect. | Grab samples collected in sterile conditions. | Influent from four WwTWs with tourist facility and abattoir sewer inputs (sheep, cattle). | Samples filtered and phases extracted by QIAamp DNA Blood Mini Kit (retained bacteria) and phenol:chloroform in-house method (phage containing supernatant). qPCR of genes: blaTEM, blaCTX-M-1, blaCTX-M-9, mecA, armA, qnrA, qnrS, sul1. Eight target genes. | Country-specific historical resistance rates and specific resistance prevalence. | Unclear total wastewater samples: 26 wastewater samples underwent DNA extraction and 28 underwent viral phage fraction analyses. | None conducted. | Most common ESBL isolated reflects most reported clinical ESBL. Higher ARG density from high-antibiotics use country. | High |
| Golle et al. 2017 | Slovenia | Assessing the prevalence/variability of carbapenem-resistant Pseudomonas aeruginosa (CRPA) strains isolated from clinical isolates and STW effluent in the same region using PFGE and WGS. | Not reported | Effluent from two with distinct community and hospital inputs (1300 or 260 beds). Additional six healthcare facilities including geriatric primary care unit and psychiatric long-term care facility). Median flow m3/d 22207.5 (IQR 7928.5). | Disk diffusion (10 antibiotics). DNA extraction of subset (QIAamp DNA Mini Kit), MiSeq sequencing and identification of acquired carbapenemase genes using Resfinder. | CRPA cultured during the same sampling period from all respiratory and urine samples from hospitals, other health care facilities and GPs (n=130). | 83 CRPA isolated from effluent samples. Totals including clinical: 208 PFGE types and 112 sequenced. | None conducted | No pattern or uniformity of PFGE types observed over the year with low overlap (4 isolates) between clinical and effluent samples regardless of region. Low overlap also seen between hospitals and other clinical settings (2). Slightly higher overlap between WwTWs (6). AST-based resistance patterns: clinical isolates have higher diversity in resistance patterns but resistance to individual antibiotics is more prevalent in effluent for 6/8 reported. | Low |
| Gouliouris et al. 2019 | UK | Genomic comparison of wastewater E.faecium isolates and isolates from clinical infection preceding and during study. | Composite (two subsamples) of consecutive 500 ml samples. Hospital grab samples of 1 L. | Influent and effluent from 20 WwTWs where 10 received hospital waste and 10 did not. 15 urban and 5 rural sites. Median PE 47957.5 (IQR 127521) and flow m3/d 27043.2 (IQR 48621.6). | Selective media for AMR counts and VITEK microbroth dilution for AST. Hiseq for WGS. Phylogenetic WGS analyses with ResFinder (ARIBA) and plasmidSPAdes/BLASTN. | 187 E. faecium associated with bloodstream infection in 187 patients. 140/187 patients in hospitals served by sampled WwTWs preceding wastewater sampling period and 23/187 collected during sampling period. | 40 STW wastewater samples from which 388 E. faecium isolates sequenced. Five hospital wastewater samples from which 40 isolates sequenced. Total of 423 in data analysis after dropping 5 low quality sequences. | Maximum-likelihood phylogeny (no recombination/MGE). | ST typing show high diversity in influent and much lower in clinical samples – consistent with specific ST’s association with drug resistance. Comparing relatedness between sample groups showed minimum SNP distance between any bloodstream to wastewater isolates did not differ significantly for plants with hospital input or not. | Low |
| Haghi et al. 2019 | Iran | Prevalence and resistance patterns of Enterococci isolated from healthy humans and wastewater from the same region during the same period. | Grab samples collected in sterile bottles. | Six sites of "urban sewage waters". | Disk diffusion with seven antibiotics for ASTs. MIC by agar dilution method. | 100 healthy faecal samples with no history of diarrhoea and antibiotic therapy for at least one month. | 100 wastewater samples and 100 faecal specimens - total of 141 Enterococci isolated. 77/141 from wastewater samples and 64/141 from faeces. | None conducted | Top three single-resistances shared. Wastewater had higher prevalence of resistance to three other antibiotics, multi-drug resistance and VRE. | High |
| Hendriksen et al. 2019a | Kenya | Resistomes of wastewater compared to surveillance data and laboratory-confirmed cases of wastewater source populations. | Grab samples of 500 ml. | Untreated waste from two confluence points of drainage ditch networks with the high surface flow. Each point drains household latrine waste from a separate village: population (population density): 1845 (8.4/m2) and 3727 (8.8/m2). | DNA extracted (QIAamp Fast DNA Stool mini kit on pellet) and Viral RNA/DNA extracted (Nucleospin RNA XS kit on supernatant). Hiseq and Miseq (PE, 2 x 250bp) used to sequence pellet and supernatant extracts respectively. MGmapper used to map against genomic/viral databases and Resfinder for AMR genes. | PBIDS local surveillance system comprised of household morbidity and health care usage data contemporaneously collected through home visits. Key measures used in study: total cases of diarrhoea, fever, acute febrile illness and number of clinic visits. Stool culture also conducted. | 42 wastewater samples sequenced. | Confidence intervals calculated on mean read abundance. Weekly number of reads for specific bacteria, viruses or AMR genes surpassing upper limit defined as significant. | Increases/decreases in read abundances for bacteria/AMR genes were not reflected in the contemporaneous PBIDS data. Non-significant observed increase in read abundances of pathogens appeared to coincide with reported illness/visits to clinic. | Unclear |
| Hendriksen et al. 2019b | 60 countries sampled | Resistomes of sewage influent collected on a global scale compared to source regional antimicrobial use, and socio-economic, health, and environmental factors. | 24H flow proportional sampling or composites (3 sub samples) over a short time period (minimum 15 min). | Influent from 53 WwTWs.H15 44/53 received hospital input, 34/53 received industrial input, 17/53 received abattoir input and 14/53 received agricultural input. | DNA extracted (QIAmp Fast DNA Stool Mini Kit with twice input material and bead beating changes), libraries prepped (NEXTflex PCR-free Library Prep Kit) and sequenced (HiSeq3000 2 x 150bp paired-end). MGmapper used to align against reference sequence databases and AMR genes annotated using ResFinder. | World Bank Health, Nutrition, Population, Development indicator data sets collected between the years 2000 and 2016 for 259 countries and territories. | Samples sequenced for 80 sites (WwTW and others) - results from 79 reported. | Multilevel Poisson model including observation-level random effects and fixed effects. | Total AMR gene abundances detected across sites corresponded to existing data describing their country/continent of origin. Country-specific variables made up from World Bank’s Health, Nutrition and Population, Development indicator data sets (mostly sanitation and general health) explained up to 89% of observed resistome variation between samples regardless of diversity of AMR genes. Countries with higher HDI have significantly lower abundance of AMR genes and the number of passengers flying into a country has no effect on abundance. No significant association between temperature at collection with AMR genes abundance. No significant effect was found when investigating total usage of all antimicrobials on the abundance of AMR genes on a class-level. No significant association was found between the abundance of AMR genes on a class-level and the antimicrobial residue levels measured. | High |
| Huijbers et al. 2020 | Denmark, Finland, Norway, Sweden, Belgium, France, Germany, Greece, Italy, Spain | Wastewater-isolated E.coli AST compared against AMR surveillance network data for the country samples at a similar time period. | Time-proportional, flow-proportional, volume-proportional. | Influent from 10 large WwTWs (>500,000 PE each). | Non-selective chromogenic plating and isolation of suspected E.coli (confirmed by MALDI). Microbroth dilution AST against six antibiotics. | EARS-net data of invasive E. coli isolated from blood and CSF clinical samples extracted for countries sampled for 2016 (each country has contributed >1000 isolates to EARS-net previously). | 10 wastewater samples, 2507 E. coli isolated and underwent AST. | Resistance prevalence and 95% CIs calculated based on the binomial probability function. Chi-squared test used to assess relationship between isolate prevalence and national surveillance data. Simple linear regression used for wastewater-human relationships. | Resistance prevalence lower in wastewater E. coli compared to clinical E. coli but followed geographic trends based on the ten countries studied. Wastewater-human comparisons showed significant correlation for 2/4 antibiotic classes with higher sample sizes. | Low |
| Hutinel et al. 2019 | Sweden | Resistance rates of E.coli isolates from hospital/STW wastewater compared to E.coli isolated from hospital ward and primary care clinical samples during the same period. | 24H WwTW flow proportional sampling comprised of a minimum of 224 subsamples. 24H hospital effluent composite comprised of subsamples taken every 9 min (160 subsamples). | Influent from WwTW with PE 746,882. | Microbroth dilution of stocked isolates against 11 antibiotics. ESBL screening by multiple disk diffusion. | E. coli urine/blood isolates from patients on hospital wards connected to hospital wastewater sampling point or primary care patients from municipalities connected to the sampled WwTW (n=6270). Contemporaneous and before wastewater sampling period. | 14 wastewater samples from which 1252 E. coli were isolated. | Resistance across clinical/sewage modelled with linear regressions. | Resistance prevalence of hospital effluent, clinical samples and primary care urine isolates correlated with annual mean resistance rates. ESBLs higher in hospital effluent than WwTW influent. Clinical samples had double resistance rates of influent isolates except for one antibiotic. | Unclear |
| Jakobsen et al. 2008 | Denmark | Gen-R resistance E. coli isolates from sewage with/out hospital sewer input and from contemporaneous hospital patient UTI samples. | 24H flow proportional WwTW sampling. Composites (flow-respective mix) of hospital effluent. Composites of residential area outlet comprising 5 samples taken with 10 min interval (5 subsamples). | Influent from WwTW with 230,000 PE receiving industrial hospital (794 bed) input. | Microboth dilution for ASTs to five antibiotics. PCR detection of four genes: aac(3)-II, aac(3)-IV, ant(2″)-I, and armA. Additional PCR detection of aac(3)-I and aac(3)-III in subset. | 38 Gen-R E. coli isolates from hospital patients with UTIs during wastewater sampling period with addition month either side. | 33 wastewater samples from which 55 GEN-R E. coli isolates cultured. | None conducted | Prevalence of Gen-R E. coli similar between hospital effluent and WwTW influent - lowest in residential outlet. No sharing of PFGE types between any patient isolates and wastewater isolates. | High |
| Jorgensen et al. 2017 | Norway | Genomics of ESBL-E.coli isolated from influent compared to those isolated from UTI patients living in the STW catchment area with temporal overlap. | 24H flow proportional sampling. | Influent from one WwTW. | Vitek2 microbroth AST and E-test. PCR for blaCTX-M and DNA microarray for AMR 11 genes for isolates negative for blaCTX-M, Hiseq (150 PE) WGS and analysis using ResFinder and PlasmidFinder. | 94 patients with community acquired UTI caused by ESBL-E. coli living within catchment area of the WwTW collected before wastewater sampling. | Five wastewater samples from which 91 ESBL E. coli isolates cultured. | Univariate and multivariate analyses conducted by binary logistic regression. | Clinical isolate single and multi-drug resistance rates higher than wastewater isolates. Most prevalent ESBL gene was shared between groups and prevalence for other ESBL genes also similar except M-14 higher in wastewater. Prevalence of acquired resistance genes similar between groups but higher in urine isolates. No difference in plasmid replicon typing between groups. | High |
| Karkman et al. 2020 | 60 countries sampled (GSSP) | Resistomes of sewage influent collected on a global scale compared to local clinical surveillance data and socio-economic, health, and environmental factors. | Mixed (24H flow proportional and composites) | As described for Hendriksen (GSSP). | DNA extracted (QIAmp Fast DNA Stool Mini Kit with twice input material and bead beating changes), libraries prepped (NEXTflex PCR-free Library Prep Kit) and sequenced (HiSeq3000 2 x 150bp paired-end). MGmapper used to align against reference sequence databases and AMR genes annotated using ResFinder. | Clinical resistance data for invasive E. coli (blood, CSF) against four classes of antibiotics extracted from networks EARS-Net, CAESAR and ResistanceMap. | As described for GSSP. | Prevalence of clinical resistance was modelled using beta regression. Further analyses with PCA, correlation plots and heatmaps. | Sewage resistomes correlated to surveillance data of invasive E. coli in terms of prevalence on a global scale. Resistome ARGs were mostly insufficient to explain variability of clinical resistance. Similarly, resistance to antibiotic classes could not be associated with corresponding ARG resistance classes except for beta-lactam ARGs and aminopenicillin resistance. Combining sewage resistome data and socioeconomic data from respective countries produced the best performing models. | High |
| King et al. 2020 | South Africa | Comparison of AMR profiles of wastewater-isolated Klebsiella and clinical Klebsiella isolates | Grab sampling. | Influent and effluent from 2 WwTWs (no details reported) | Disk diffusion and AmpC/ESBL detection kits. | Undefined clinical isolates (n=72). | 4 wastewater samples and 37 Klebsiella isolated. | None conducted. | Common resistance profiles were detected between clinical and wastewater isolates. Wastewater isolates had a lower resistance prevalence compared to clinical but much fewer wastewater isolates were collected. Carbapenem resistance was detected in clinical sampled but not in WwTW samples (was detected in hospital effluent though). | Unclear |
| Meir-Gruber et al. 2016 | Israel | Prevalence of blaKPC/blaNDM-1 carrying CREs, MRSA and VRE in STW influent and domestic sewer systems collected nationally during 2012-2013 compared to in clinical samples from a major hospital during 2013. Distribution of CREs, MRSA and VRE from sampling sites with and without hospital sewage input. | 24H WwTW and sewer composites via autosampler (24 or 48 subsamples). | Influent from six WwTWs serving large urban areas across Israel (population high = 462670, low = 56943). 4/6 WwTWs received healthcare input (hospitals and nursing homes). | Microbroth dilution (Phoenix) susceptibility testing (antibiotics not provided). RT-PCR for detection of blaKPC and blaNDM-1. | All blood cultures, general cultures and rectal swabs received by one major hospital clinical microbiology laboratory in 2013 (semi-contemporaneous) Sample size not reported. | Six WwTW influent samples and 10 sewer system samples. Total number of isolates cultured or tested unclear: approximately 560 tested and 112 CRE included in analyses. | None conducted | WwTW samples and clinical isolates shared the same most commonly isolated blaKPC-carrying species (K. pneumoniae). They also shared the three other most commonly isolated blaKPC species but at different rates. Higher prevalence of MRSA and VRE in hospital wastewater and the receiving WwTWs than in domestic wastewater. | High |
| Moradigaravand et al. 2018 | UK | Comparison of resistance gene prevalence in C. difficile isolated from hospital patients and wastewater treatment works both downstream and unconnected to hospitals. | Composites of two consecutively collected 0.5L grabs from both influent and effluent. | Influent and effluent from 20 conventional WWTWs where 10 received hospital effluent and 10 did not. | Single isolate WGS. | 70 clinical isolates from patients with C. difficile-associated diarrhoea. | 40 wastewater samples and 186 wastewater isolates analysed. | None conducted | Low overall prevalence of AMR genes, most common being ermB (13%) and tetM (7%). | High |
| Mourkas et al. 2019 | Spain | Comparison of wastewater Campylobacter isolates and clinical isolates. | Grab samples” collected in sterile containers”. | Effluent from one site of “urban effluents”. | Microbroth dilution, WGS of subset. | Campylobacter isolates associated with campylobacteriosis cases from sewershed hospitals (n=152). | 50 wastewater samples and 53 wastewater isolates. | Sum of core genes used to create pangenome. Neighbour joining clustering method used to generate phylogenetic trees. | Phenotypic resistance prevalence of individual antibiotics tested were similar between human and sewage isolates – both groups showed high prevalence than the other in specific antibiotic comparisons. Genotypic comparisons showed human isolates generally had higher prevalence of the AMR genes tested. | High |
| Ojer-Usoz et al. 2017 | Spain | Comparison of resistance to individual antibiotics and presence of resistance genes in ESBL E.coli isolated from wastewater and hospital patients. | Not reported. | No details reported. | Microbroth dilution for 35 antibiotics and PCR for 5 AMR genes. | 130 clinical isolates of mixed origin provided by hospital routine diagnostics. | 279 wastewater samples and 132 wastewater isolated analysed. | R × C contingency test or Fisher’s exact test. | Similar resistance prevalence to individual antibiotics across compartments for ~half of antibiotics tested, differences with some significant for the rest - both higher and lower prevalence seen in wastewater compared to human compartment. Very similar presence of bla genes detected across compartments human (112/130) and wastewater (117/132) but varying specific gene distribution - CTX-M-15 being notably higher in human isolates (39.2% vs 11.1%). | High |
| Oravcova et al. 2017 | Czech Republic | Comparison of resistance to individual antibiotics and presence of resistance genes in VRE isolated from wastewater and hospital patients on oral antibiotic treatments. | Moore swab method exposing a cotton swab to wastewater effluents for 7 days. | Effluent from a large urban WWTW receiving effluent from multiple hospitals and long term care facilities. | Disk diffusion for 14 antibiotics and PCR for 14 AMR genes. | 33 isolates from rectal swabs collected from patients with current or previous oral antibiotic therapy. | 37 wastewater samples and 49 wastewater isolates analysed. | None conducted. | Presence of identical isolates from the WwTW and hospitalized patients, with presence of vanA-enterococci in WwTW outflows. All isolates cross compartments possessed vanA but none harboured vanB. Similar phenotypic resistances to individual antibiotics seen across WwTW and clinical isolates but varying prevalence of different genotypic AMR determinants. | Low |
| Pärnänen et al. 2019 | Portugal, Spain, Ireland, Cyprus, Germany, Finland, and Norway | The relative abundance of targeted AMR genes in STW influent/effluent collected across multiple European countries compared with AMR surveillance data from the EARS-Net database and national human antibiotic consumption amounts. | 24H composite samples (24 hourly sub samples totalling 1-2L) collected on three consecutive days (Tuesday, Wednesday and Thursday). | Influent and effluent from 13 WwTWs from seven countries. Median PE 173839 (IQR: 289906) and flow 33032.5 m3/d (IQR: 100529). Hospital sewer input described for 7/13 WwTWs. | DNA extraction (PowerWater Kit) and qPCR array with 384 primer sets targeting AMR genes and mobile genetic elements. | EARS-Net clinical AMR surveillance data from 30 European countries, specifically the 2017 report for 2013-2016 describing “a north-to-south and a west-to-east gradient is evident in Europe”. | 168 wastewater samples. 142/168 underwent qPCR (92 effluent and 50 influent). | PCoA using the Bray-Curtis dissimilarity index. | Wastewater ARG distribution consistent with country-level gradient from EARS-Net report. Relative abundance of ARG classes reflects country-level antibiotics consumption but more insertion sequences in low-use countries. Effluent antibiotics concentrations and primary care antibiotics consumption not associated to ARG abundance. | High |
| Pehrsson et al. 2016 | Peru, El Salvador | Resistomes and functional metagenomics of human faecal samples compared to wastewater collected from STW serving the human community sampled. | Samples collected in sterile containers. | Influent and effluent from one WwTW serving population of 700,00 including two shanty towns of low-income families. | Metagenomic DNA extracted from pellet via in-house phenol-chloroform bead beating method. Small-insert shotgun expression libraries created (pZE21 in E. coli DH10B), colony libraries selectively screened and pooled for DNA extraction. Metagenomic inserts amplified (PCR) and sequenced (HiSeq 2000). Assembly with PARTFuMS and annotation with Resfarms. Metagenomic DNA sheared (300–400 bp) and sequenced (HiSeq/NextSeq 2 × 150 PE). ShortBRED24 used to quantify abundance of AMR genes in the metagenomes based on generated functional metagenomics, CARD, human faecal metagenome assemblies, Lahey b-lactamase database. | Human faecal samples from shanty town serving WwTW sampled - collected semi-contemporaneously with wastewater. 5.7 ± 3.0 individuals per house sampled. Up to 44 faecal samples analysed. | 24 wastewater samples (12 influent, 12 effluent). Metagenomics on 22 wastewater samples after dropping low-yield extracts. Functional libraries created from 31 human samples from four households. Metagenomic libraries created from 44 human faecal samples. | Non-parametric Student’s t-tests with Bonferroni correction. | Street-access wastewater and WwTW influent resistomes were equally similar to human-associated resistomes. Street-access wastewater was more similar to influent than human faeces in terms of bacterial composition. All wastewater had higher phylogenetic diversity and more AMR proteins per sample than the human faeces. Drug efflux antibiotic resistance mechanisms were higher overall in human faecal vs wastewater resistomes. Investigating genetic context of cosmopolitan AMR proteins showed network of gene sharing between all sample metagenomes. | High |
| Pignato et al. 2010 | Italy | Prevalence of ampicillin resistance between E.coli and Salmonella isolates cultured from wastewater compared to Salmonella isolates cultured from symptomatic hospitalized patients. | Composite sampling (10 subsamples). | Influent and effluent from two WwTWs serving separate towns with hospital input for one site. Median PE 21500 (IQR 16000) and flow m3/d 3801.6 (IQR 3110.4) | Selective media at cut-off points to indicate resistance for eight antibiotics. Disk diffusion for subset of isolates (66) against 32 antibiotics to determine resistance patterns. PCR of four bla genes and class 1 integron gene cassettes. | 274 Salmonella isolates cultured from faeces of hospitalized patients with gastroenteritis at hospital served sampled WwTW during wastewater sampling period. | 108 total wastewater samples from which 64 Salmonella isolates cultured. 273 previously cultured E. coli included. | None conducted | Resistance prevalence in Salmonella isolates was of the same order in isolates from wastewater and clinical samples. Salmonella resistance profiles were similar between sample sources according to serovar. Specific six-drug pattern observed with a similar frequency in in Salmonella serovar Typhimurium isolates from wastewater and clinical specimens. Persistent isolation of suspected (not phage-typed confirmed) Salmonella DT104 endemic clone accounting for 40% of human salmonella isolates in the study country. Suspected DT104 represented 50% of wastewater isolates but only 22.2% of clinical isolates. Another potential clone with different restricted resistance pattern also appeared to be endemic in the same population. | High |
| Pot et al. 2020 | Guadeloupe | Comparison of ESBL Enterobacter cloacae from wastewater and sewershed clinical infections. | Grab samples (500ml) | Influent and effluent from 1 large WwTW treating hospital and urban wastewater - activated sludge process. | Selective media, disk diffusion, subset underwent WGS. | ESBL-producing Enterobacter cloacae associated with clinical infection (n=36). | 20 wastewater samples and 63 wastewater isolates. | Maximum likelihood phylogenetic tree. Temporal analysis via linear regression between root-to-tip and isolation year. | Focussing on wastewater-human component of the one health study: ESBL E. coli strains isolated from wastewater mirrored the prevalence in human clinical isolates. Regional ESBL-producing E. coli ST114 divided into two lineages both including wastewater and clinical isolates. | Low |
| Rahimi et al. 2014 | Iran | MRSA isolates collected during the same sampling period from sewage and human clinical infections compared using biochemical fingerprinting, SCCmec typing and ASTs. | Three grab samples collected in sterile 250 ml bottles. | Urban WwTW west of Tehran. | MIC by E-test for two antibiotics. AST by disk diffusion for 16 antibiotics. PCR detection of mecA, nucA, SCCmec (eight types/subtypes) and ccr (four types). | 489 clinical samples (wound, urine, sputum, blood, CSF, nose and abscess) from hospitalized patients collected during the same year as wastewater sampling. | Three wastewater samples from which 653 S. aureus cultured. | None conducted | Higher rates of multi-drug resistance in wastewater isolates versus clinical. Wastewater isolates also had higher single resistance rates to five other antibiotics. Homogeneity in biochemical typing consistent with contemporaneous outbreaks and dissemination of clones. | High |
| Raven et al. 2019 | United Kingdom | Genomic comparison of STW E.coli isolates (with and without hospital sewage input) to E.coli blood isolates from hospitalised patients in the same region preceding and during STW sampling period. | Composite (two subsamples) of consecutive 500 ml samples. | Influent and effluent from 20 WwTWs where 10 received hospital waste and 10 did not. 15 urban and 5 rural sites. Median PE 47957.5 (IQR 127521) and flow m3/d 27043.2 (IQR 48621.6). | Microbroth dilution (Vitek2) AST testing/phenotypic carbapenem test. DNA extracted (Qiagen QIAxtractor) and sequenced (Hiseq2000). AMR genes identified with ARIBA and ResFInder. Mobile genetic elements encodings five bla genes identified using in silico PCR approach. | Genomes of 437 E.coli isolates from hospital patients blood cultures (13/437 overlap with wastewater sampling, 424/437 are prior during 2006-2013). | 40 wastewater samples from which 394 E. coli isolates sequenced. Total of 388 in data analysis after dropping 6 low quality sequences. | Phylogenetic analysis of ST131 based on core genome SNPs. | Top three most common ST types from clinical blood cultures present in most WwTWs sampled (both receiving and not receiving hospital waste). Most prevalent ESBL gene from clinical samples was detected in all WwTWs. Prevalence of most common ESBL genes approximately reflected between wastewater and clinical samples although higher in wastewater. Majority of AMR gene variants identified were shared between groups. No detection of carbapenem or colistin resistance, consistent with very low resistance to these antibiotics in study area. | Low |
| Reinthaler et al. 2012 | Austria | Prevalence of antimicrobial resistant E. coli and resistance patterns of patient isolates collected in 2000 and 2009 compared to sewage sludge isolates collected during the same period. | Grab samples using sterile wide-mouth bottles. | Activated sludge from five WwTWs with no sewer input from hospitals. | Microbroth dilution (Vitek2) for identification and susceptibility testing to 15 antibiotics. | Random subset (500) of E. coli collection from patient samples (urine, sputum, stool, wound, skin and respiratory tract) taken by general medical practitioners during wastewater sampling periods. | 55 total wastewater samples from which 356 E. coli isolates cultured. | None conducted | Most commonly isolated resistances shared between patient and sludge isolates but lower overall prevalence in sludge. Increase in individual resistances over time seen in both groups. | High |
| Saifi et al 2009 | Iran | Assessing clonality and resistance prevalence of gentamicin-resistance Enterococcus faecium isolates isolated from clinical and STW samples. Both STWs and clinics in the same city but overlap unclear but associated in time. | Not reported | Influent from three WwTWs located in different parts of Tehran. | Disk-diffusion and microbroth dilution for antimicrobial susceptibility testing (10 antibiotics). PCR for five genes (aac (6 ′)‐Ie‐aph (2 ″)‐Ia, aph (2 ″)‐Ib , aph (2 ″)‐Ic, aph (2 ″)‐Id and ant (4 ′)‐Ia ). | 320 enterococcal isolates cultured from urine and wound samples from six outpatient clinics during sampling period. | Unclear total wastewater samples and isolates: total of 106 high-level gentamicin-resistant isolates (clinical = 48, WwTW = 58) | None conducted | Clinical isolate resistance rates higher than wastewater isolates. Individual resistances match national prescribing levels. Low PFGE type overlap between groups. | High |
| Talebi et al. 2008 | Iran | Prevalence of resistance to single/combinations of antibiotics in clinical VRE and wastewater VRE isolates. PFGE and biochemical fingerprinting patterns in clinical VRE compared to wastewater VRE isolates. | Grab samples using 250 ml sterile wide-mouth bottles. | Three urban WwTWs located in different areas of Tehran. | VRE isolated by classical culture-based methods. PCR for detection of vanA, vanB. AST disk diffusion and E-test for eight antibiotics. | Enterococcus species isolated from clinical samples (450) (predominately urine) from three major hospitals. | Six wastewater samples from which 593 enterococcal isolates cultured. | None conducted | MICs higher in human isolates compared to wastewater isolates. Multi-drug resistance detected at the same prevalence between sample groups. Human isolates showed higher PFGE type diversity consistent with no reported contemporaneous outbreaks compared to lower diversity in wastewater isolates. | High |
| Urase et al. 2020 | Japan | Comparison between wastewater-isolated CTX-R E.coli and MPEM-R Enterobacteriaceae prevalence compared to JANIS national surveillance data. | Not reported | Effluent “after chlorination” from 12 WwTWs in Tokyo and suburbs. Details: 9 receive hospital and use activated sludge treatment, 2 use oxidation ditches in rural areas, 1 is a community plant with no medical facilities. | Selective chromogenic media incubated at both 37 and 42C - isolates identified by 16S rRNA sequencing, disk-diffusion AST. | Resistance prevalence from Japan Nosocomial Infections Surveillance (JANIS 2019) - both inpatients and outpatients. | 17 wastewater samples, 605 E.coli isolated tested against CTX, 976 Enterobacteriaceae tested against MEPM. | None conducted. | Lower resistance prevalence in effluent wastewater isolates (5.7%) compared to clinical surveillance data of both in-(27.5%) and outpatients (17%). | High |
| YoungKeun et al. 2015 | Sweden | Resistance rates of E. coli isolated from STW influent, effluent and hospital effluent in 2013-2014 compared to national resistance rates of human blood isolates from 2007-2012. | 24H WwTW "continuous" sampling via autosampler. 4H hospital composites comprising 50 ml grab samples every 10-15 minutes (16 subsamples). | Influent from WwTW with PE >750,000 and flow 250,000 m3/d. Receives hospital input (800 bed) but no significant agricultural sewer input. | Microbroth dilution (PhenePlate) with 10 antibiotics. Microarray targeting six ESBL and six plasmid-mediated AmpC genes. | Resistance data from EARS-Net European database on human blood isolates limited to studied country for the years 2007-2012 (preceding wastewater sampling). | 17 influent samples from which 1326 E. coli isolates cultured. Three effluent samples from which 117 E. coli isolates cultured. Six hospital effluent samples from which 451 E. coli cultured. | None conducted | Increasing resistance prevalence of WwTW isolates reflect increase in resistance of human blood isolates. Lower prevalence of resistance in WwTW isolates compared to hospital effluent. | High |
| Zaheer et al. 2020 | Canada | Comparison of wastewater-isolated Enterococcus spp. and clinical isolates. | Composite of influent and effluent (1L). | Influent and effluent (post-grit tank) from 2 WwTWs – with agriculture input. | Selective culture, disk diffusion and WGS of subset. | Clinical isolates - mostly non-sterile sites but blood, other tissue and rectal swabs (subset for WGS = 299). | 43 wastewater samples and subset of 169 isolates underwent WGS. | Phylogenetic analysis via single nucleotide variate phylogenomics of core genes using unassembled sequence data. | MDR prevalence in clinical isolate was much higher than wastewater isolates. VRE was only detected in clinical isolates. The majority of wastewater and human isolates were closely related when compared to other one health samples, and harboured similar resistance genes. Phylogenetic clustering showed clinical and wastewater isolates grouped together separately compared to other one health samples indicating surveillance potential of human carriage. | Low |
| Zarfel et al. 2013 | Austria | AST, biochemical phenotypes, plasmid replicon types and bla gene presence of ESBL E. coli isolates from STW sludge compared to isolates from hospital patients within STW catchment and collected in the same sampling period. | Grab samples collected using sterile wide-mouth bottles. | Activated sludge from five WwTWs "PE range <10,000 to >100,000 and flow rate range 100–1200 L/min". | AST for 11 antibiotics using microbroth dilution (Vitek). Three additional antibiotics via disk diffusion. CLSI confirmation of ESBL. PCR for five b-lactamase gene families. | 50 ESBL E. coli isolates from hospitalized patients with UTIs collected contemporaneously with wastewater. | 30 sludge samples from which 50 ESBL E. coli isolated. | None conducted | Most prevalent ESBL genes detected were shared across wastewater sludge and UTI isolates. TEM-1 gene detected at similar rates between groups. Both groups shared the same level of resistance pattern diversity. UTI isolates showed reduced phenotypic variation but both groups clustered together with no distinction. Higher resistance rates against clinical antibiotics seen in UTI isolates but not for non-clinical antibiotics. | Unclear |
