# Supplementary material for: Systematic review of wastewater surveillance of antimicrobial resistance in human populations
Source: Environ Int. 2022 Apr;162:107171. doi: 10.1016/j.envint.2022.107171 (PMC8960996; doi:10.1016/j.envint.2022.107171)
Supplement: Supplementary data 8 [file mmc8.docx]

| **Citation** | **Study aims** | **Wastewater sampling strategy** | **Targeted organism and resistances** | **Wastewater handling and storage** | **Sampling season and weather reporting** |
| --- | --- | --- | --- | --- | --- |
| Adator et al. 2020a | To investigate ESBL E. coli from a one health continuum linked to beef production. | Longitudinal sampling over 24 months – frequency of visits not reported. | ESBL E. coli. | Not reported. | All seasons - weather conditions not reported. |
| Adator et al. 2020b | To examine similarities between AMR patterns/prevalence of E.coli along a one health continuum. | Longitudinal over 24 months with a two-monthly interval. | ESBL E.coli. | Not reported. | All seasons - weather conditions not reported. |
| Aljanaby et al. 2018 | To investigate the relationship between virulence genes and antibiotic resistance in pathogenic bacteria isolated from clinical and environmental sources. | Longitudinal sampling over 10 months - interval not reported. | P. aeruginosa. | Not reported. | Not reported. |
| Colomer-Lluch et al. 2014 | To evaluate the abundance of several ARGs in bacteria and bacteriophage DNA derived from wastewater collected from distant sources with differing socio-economic and cultural characteristics, climate and geographic background. | Sampling between 2011 and 2014 with unclear interval. | Metagenomics targeting blaTEM, blaCTX-M-1, blaCTX-M-9, mecA, armA, qnrA, qnrS, sul1. | Frozen at -80°C and stored in dry ice before filtration (0.45μm) and processing. | Not reported. |
| Golle et al. 2017 | To evaluate possible overlap between clinically relevant and environmental strains and determine transfer from wastewater to WWTW. | Longitudinal sampling between Jan-Dec 2014 with one month interval - 12 timepoints. | Carbapenem-resistant P. aeruginosa targeting 1411 carbapenemase genes. | Refrigerated for up to 6H before filtration (0.45μm). | All seasons - weather conditions not reported. |
| Gouliouris et al. 2019 | To generate indirect evidence for the extent to which healthcare-associated E. faecium is disseminated in the community. | Snapshot sampling between June 2014 and January 2015. | Vancomycin-resistance E. faecium. Non-limited genotyping. | Sodium thiosulphate- based preservation. | Summer, autumn, winter - weather conditions not reported. |
| Haghi et al. 2019 | To investigate potential transmission of VRE from wastewater to humans by sampling VRE in wastewater and faecal samples of healthy carriers. | Longitudinal samples "collected randomly" from June to October 2017. Unclear timepoints. | Vancomycin- resistant Enterococcus spp. | Kept at 4°C until processing - no other details reported. | Summer - weather conditions not reported. |
| Hendriksen et al. 2019a | To monitor circulating pathogens and AMR genes via metagenomics of urban wastewater with comparison to other concurrent disease surveillance. | Longitudinal sampling every Monday and Wednesday between June 16 and August 26 2014 - 42 timepoints. | Metagenomics – non-limited genotyping. | Samples whole frozen at -80°C within 2 hours of collection before shipment without coolant to authors. Samples were thawed for 48H at 4°C prior to processing. | Summer - weather conditions not reported. |
| Hendriksen et al. 2019b | To characterise bacterial resistomes and explain AMR variation in untreated wastewater from sites sampled on a global scale. | Snapshot sampling between 25 Jan and 5 Feb 2016. | Non-limited | Samples frozen at -80°C (48H) before shipping to authors without cold chain. Samples then thawed at 20 for 12H before pelleting via centrifugation and storage at -20 or -80°C before processing. | Winter, spring summer - weather conditions reported as air temperature at time of sampling and brief description (sunny/cloudy/rainy/foggy/snowy). |
| Huijbers et al. 2020 | To evaluate relationship between AMR prevalence in E.coli from wastewater and clinical samples with traditional clinical surveillance comparison. | Snapshot sampling, one timepoint per WWTW between Dec 2016 and Feb 2017. | E. coli resistant to ampicillin, ciprofloxacin, cefotaxime, ceftazidime, gentamicin, meropenem. | Cooled (4°C) until processing 12H after collection. | All seasons – based on reference flow provided during dry conditions, majority of sampling was during wet conditions. |
| Hutinel et al. 2019 | To investigate the relationship between E. coli resistance rates in WWTW and hospital wastewater compared to clinical samples. | Longitudinal samples collected in 2016 with 1-2-month interval. Up to 12 timepoints. | E. coli - no specific resistance targeted. | Kept at 4°C and processed within three hours of collection. | All seasons - weather conditions not reported. |
| Jakobsen et al. 2008 | To investigate the potential spread of gentamicin resistant (GEN-R) E. coli isolates or GEN-R determinants from a Danish university hospital to wastewater. | Longitudinal monthly sampling between October 2002 - August 2003 - 11 timepoints. | Gentamicin-resistant E. coli (aac(3)-II, aac(3)-IV, ant(2″)-I, armA, aac(3)-I, aac(3)-III). | Not reported | All seasons - weather conditions not reported. |
| Jorgensen et al. 2017 | To investigate possible transmission routes of ESBL-E.coli by comparing isolates from various niches (freshwater lakes, saltwater basins, WWTW and UTIs). | Longitudinal sampling (May 2010, two visits in June, Aug, and Sep) - 5 timepoints. | Third generation cephalosporin-resistant E. coli (blaCTX-M, blaTEM, blaSHV, blaNDM, blaKPC). | Filtered - no other details reported. | Summer - weather conditions not reported. |
| Karkman et al. 2020 | To characterise bacterial resistomes and explain AMR variation in untreated sewage from sites sampled on a global scale. | As described for GSSP. | Non-limited metagenomics exploration – phenotypic clinical surveillance data limited to four antibiotic classes: aminopenicillins, fluoroquinolones, third generation cephalosporins and aminoglycosides | As described for GSSP. | As described for GSSP. |
| King et al. 2020 | To investigate the AMR profiles of Klebsiella spp. from hospitals, hospital effluents and WWTWs. | Longitudinal: two timepoints over three months. | Klebsiella spp. with focus on ESBLS and carbapenemase resistance. | Transferred on ice and processed within 3H of collection. | Winter and Spring - weather conditions not reported. |
| Meir-Gruber et al. 2016 | To evaluate the population carriage of pan-resistant bacteria and to establish a convenient and fast method for wastewater sampling. | Snapshot sampling between Apr 2012 and Nov 2013. | Carbapenem, methicillin and vancomycin resistance in Enterobacteriaceae, S. aureus and enterococci spp (blaKPC, blaNDM-1). | Not reported | Not reported |
| Moradigaravand et al. 2018 | To investigate C. difficile in wastewater treatment plants and compare to clinical isolates from the same geographical region to identify if wastewater treatment plants are potential reservoirs. | Snapshot sampling of individual sites over 8 months - interval between sites not reported. | C. difficile, non-limited genotypic detection using srst2 package database. | Mixing of wastewater samples with sodium thiosulphate, filtration and alcohol shock for selected samples. | Summer, Autumn, Winter - weather not reported. |
| Mourkas et al. 2019 | To compare genomes of AMR campylobacter from multiple niches. | Longitudinal sampling over 36 months – frequency of visits not reported. | Campylobacter with high resistance to ciprofloxacin, tetracycline, erythromycin, streptomycin and gentamicin. | Stored at 4°C – no other details reported. | Not reported |
| Ojer-Usoz et al. 2017 | To characterise the clonal diversity of ESBL-E. coli from food, humans and environmental niches. | Longitudinal sampling - length and interval not reported. | ESBL E. coli and presence of blaCTX-M-14, blaCTX-M-1, blaCTX-M-15, blaTEM-42, blaSHV-12. | Not reported. | Not reported. |
| Oravcova et al. 2017 | To investigate if VRE is discharged by the sampled WWTW, if gulls are colonised by effluent VRE if present, and compare to admitted hospital patients. | Longitudinal sampling over 10 months - interval not reported. | VRE - Presence of vanA, vanB, vanC, tet(K), tet(L), tet(M), tet(O), erm(A), erm(B), mef(A), ant(4′)-Ia, aac(6′)-Ie-aph(2″)-Ia, and aph(3′)-IIIa. | Processed within 30m of collection. | All seasons - weather not reported. |
| Pärnänen et al. 2019 | Trans-European antibiotic resistance surveillance in WWTWs - an initial survey for basing future regular surveillance protocols. | Longitudinal sampling with three timepoints over one year (autumn 2015, spring and autumn 2016). Four sites missing timepoints. | Targeting 229 resistance genes (not organism specific). | Filtered (0.22μm) and stored at -80°C before processing. | Autumn, spring - weather conditions reported as average annual air temperature based on "timeanddate.com". |
| Pehrsson et al. 2016 | To map resistance gene dissemination between humans and their environments with focus on low-income populations. | Longitudinal sampling between May 2012 and Jan 2013 with 1-2-month interval. Up to nine timepoints. | Non-limited. | Frozen (−20 to −80°C) until shipment to authors where samples were stored at −80°C before filtration (0.22 μm). | All seasons - weather conditions not reported. |
| Pignato et al. 2010 | To investigate whether wastewater isolates can be a useful/easy for epidemiological monitoring of AMR prevalence in Salmonella clones circulating in the human population. | Longitudinal sampling between Jan-Dec 2004 with 0.5-1 month interval. Up to 24 timepoints. | Ampicillin-resistant Salmonella spp. and E. coli (blaTEM, blaSHV, blaPSE-1, blaOXA-1 group). | Refrigerated (4°C) before filtration and processing. | All seasons - weather conditions not reported. |
| Pot et al. 2020 | One health study to assess ESBL Enterobacter cloacae between WWTWs, humans and animals. | Longitudinal: Ten timepoints between April 2018 and March 2019. | ESBL Enterobacter. | Stored at 4°C and processed within 4H of collection. | All seasons - weather conditions not reported. |
| Rahimi et al. 2014 | To determine epidemiological relatedness between MRSA strains isolated from wastewater and human infections. | Snapshot samples collected in 2010 - no other details. | MRSA (mecA). | Cold chain transport before filtration (0.45μm) and processing. | Not reported. |
| Raven et al. 2019 | To evaluate whether the sequencing of E. coli isolated from WWTWs could support surveillance of pathogenic E.coli lineages and AMR genes in a specific region. | Snapshot sampling between June 2014 and January 2015. | Third generation cephalosporin-resistant E. coli with non-limited genotype targeting. | Sodium thiosulphate- based preservation before filtration. | Summer, autumn, winter - weather conditions not reported. |
| Reinthaler et al. 2012 | To assess transmission of antibiotic resistances from human sources into the environment. | Two sampling periods of six months each divided by ~nine years (five days between Apr - Sep 2000) and (six days between Apr - Sep 2009). | E. coli - no specific resistance targeted. | Kept refrigerated (4-8°C) for up to 24H before processing. | Spring, summer, autumn - weather conditions not reported. |
| Saifi et al 2009 | To analyse and compare clonal diversity of gentamicin-resistant E. faecium in clinical and WWTW samples to assess release into the environment. | Longitudinal sampling with unclear interval between September 2005 - September 2006. Unclear timepoints. | Gentamicin-resistant E. faecium (6 ′)‐Ie‐aph (2 ″)‐Ia, aph (2 ″)‐Ib , aph (2 ″)‐Ic, aph (2 ″)‐Id, ant (4 ′)‐Ia). | Filtration (0.45μm) - no other details reported. | Not reported. |
| Talebi et al. 2008 | This study was undertaken to determine the genetic relatedness between the VRE isolates of wastewater and human urinary tract infection isolates. | Snapshot sampling during 2005 - no other details reported. | Vancomycin-resistant E. faecium (vanA and vanB). | Kept refrigerated before filtration (0.45 μm) and processing. | Not reported. |
| Urase et al. 2020 | Comparison of ESBL E. coli and CRE in treated wastewater to national surveillance data on nosocomial infections. | Snapshot and longitudinal: One timepoint for 9 WWTWs, four timepoints for 1 WWTW and two timepoints for 1 WWTW - taken 2018-2019. | E. coli possessing ESBLs, and CREs. | Sampled collected in the morning and processed in the afternoon – no cool chain mentioned. | All seasons – samples taken to avoid rain events. |
| YoungKeun et al. 2015 | To provide evidence for AMR wastewater surveillance for monitoring resistance trends and as an early warning system. | Longitudinal sampling with five timepoints over one year: Jan, Feb, July 2013, Jan 2014, Feb. | E. coli (blaCTX-M, blaTEM, blaSHV, blaNDM, blaKPC). | Kept at 4°C and analysed within 4H. | Winter, summer - weather conditions not reported. |
| Zaheer et al. 2020 | To assess contributions of beef cattle to AMR in environmental niches and transmission chains to humans. | Longitudinal sampling over 24 months – frequency of visits not reported. | Enterococcus resistant to critically and highly important antibiotics. | Not reported. | All seasons - weather conditions not reported. |
| Zarfel et al. 2013 | To investigate wastewater environmental samples to understand transmission of AMR to humans. | Longitudinal monthly sampling between Feb and July 2009. Six timepoints. | Third generation cephalosporin-resistant E. coli (blaTEM, blaSHV, blaCTX-M, blaVEB and blaGES). | Kept at 4–8°C for up to 24H before processing. | Winter, spring, summer - weather conditions not reported. |
