# Supplementary material for: Systematic review of wastewater surveillance of antimicrobial resistance in human populations
Source: Environ Int. 2022 Apr;162:107171. doi: 10.1016/j.envint.2022.107171 (PMC8960996; doi:10.1016/j.envint.2022.107171)
Supplement: Supplementary data 9 [file mmc9.docx]

**Studies without extractable data – full descriptive summary**

*Targeted qPCR-based studies*

Two studies performed direct AMR gene detection using qPCR of either 229 (Pärnänen et al., 2019) or eight AMR genes (Colomer-Lluch et al., 2014); both reported a relationship between wastewater AMR and national AMR data. Pärnänen et al. conducted a longitudinal survey of 13 European WwTWs across seven countries compared with contemporaneous surveillance data from EARS-Net. The relative abundance of influent AMR genes clustered significantly into two distinct country groups coinciding with high and low antibiotic consumption, with AMR gene distributions mirroring EARS-Net (i.e. human AMR surveillance)-described north-to-south and west-to-east geographic gradients. A higher relative abundance of most AMR gene classes was observed from high antibiotic consumption countries except for tetracycline and macrolide-lincosamide-streptogramin B AMR genes. The study also performed counts of antibiotic-resistant culturable bacteria on samples but showed no significant correlation between counts and AMR gene quantification.

Colomer-Lluch et al. compared wastewater samples in Tunisia and Spain, and their respective national AMR surveillance data. Intra-country comparisons showed the most commonly recovered beta-lactamase gene (*bla*_TEM_) from wastewater was also most commonly reported in clinical surveillance. This was also true of the quinolone resistance genes *qnr*S and *qnr*A but not for CTX-M-9 group ESBL genes, where a higher prevalence was identified in wastewater. Inter-country comparisons also highlighted higher AMR gene detection in samples from Spain coinciding with higher antibiotic use, and high *mec*A prevalence thought to be potentially consistent with high-pig farming densities.

*Metagenomic-based studies*

Four studies employed metagenomics to identify potential wastewater-human AMR associations. Two of these studies appeared to demonstrate an association while the other two were inconclusive. Hendriksen et al. conducted a large-scale cross-sectional survey of global wastewater and compared resistomes to multiple epidemiological variables (Hendriksen et al., 2019b). This study showed total AMR gene abundances from a given site/country correlated with sanitation and general health metrics (with up to 89% of observed resistome variation significantly explained regardless of within-sample AMR gene diversity). A high human development index (i.e. generally high-income countries) was linked to significantly lower abundance of AMR genes in wastewater metagenomes.

Karkman et al. compared wastewater resistomes (generated by Hendriksen et al.) to local clinical surveillance data extracted from surveillance networks (EARS-Net, CAESAR and ResistanceMap) (Karkman et al., 2020). A beta regression model showed relative abundance of AMR genes could partially explain clinical resistance patterns but focussing on individual classes of resistance only improved model fit for aminopenicillin resistance. The best models were produced by incorporating country-level socioeconomic factor data (GDP, basic sanitation and proportion of urban population), which improved model fit for all resistance classes except aminoglycoside resistance.

The first inconclusive metagenomics-based study conducted longitudinal surveillance (Hendriksen et al., 2019a) comparing resistomes generated from drainage ditch wastewater and contemporaneous local surveillance data consisting of household morbidity and healthcare usage. Significant increases/decreases in resistome read abundance were not associated with contemporaneous increases in reported diarrhoea or clinical faecal isolates. However, authors highlighted non-significant increases in pathogen read abundances appeared to coincide with increased reported illness/clinic visits, possibly reflecting a lack of sensitivity to detect significant changes in read abundances.

The second inconclusive study was a metagenomic and functional metagenomic longitudinal evaluation of wastewater and human faecal samples collected from individuals within the WwTW catchment (Pehrsson et al., 2016). Characterisation of resistomes, including the use of AMR gene networks, showed significantly higher phylogenetic species diversity and abundance of AMR proteins in wastewater (WwTW influent, street-access wastewater) compared to faecal samples. Faecal samples were significantly enriched for drug efflux mechanisms whereas wastewater samples contained more aminoglycoside acetyltransferase, class D β-lactamase, and dihydrofolate reductase genes. However, extensive sharing of sulphonamide-resistance conferring AMR genes was detected between sample sets indicating possible resistance-specific relationship.

*Mixed-approach and phenotypic-only studies*

Two studies used mixed approaches combining phenotypic AST with qPCR (2-targets) (Meir-Gruber et al., 2016) and single isolate WGS (Gouliouris et al., 2019); one study used a phenotypic approach only (YoungKeun et al., 2015). All three studies appeared to show a wastewater-human AMR association. Meir-Gruber et al. compared prevalence of *bla*_KPC_/*bla*_NDM-1_-carrying CREs, MRSA and VRE in WwTW influent and domestic sewer systems collected nationally over one year compared to contemporaneous clinical samples. The study found a limited observational association where wastewater and clinical isolates shared the same most commonly isolated *bla*_KPC_-carrying species (*K. pneumoniae*) in addition to the other top three isolated *bla*_KPC_ species.

The second mixed approach study (Gouliouris et al., 2019) compared wastewater *E. faecium* isolates from 20 WwTWs and isolates from clinical infection preceding and during study sourced from hospitals in the same region. They found wastewater and clinical isolates were phylogenetically interspersed, indicating mixing, with divergence events implying recent emergence and dissemination in the geographic region. Each WwTW sampled also contained genetically diverse populations but these remained comparable to diversity in national bloodstream infection isolates. Network analysis showed geographic clustering of WwTWs and bloodstream isolates, and linked bloodstream isolates to 9/20 WwTWs (three without hospital sewer input).

Lastly the phenotypic-only study (YoungKeun et al., 2015) compared prevalence of resistant *E. coli* isolated from WwTW influent, effluent and hospital effluent to national resistance prevalence of clinical blood isolates with a non-overlapping sampling period. The authors found a lower prevalence of resistance in WwTW isolates when compared to hospital effluent. An observational wastewater-human association was reported in the increasing resistance prevalence of WwTW isolates which may reflect increases in the clinical dataset.
